# Network meta‐analysis of medical therapy efficacy in more than 90,000 patients with heart failure and reduced ejection fraction

**DOI:** 10.1111/joim.13487

**Published:** 2022-04-12

**Authors:** Vincenzo De Marzo, Gianluigi Savarese, Lucia Tricarico, Sofia Hassan, Massimo Iacoviello, Italo Porto, Pietro Ameri

**Affiliations:** ^1^ Department of Internal Medicine University of Genova Genova Italy; ^2^ Division of Cardiology Department of Medicine Karolinska Institutet Stockholm Sweden; ^3^ Cardiology Unit Ospedali Riuniti di Foggia Foggia Italy; ^4^ Department of Medical and Surgical Sciences University of Foggia Foggia Italy; ^5^ Cardiology Unit Cardio‐Thoracic and Vascular Department IRCCS Ospedale Policlinico San Martino Genova Italy

**Keywords:** heart failure, mortality, outcomes, pharmacotherapy, prognosis, trial

## Abstract

**Background:**

Following the availability of new drugs for chronic heart failure (HF) with reduced ejection fraction (HFrEF), we sought to provide an updated and comparative synthesis of the evidence on HFrEF pharmacotherapy efficacy.

**Methods:**

We performed a Bayesian network meta‐analysis of phase 2 and 3 randomized controlled trials (RCTs) of medical therapy in HFrEF patient cohorts with more than 90% of the participants with left ventricular ejection fraction less than 45% and all‐cause mortality reported.

**Results:**

Sixty‐nine RCTs, accounting for 91,741 subjects, were evaluated. The step‐wise introduction of new drugs progressively decreased the risk of all‐cause death, up to reaching a random‐effects hazard ratio (HR) of 0.43 (95% credible intervals [CrI] 0.27–0.63) with beta blockers (BB), angiotensin‐converting enzyme inhibitors (ACEi), and mineralocorticoid receptor antagonist (MRA) versus placebo. The risk was further reduced by adding sodium–glucose cotransporter‐2 inhibitors (SGLT2i; HR 0.38, 95% CrI 0.22–0.60), ivabradine (HR 0.39, 95% CrI 0.21–0.64), or vericiguat (HR 0.40, 95% CrI 0.22–0.65) to neurohormonal inhibitors, and by angiotensin receptor–neprilysin inhibitor (ARNI), BB, and MRA (HR 0.36, 95% CrI 0.20–0.60). In a sensitivity analysis considering the ARNI and non‐ARNI subgroups of SGLT2i RCTs, the combination SGLT2i + ARNI + BB + MRA was associated with the lowest HR (0.28, 95% CrI 0.16–0.45 vs. 0.40, 95% CrI 0.24–0.60 for SGLT2i + BB + ACEi + MRA). Consistent results were obtained in sensitivity analyses and by calculating surface under the cumulative ranking area, as well as for cardiovascular mortality (information available for 56 RCTs), HF hospitalization (45 RCTs), and all‐cause hospitalization (26 RCTs).

**Conclusions:**

Combination medical therapy including neurohormonal inhibitors and newer drugs, especially ARNI and SGLT2i, confers the maximum benefit with regard to HFrEF prognosis.

## Graphical abstract

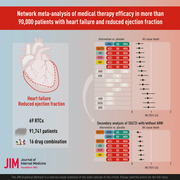

## Introduction

Three classes of disease‐modifying neurohormonal inhibitors—beta blockers (BB), angiotensin‐converting enzyme inhibitors/angiotensin receptor blockers (ACEi/ARB), and mineralocorticoid receptor antagonists (MRA)—represent the core of medical therapy for chronic heart failure (HF) with reduced ejection fraction (HFrEF) [1, 2]. Ivabradine and angiotensin receptor–neprilysin inhibitors (ARNI, i.e., sacubitril/valsartan) complement neurohormonal inhibitors, and pivotal randomized controlled trials (RCTs) and subsequent meta‐analyses showed that the use of ivabradine or ARNI together with neurohormonal inhibitors improves outcomes in HFrEF patients [3, 4].

In recent years, additional compounds have been tested for treatment of HFrEF. In particular, sodium–glucose cotransporter‐2 inhibitors (SGLT2i) [5, 6] and vericiguat [7] reduced the risk of the combined endpoint of cardiovascular (CV) death and hospitalization for HF (HHF) as compared with placebo and, consequently, received clinical approval from regulatory authorities.

The RCTs evaluating the new HFrEF medications have been conducted in parallel during the same periods. As a result, a measure of the overall effect as well as of the relative efficacy of the most recent drugs is lacking, which may delay their uptake by the medical community.

Against this background, we performed an updated systematic review and a network meta‐analysis (NMA), with the goal of summarizing RCT data into a framework encompassing direct and indirect comparisons of medical interventions for HFrEF [8].

## Methods

This NMA was registered in the PROSPERO database (ID: CRD42021228040) and performed according to the Preferred Reporting Items for Systematic Reviews and Meta‐Analysis (PRISMA) recommendations (Table S1) [9]. Full methods and the data not available within the article are provided as Supplementary Material.

### Search strategy

We systematically searched the MEDLINE, Embase, Scopus, and Cochrane Library databases for English‐language, peer‐reviewed publications of RCTs in HFrEF up to 30 November 2020, using the search strings "heart failure" and/or "randomized controlled trial" (Table S2). The references of the selected articles were also thoroughly screened.

### Eligibility criteria and data extraction

After identifying the phase 2 and 3 RCTs enrolling individuals with chronic HF and left ventricular ejection fraction (LVEF) less than 45%, we excluded those with more than 10% of subjects with at least 45% LVEF, which were not representative of the broad HFrEF population (i.e., investigating only specific subsets of patients) or for which there was no published information about the rates of all‐cause death in both the intervention and placebo or comparator arms. We also excluded the studies comparing different molecules of the same drug class (e.g., BB vs. BB).

Three investigators (V.D.M., L.T., and S.H.) independently reviewed the retrieved articles and extracted baseline patient characteristics and therapies, follow‐up duration, total numbers of patients, and outcome events in the arms of each RCT, and measures of relative risk (hazard ratios [HR]), if available. Disagreements were solved by involvement of another two investigators (P.A. and M.I.).

To account for concomitant treatments, the tested compound was considered as combined with other drugs if more than 50% of the patients took these medications [3, 4, 10].

### Analysis outcomes

The primary outcome was all‐cause death. The secondary outcomes of CV death, HHF, and all‐cause hospitalization were also investigated for those RCTs with data available.

### Assessment of risk of bias

We used the Cochrane Collaboration's tool to assess risk of bias in six prespecified domains: selection bias, performance bias, detection bias, attrition bias, reporting bias, and other bias [11].

Comparison‐adjusted funnel plots and Egger's regressions were employed to visualize publication bias, as previously described [12].

### Quality of evidence

The quality of evidence (high, low, or unclear) was examined according to the Grading of Recommendations, Assessment, Development, and Evaluation tool [13].

### Network meta‐analysis

The NMA comprised a fixed‐effects model and a more conservative random‐effects model within a Bayesian framework using R and JAGS software [14]. The Markov chain Monte Carlo method was used, running two chains with 200,000 iterations after a burn‐in of 100,000. Noninformative priors were used. Results of the random‐effects model are presented unless the fixed‐effects model resulted in a more parsimonious model.

The log mean/median follow‐up time was used to transform the probability of an event into a constant rate for an RCT trial arm by assuming an underlying Poisson process, and a log link was used to model the event rates.

Heterogeneity was measured through the I^2^ statistic and τ^2^ heterogeneity [15], and convergence was evaluated according to Gelman–Rubin–Brooks [16].

Consistency was assessed comparing direct and indirect evidence with the node‐splitting technique.

The probability that a treatment ranked among the most effective for the outcomes of interest was calculated as a surface under the cumulative ranking area (SUCRA) value between 0% and 100% [17].

The following sensitivity analyses were performed: use of a frequentist random‐effects approach with the DerSimonian–Laird estimator [18], sequential exclusion of the selected studies (leave‐one‐out analysis), and separation of the neurohormonal inhibitor and ARNI + BB + MRA groups in SGLT2i RCTs, although the proportion of participants on ARNI in DAPA‐HF and EMPEROR‐REDUCED was below the prespecified threshold of 50% (see Eligibility criteria and data extraction).

Metaregression analyses were carried out to determine whether exclusion of covariates with less than 30% missing values modified the goodness of fit of the original regression model, with a 5‐units deviance information criterion (DIC) reduction being considered suggestive for a goodness‐of‐fit improvement [19]. The same strategy was adopted to account for the risk of time bias, since the selected RCTs spanned 33 years.

The NMA was conducted in R environment (RStudio Desktop, version 1.2.5033) with *forestplot*, *gemtc*, *ggplot2*, and *netmeta* packages. We set statistical significance at *p* < 0.05 for the frequentist NMA.

## Results

The PRISMA flowchart depicting the search and selection of references is provided in Fig. S1, while the list of references included in the qualitative synthesis—but then excluded based on prespecified criteria—is given in Table S3.

**Fig. 1:**
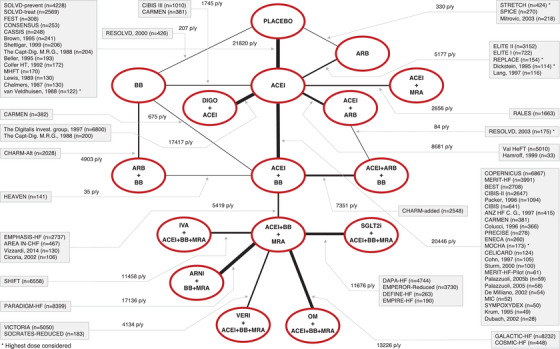
Network of the comparisons between different pharmacological treatments for the primary endpoint of all‐cause death. Each treatment (single drug or combination therapy) is represented by a node and is connected to the other treatments (either single drug class or a combination therapy), irrespective of the existence of head‐to‐head comparison. The thickness of the lines is proportional to the number of patients/years. Interventions with different molecules belonging to the same pharmacological class, for example, BB, were pooled together to form one node. ACEi, angiotensin converting enzyme inhibitors; ARB, angiotensin receptor blockers; ARNI, angiotensin receptor–neprilysin inhibitor; BB, beta blockers; DIGO, digoxin; IVA, ivabradine; MRA, mineralocorticoid receptor antagonists; OM, omecamtiv mecarbil; SGLT2i, sodium–glucose cotransporter 2 inhibitors; VERI, vericiguat.

A total of 69 RCTs were included in the NMA for the primary outcome of all‐cause death (Fig. 1). Figures S2–S4 display the diagrams for CV death (56 RCTs), HHF (45 RCTs), and all‐cause hospitalization (26 RCTs).

### Study and patient characteristics

Most RCTs were double‐blind, placebo‐controlled, and multicenter. The publication year was between 1987 and 2020. In the oldest RCTs, the investigational drugs (ACEi, BB, or ARB) were evaluated alone, in the absence of any other disease‐modifying HfrEF medication. Conversely, in the subsequent RCTs the interventions were always part of combination treatments of increasing complexity with the progressive adoption of additional drugs (Fig. 1 and Figs S2–S4).

The main features of the selected RCTs are summarized in Table 1.

**Table 1:** Characteristics of the RCTs included in the network meta‐analysis

						NYHA (%)									
RCT (year)	FUP (months)	Patients (*N*)	Arms	Age (years)	Males (%)	I	II	III	IV	LVEF (%)	Ischemic heart failure (%)	AF (%)	Diabetes (%)	Hypertension (%)	HHF (%)
Chalmers (1987)	3	87 43	Lisinopril Placebo	58	69	0	22	65	13	NA	NA	NA	NA	14	NA
CONSENSUS (1987)	12	127 126	Enalapril Placebo	70	71	0	0	0	100	NA	NA	50	22	21	NA
The Captopril–Digoxin Multicenter Research Group (1988)	6	104 96 100	Captopril Digoxin Placebo	57	83	5	82	13	0	25	62	NA	NA	NA	NA
Lewis (1989)	3	87 43	Lisinopril Placebo	NA	NA	0	22	64	14	39	NA	NA	NA	NA	NA
SOLVD (1991)	41	1285 1284	Enalapril Placebo	61	80	11	57	30	2	25	71	10	26	42	NA
SOLVD (1992)	37	2111 2117	Enalapril Placebo	59	89	67	33	0	0	28	83	4	15	37	NA
Colfer (1992)	3	114 58	Benazepril Placebo	62	83	0	54	45	1	25	55	NA	NA	NA	NA
MHFT (1993)	44	83 87	Captopril Placebo	62	75	26	49	24	0	35	69	NA	NA	12	NA
CIBIS (1994)	23	320 321	Bisoprolol Placebo	60	83	0	0	95	5	17	54	13	NA	5	NA
CASSIS (1995)	26	152 48 48	Spirapril Enalapril Placebo	58	83	0	25	56	19	28	70	NA	23	NA	NA
Beller (1995)	3	130 63	Lisinopril Placebo	60	75	0	35	56	9	28	NA	NA	NA	5	NA
Brown (1995)	6	116 125	Fosinopril Placebo	62	80	0	37	54	9	25	NA	NA	NA	NA	NA
Dickstein (1995)	2	108 58	Losartan Enalapril	64	78	0	0	84	16	23	69	NA	NA	23	NA
FEST (1995)	3	155 153	Fosinopril Placebo	63	75	0	65	36	0	26	71	NA	NA	NA	NA
Krum (1995)	3	33 16	Carvedilol Placebo	55	78	0	27	63	10	16	10	NA	NA	NA	NA
Packer (1996)	6	696 398	Carvedilol Placebo	58	77	0	53	44	3	23	NR	NA	NA	NA	NA
PRECISE (1996)	6	133 145	Carvedilol Placebo	60	73	0	40	56	4	22	52	NA	NA	NA	NA
MOCHA (1996)	6	261 84	Carvedilol Placebo	60	76	0	53	60	2	23	52	NA	NA	NA	NA
Colucci (1996)	12	232 134	Carvedilol Placebo	54	85	0	85	14	0	23	41	NA	NA	NA	NA
ANZ HF Coll GROUP (1997)	19	207 208	Carvedilol Placebo	67	80	29	54	16	0	NA	NA	NA	NA	NA	42
Lang (1997)	3	78 38	Losartan Enalapril	58	78	0	47	51	2	25	47	NA	NA	4	NA
Cohn (1997)	6	70 35	Carvedilol Placebo	60	58	0	1	86	13	22	45	NA	NA	NA	NA
The Digitalis Investigation Group (1997)	37	3397 3403	Digoxin Placebo	63	78	13	54	31	2	28	71	NA	28	45	NA
ELITE I (1997)	12	352 370	Losartan Captopril	73	67	0	65	34	2	30	68	23	24	57	NA
Van Veldhuisen (1998)	3	182 62	Imidapril Placebo	61	77	0	77	23	0	34	63	9	NA	NA	NA
CIBIS‐II (1999)	15	1327 1320	Bisoprolol Placebo	61	81	0	0	83	17	28	50	20	NA	NA	NA
STRETCH (1999)	3	633 211	Candesartan Placebo	62	69	0	81	19	0	39	NA	NA	NA	29	NA
Hamroff (1999)	6	16 17	Losartan Placebo	61	49	NA	NA	NA	NA	26	30	NA	39	66	NA
MERIT‐HF Pilot (1999)	6	42 19	Metoprolol Placebo	NR	75	0	56	41	3	27	NA	NA	NA	8	NA
RALES (1999)	24	822 841	Spironolactone Placebo	65	73	0	0	70	29	25	54	NA	NA	NA	NA
Shettigar (1999)	3	102 104	Fosinopril Placebo	62	NA	NA	58	NA	30	NA	NA	NA	NA	NA	NA
RESOLVD (2000)	6	214 212	ACEi/ARB + metoprolol ACEi/ARB + placebo	61	82	7	69	23	1	28	69	NA	25	36	NA
ELITE II (2000)	18	1578 1574	Losartan Captopril	71	70	0	52	43	5	31	79	30	24	49	NA
CELICARD (2000)	12	62 62	Celiprolol Placebo	57	90	0	57	43	1	26	NA	NA	NA	NA	NA
MIC (2000)	6	26 26	Metoprolol Placebo	54	71	0	58	42	0	28	NA	NA	NA	NR	NA
SPICE (2000)	3	179 91	Candesartan Placebo	66	69	0	54	41	6	27	71	23	18	38	NA
Sturm (2000)	13	51 49	Atenolol + enalapril Placebo + enalapril	52	88	7	69	23	1	17	28	16	18	36	NA
REPLACE (2001)	3	301 77	Telmisartan Enalapril	64	89	0	64	36	0	26	NA	NA	NA	NA	46
Val‐HeFT (2001)	23	2511 2499	Valsartan Placebo	63	80	0	62	36	2	27	NA	12	25	7	NA
BEST (2001)	24	1354 1354	Bucindolol Placebo	60	78	0	0	92	8	23	58	11	35	59	NA
Dubach (2002)	12	13 15	Bisoprolol Placebo	58	NA	0	NA	NA	0	26	57	NA	3	43	NA
De Milliano (2002)	6	43 11	Metoprolol Placebo	71	66	0	54	46	0	25	56	9	NA	NA	NA
Cicoira (2002)	12	54 52	Spironolactone Placebo	62	87	NA	NA	NA	NA	33	64	NA	NA	NA	NA
COPERNICUS (2001)	10	1156 1133	Carvedilol Placebo	63	79	0	NA	NA	NA	20	67	NA	NA	NA	65
HEAVEN (2002)	3	70 71	Valsartan Enalapril	67	53	0	70	30	0	NA	43	NA	NA	NA	NA
Mitrovic (2003)	3	174 44	Candesartan Placebo	54	85	0	61	39	0	28	NA	NA	NA	NA	NA
RESOLVD (2003)	11	125 89 86 126	Candesartan + metoprolol or Enalapril + metoprolol Candesartan + enalapril + metoprolol Candesartan + enalapril Candesartan or enalapril	62	82	34	34	16	16	28	66	NA	NA	NA	NA
CHARM‐Added (2003)	41	1276 1272	Candesartan Placebo	64	79	0	24	73	0	28	62	27	30	48	77
CHARM‐Alternative (2003)	33	1013 1015	Candesartan Placebo	66	68	0	47	49	4	30	68	25	27	50	68
SYMPOXYDEX (2004)	6	28 22	Carvedilol Placebo	59	84	0	78	22	0	26	40	NA	NA	NA	NA
CARMEN (2004)	18	191 190 191	Carvedilol + placebo Enalapril + placebo Carvedilol + enalapril	62	81	8	65	27	0	30	67	18	14	31	NA
ENECA (2005)	8	134 126	Nebivolol Placebo	72	73	0	47	49	5	26	NA	26	26	57	NA
CIBIS III (2005)	30	505 505	Bisoprolol Enalapril	72	68	0	49	51	0	29	NA	NA	21	66	NA
Palazzuoli (2005)	12	33 25	Carvedilol Placebo	71	66	0	0	57	43	32	69	NA	NA	NA	NA
Palazzuoli, b (2005)	12	32 27	Carvedilol Placebo	71	64	0	0	58	42	32	69	NA	NA	NA	NA
MERIT‐HF (2009)	12	1990 2001	Metoprolol CR/XL Placebo	64	77	0	41	55	4	28	65	16	24	44	NA
AREA IN‐CHF (2009)	12	231 236	Canrenone Placebo	63	84	0	100	0	0	40	52	8	20	45	47
SHIFT (2010)	23	3241 3264	Ivabradine Placebo	60	66	0	48	50	2	29	68	8	30	66	NA
EMPHASIS‐HF (2011)	21	1364 1373	Eplerenone Placebo	69	78	0	100	0	0	26	69	30	31	67	53
Vizzardi (2014)	44	65 65	Spironolactone Placebo	63	NR	18	82	0	0	36	NA	NA	28	57	NA
PARADIGM‐HF (2014)	27	4187 4212	Sacubitril‐valsartan Enalapril	64	78	5	70	24	1	29	60	37	35	71	63
SOCRATES‐REDUCED (2015)	3	364 92	VERI Placebo	68	80	26	26	24	24	30	53	34	48	78	78
COSMIC‐HF (2016)	5	296 148	OM Placebo	63	83	NA	NA	NA	NA	29	64	19	41	68	29
DEFINE‐HF (2019)	3	131 132	Dapagliflozin Placebo	61	73	0	66	34	0	26	53	40	63	NA	79
DAPA‐HF (2019)	18	2373 2371	Dapagliflozin Placebo	66	77	0	68	31	1	31	56	38	42	NA	48
EMPIRE‐HF (2020)	3	95 95	Empagliflozin Placebo	63	85	6	78	15	0	30	51	37	17	NA	51
VICTORIA (2020)	11	2526 2524	VERI Placebo	67	76	0	59	40	1	29	NA	NA	NA	NA	NA
GALACTIC‐HF (2020)	22	4120 4112	OM Placebo	64	79	0	53	44	3	27	54	27	40	NA	25
EMPEROR‐REDUCED (2020)	16	1863 1867	Empagliflozin Placebo	67	76	0	75	24	1	27	52	37	50	72	31

Abbreviations: ACEi, angiotensin converting enzyme inhibitor; AF, atrial fibrillation; ARB, angiotensin receptor blocker; FUP, followup; HHF, hospitalization for heart failure; LVEF, left ventricular ejection fraction; NA, not available; NYHA, New York Heart Association; OM, omecamtiv mecarbil; RCT, randomized controlled trial; VERI, vericiguat.

The total population consisted of 91,741 patients, predominantly male (mean 76.3%, range 49.0%–90.0%) and with a mean age of 62.7 years (52.0–73.0 years). Most patients were classified under New York Heart Association (NYHA) class II (mean 48.5%) or III (mean 40.5%). Mean baseline LVEF was 27.5% (16.0%–40.0%) and HF etiology was primarily ischemic (mean 58.3%, range 10.0%–83.0%).

Follow‐up lasted from 2.0 [20] to 44.0 [21, 22] months, with the mean being 13.0 months. Total patients/exposure was 150,364 patients/year; 22 RCTs had at least 1000 patients/year.

The number of drug classes forming the background therapy and the proportion of patients taking it at the beginning of the RCTs increased over time (Table S4).

Despite some differences in study quality, the risk of bias was low overall, both globally and in individual domains (Table S5).

### Bayesian NMA results

**Fig. 2:**
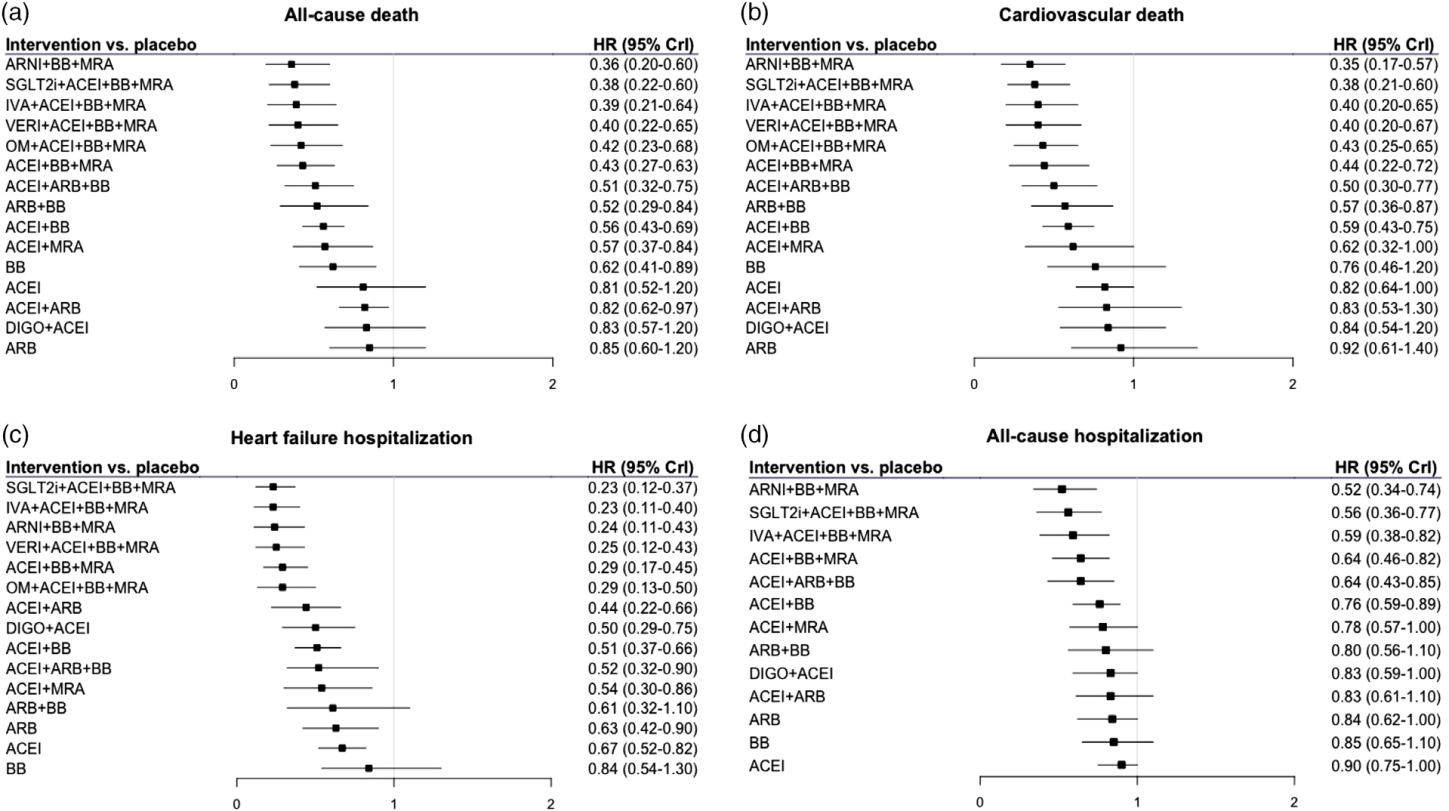
Results of the random‐effects network meta‐analysis for all‐cause death (a), cardiovascular death (b), heart failure hospitalization (c), and all‐cause hospitalization (d). The points and the bars represent the hazard ratios (HRs) and 95% credible intervals (CrI), respectively, for intervention versus placebo (i.e., none of the drugs evaluated in the randomized controlled trials). ACEi, angiotensin converting enzyme inhibitors; ARB, angiotensin receptor blockers; ARNI, angiotensin receptor–neprilysin inhibitor; BB, beta blockers; DIGO, digoxin; IVA, ivabradine; MRA, mineralocorticoid receptor antagonists; OM, omecamtiv mecarbil; SGLT2i, sodium–glucose cotransporter 2 inhibitors; VERI, vericiguat.

The number of events for the endpoints of interest for each RCT are reported in Table S6, whereas the results of the random‐effects NMA are presented in Fig. 2 and Tables S7–S10.

All treatments were associated with some reduction in the risk of the outcomes as compared with none of the drugs evaluated in the RCTs. This effect was invariably significant, with the upper 95% credible interval (CrI) being well below 1 for the combination of BB, ACEi, and MRA, as well as for the combinations of these and other drugs, including omecamtiv mecarbil (OM), ivabradine, vericiguat, and SGLT2i. The magnitude of decrease in the risk of the outcomes was also always among the highest with the combination of ARNI, BB, and MRA (Fig. 2).

As shown in Fig. 2a,b and Tables S7 and S8, ivabradine, vericiguat, or SGLT2i in addition to BB, ACEi, and MRA, or ARNI together with BB and MRA, were associated with the maximum reduction in all‐cause mortality, by 60% to 64% versus placebo, and CV mortality by 60% to 65% versus placebo.

These treatments were also associated with the maximum decrease in the risk of HHF: 77% with either SGLT2i or ivabradine and BB, ACEi, and MRA; 76% with ARNI, BB, and MRA; and 75% with vericiguat, BB, ACEi, and MRA (Fig. 2c and Table S9).

The estimates of risk reduction for all‐cause hospitalization were smaller, indicating a lower efficacy of the interventions, and the CrI were wider because fewer studies reported this endpoint. Complete neurohormonal inhibition with BB, ACEi, and MRA was associated with a 36% decrease in the outcome. The addition of SGLT2i or ivabradine further diminished the risk by 44% and 41%, respectively, and the combination of ARNI with BB and MRA by 48% (Fig. 2d and Table S10).

**Fig. 3:**
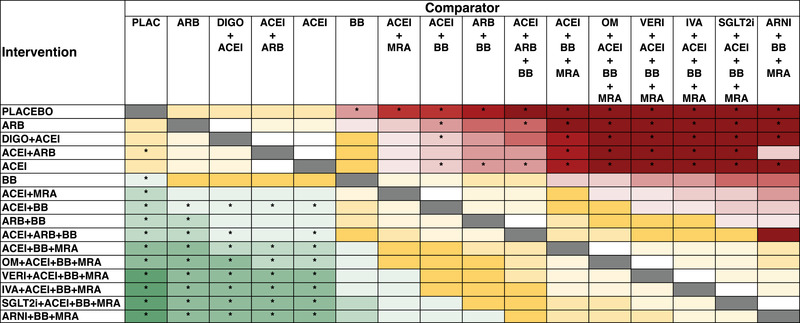
Graphical representation of random‐effects Bayesian network meta‐analysis direct and indirect comparisons for all‐cause mortality. The hazard ratios (HRs) for the comparisons are color coded. Green indicates HR between 0.31 and 0.70, yellow HR between 0.71 and 0.99 (to the left of the grey cells) or between 1.01 and 1.30 (to the right of the grey cells), and red HR higher than 1.31. Within each color, shades become darker with every 0.10‐unit decrease or increase (e.g., HRs between 0.50 and 0.60 are darker green than those between 0.60 and 0.70, and HRs between 1.40 and 1.50 are darker red than those between 1.31 and 1.40). White indicates HR = 1.00, and the symbol “^*^” indicates statistical significance. ACEi, angiotensin converting enzyme inhibitors; ARB, angiotensin receptor blockers; ARNI, angiotensin receptor–neprilysin inhibitor; BB, beta blockers; DIGO, digoxin; IVA, ivabradine; MRA, mineralocorticoid receptor antagonists; OM, omecamtiv mecarbil; SGLT2i, sodium–glucose cotransporter 2 inhibitors; VERI, vericiguat.

**Fig. 4:**
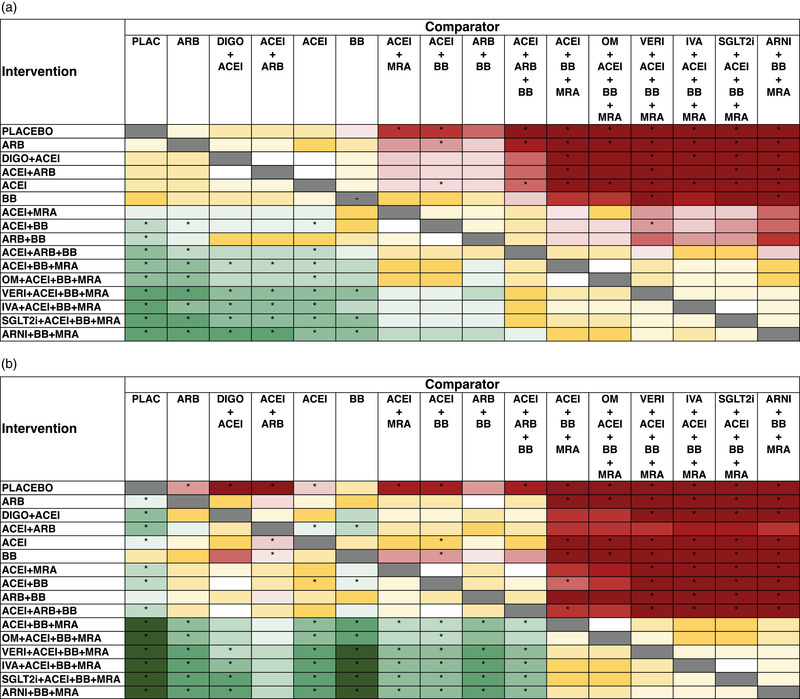
Graphical representation of random‐effects Bayesian network meta‐analysis direct and indirect comparisons for cardiovascular mortality (a) and heart failure hospitalization (b). For color coding, see legend for Fig. 3. ACEi, angiotensin converting enzyme inhibitors; ARB, angiotensin receptor blockers; ARNI, angiotensin receptor–neprilysin inhibitor; BB, beta blockers; DIGO, digoxin; IVA, ivabradine; MRA, mineralocorticoid receptor antagonists; OM, omecamtiv mecarbil; SGLT2i, sodium–glucose cotransporter 2 inhibitors; VERI, vericiguat.

The results of direct and indirect comparisons are visually presented in Figs 3 and 4, while the HR and 95% CrI are reported in Tables S7–10. Overall, neurohormonal inhibition was superior to single‐drug or two‐drug approaches, and combination therapies beyond neurohormonal inhibition (i.e., additional agent besides BB, ACEi, and MRA, or ARNI together with BB and MRA) provided further risk reduction compared with neurohormonal inhibition. Moreover, there was a trend for better outcomes with the schemas including ivabradine, vericiguat, SGLT2i, or ARNI over those including OM.

Network comparisons as well as ranking probabilities were similar in frequentist random‐effects models (Figs S5–S8).

### Sensitivity analysis of different treatment schemas with SGLT2i

**Fig. 5:**
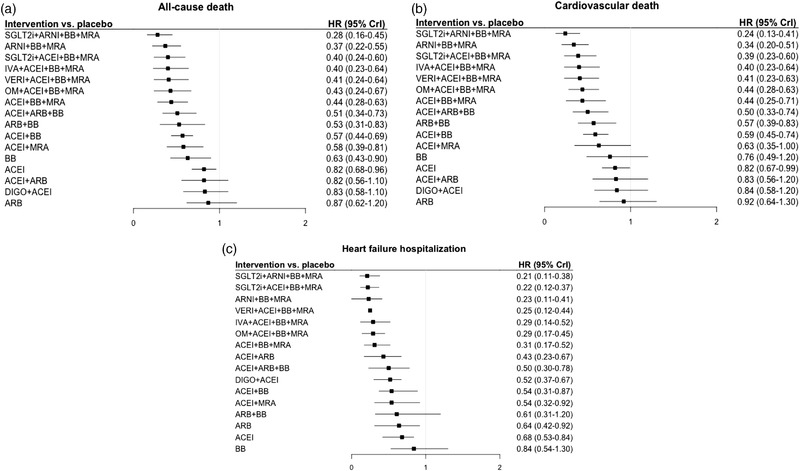
Risk reduction in all‐cause mortality (a), cardiovascular mortality (b), and heart failure hospitalization (c) after distinguishing subgroups of combination therapy with sodium–glucose cotransporter‐2 inhibitors according to concomitant use of angiotensin receptor–neprilysin inhibitor. For each outcome, the hazard ratios (HRs) with 95% credible intervals (CrI)—as calculated by random‐effects network meta‐analysis—are shown for interventions versus placebo (i.e., none of the drugs evaluated in the randomized controlled trials). ACEi, angiotensin converting enzyme inhibitors; ARB, angiotensin receptor blockers; ARNI, angiotensin receptor–neprilysin inhibitor; BB, beta blockers; DIGO, digoxin; IVA, ivabradine; MRA, mineralocorticoid receptor antagonists; OM, omecamtiv mecarbil; SGLT2i, sodium–glucose cotransporter 2 inhibitors; VERI, vericiguat.

In DAPA‐HF and EMPEROR‐REDUCED, 508 (10.7%) and 727 (19.5%) patients, respectively, were taking ARNI at baseline. When the SGLT2i node was split in two according to the use of ARNI, the schema including SGLT2i, ARNI, BB, and MRA was superior to the one with SGLT2i, BB, ACEi, and MRA in decreasing all‐cause and CV mortality. In fact, it was associated with the greatest risk reduction for these outcomes, by 72% and 76%, respectively (Fig. 5 and Fig. S9). Conversely, either drug combination similarly diminished the risk of HHF (Fig. 5 and Fig. S9).

### Treatment ranking

The highest SUCRA values for all‐cause death were obtained for treatment with ARNI, BB, and MRA (87.7%); SGLT2i, BB, ACEi, and MRA (85.0%); ivabradine, BB, ACEi, and MRA (80.5%); and vericiguat, BB, ACEi, and MRA (78.1%) (Fig. S10).

These drug combinations also ranked the highest for CV death and HHF—the SUCRA values were 91.7% for CV death and 85.1% for HHF with ARNI, BB, and MRA; 80.4% and 83.1% with vericiguat, BB, ACEi, and MRA; 85.8% and 90.4% with SGLT2i, BB, ACEi, and MRA; and 80.4% and 89.0% with ivabradine, BB, ACEi, and MRA (Figs S11 and S12).

Finally, SUCRA scores for all‐cause hospitalization were 93.6% for ARNI, BB, and MRA; 88.2% for SGLT2i, BB, ACEi, and MRA; 82.7% for ivabradine, BB, ACEi, and MRA; and 74.2% for ACEi, ARB, and BB (Fig. S13).

The results of the leave‐one‐out analysis were also comparable overall to the main ones (Table S11).

### Heterogeneity, publication bias, convergence, and node‐split analysis

Global I^2^ for the endpoint of all‐cause death was 16% (95% CrI: 0.4%–40.1%), showing low heterogeneity. τ^2^ was also very low (0.0010).

Comparison‐adjusted funnel plots and Egger's regressions were not suggestive of significant publication bias (Fig. S14).

Gelman–Rubin–Brooks plot for all‐cause death showed high convergence (Fig. S15).

Node‐split analysis did not show significant inconsistency between direct and indirect evidence for all endpoints, except for some inconsistency in the HHF model (Figs S16–S19).

### Metaregression analyses

The covariates with less than 30% of missing values were age (missing: 4.3%), sex (5.7%), baseline NYHA class III/IV (8.6%), baseline LVEF (7.1%), and ischemic etiology of HF (27.1%). Accounting for these variables and for the risk of time bias yielded DIC values similar to those of the reference models, with the changes in the goodness of fit being negligible (Table S12).

## Discussion

This NMA provides a comprehensive synthesis of phase 2 and 3 RCT data on HFrEF pharmacotherapy, encompassing more than 90,000 patients recruited over more than 30 years.

This ample body of evidence extends the conclusions of previous NMA [3, 4], confirming that the step‐wise addition of new drugs to the pre‐existing medical therapy has progressively and substantially ameliorated the prognosis of patients with HFrEF, up to abating the risk of mortality and HHF by around 65% and 75%, respectively, as compared with no treatment.

It is also shown that the greatest benefit has been attained by combining neurohormonal inhibitors and newer molecules, as well as by substituting ARNI for ACEi in a treatment schema that also includes BB and MRA. Furthermore, a sensitivity analysis—taking into account the minority of participants in DAPA‐HF and EMPEROR‐REDUCED who were on ARNI at baseline—indicates that the use of SGLT2i, ARNI, BB, and MRA is associated with the maximum improvement in outcomes, with all‐cause and CV death both being decreased by more than 70%.

These findings buttress the paradigm that polytherapy with neurohormonal inhibitors and the most recent medications should be started simultaneously rather than sequentially in subjects with HFrEF [23].

The most striking impact of contemporary pharmacotherapy on the course of HFrEF is the decline in all‐cause mortality, which is nowadays more than halved as compared with no treatment. This effect is even more notable considering that HFrEF drugs mainly prevent CV deaths, and thereby the competing risk of non‐CV death has grown over time [24, 25].

As expected, we observed the greatest relative risk reduction for HHF, the hard endpoint that most immediately reflects the advantages afforded by an intervention for HF [26, 27, 28, 29].

When possible, we also assessed the effect of HFrEF medications on all‐cause hospitalization. This information was not available in 63% of the RCTs examined, which is a reason for concern since admission for non‐CV conditions is part of the clinical events directing the trajectory and influencing the prognosis of HFrEF [28, 30, 31, 32]. With this limit recognized, we found that ivabradine or SGLT2i in addition to neurohormonal inhibitors, and ARNI together with BB and MRA, decreased the risk of all‐cause hospitalization more than mere neurohormonal inhibition. These results are consistent with the main ones and, again, indicate that HFrEF pharmacotherapy should be expanded beyond neurohormonal inhibitors.

Although the statistical significance of RCT results is fundamental, other considerations may motivate prioritization of HFrEF medications. Unlike ACEi, ARB have never been evaluated in an RCT together with BB and MRA. The cohorts of the RCTs investigating ARNI and SGLT2i were bigger than those of the RCTs with ivabradine and vericiguat. Furthermore, ivabradine and vericiguat were restricted to selected patients, that is, those having a sinus rhythm with the resting heart rate being at least 70 beats per minute or an episode of worsening HF within 6 months, respectively. Finally, vericiguat has not been introduced in clinical practice yet. Therefore, at present, SGLT2i, ARNI (or ACEi), BB, and MRA are viewed as the pillars of HFrEF medical therapy, while ivabradine and vericiguat represent second‐line options for HFrEF with persisting symptoms [33].

Interestingly, we observed that SGLT2i, ARNI, BB, and MRA had higher efficacy than SGLT2i, BB, ACEi, and MRA, and conferred the highest protection against total and CV death, even though this was in a sensitivity analysis. This is in agreement with a prior cross‐trial analysis [31] and further supports the emphasis on early prescription of both SGLT2i and ARNI to HFrEF patients [1].

In the RCTs we analyzed, the percentage of subjects taking neurohormonal inhibitors at the time of randomization was variable. Moreover, these drugs were most often, but not always, titrated to the target dose, as established in previous RCTs. For instance, in DAPA‐HF, the vast majority of subjects, but not all, were on BB and 71% were on MRA at baseline, with the mean dose of spironolactone and eplerenone being 31.4 and 32.5 mg against the recommended dose of 50 mg [5].

Our NMA shows that, in aggregate, the combination of neurohormonal inhibitors and more recent compounds was superior to neurohormonal inhibitors alone, but does not discriminate between subgroups with different patterns of background neurohormonal inhibition (e.g., BB, but no MRA). Patient‐level data would be needed to achieve this scope, a requisite that clearly cannot be met when examining a total of 69 RCTs. Nonetheless, earlier studies suggest that target doses of neurohormonal inhibitors are only modestly more effective than lower ones [34, 35, 36, 37, 38].

Along these lines, patient features were diverse across the RCTs, primarily because the enrollment criteria changed. We did acknowledge these dissimilarities and carried out several metaregression analyses, but we could not thoroughly investigate whether HFrEF drugs performed differently in specific subsets, such as in case of female sex or higher NYHA class.

However, we argue that the approach of the present work corresponds to that of the guidelines, which condense the RCT evidence and delineate principles of therapy, based on the overall characteristics and results of the studies, to be applied to the wide HFrEF population. The clinician is then expected to evaluate the patient and tailor the treatment [33, 39, 40]. With a spectrum of medications being available to improve outcomes, factors such as heart rhythm, blood pressure, renal function, and diabetes can be individually targeted [40]. In light of the complexity of HFrEF syndrome and the great degree of heterogeneity of patient profiles in the real‐life setting, the possibility of choosing among several, effective therapeutic schemas is invaluable.

Besides not distinguishing between patient subgroups, our work has other limitations.

First, it covers a uniquely high number of HFrEF RCTs, but others were not included because the search criteria were not met. Second, some RCTs tested drugs that are not indicated for HF patients, such as atenolol and canrenone. Third, the NMA relies on the assumption that all drugs belonging to the same class have similar efficacy, which may not be true. Fourth, we omitted nonpharmacological interventions, such as in particular implantable‐cardioverter defibrillators and cardiac resynchronization therapy, since published data are not granular enough to discriminate treatment strategies purely based on medications from those including both drugs and devices.

## Conclusions

The sequence of RCTs of medical therapy for HFrEF corresponds to a gradual but constant improvement in major outcomes. After the successful adoption of BB, ACEi, and MRA, the addition of new drugs to neurohormonal inhibitors has further diminished the risk of death, HHF, and—to a certain extent—all‐cause hospitalization. According to this evidence, combination pharmacotherapy beyond neurohormonal inhibition—particularly with SGLT2i and ARNI—must be pursued in patients with HFrEF.

## Conflict of interest

Vincenzo De Marzo received speaker fees from Astra Zeneca, Daiichi‐Sankyo, Bristol‐Myers Squibb, and Bayer, all outside of the scope of the submitted work. Gianluigi Savarese reports grants and personal fees from Vifor and AstraZeneca; grants and nonfinancial support from Boehringer Ingelheim; personal fees from Societa´ Prodotti Antibiotici, Roche, Servier, GENESIS, Cytokinetics, Medtronic; and grants from Novartis, Boston Scientific, PHARMACOSMOS, Merck, and Bayer, all outside of the scope of the submitted work. Massimo Iacoviello received speaker and/or advisor fees from Novartis, AstraZeneca, Boehringer–Ingelheim, and MSD, all outside of the scope of the submitted work. Italo Porto received speaker and/or advisor fees from Biotronik, ABIOMED, Terumo, Philips, Sanofi, Amgen, Daiichi‐Sankyo, and Bayer, all outside of the scope of the submitted work. Pietro Ameri received speaker and/or advisor fees from AstraZeneca, Novartis, Bayer, Daiichi Sankyo, MSD, Janssen, GlaxoSmithKline, and Amgen, all outside of the scope of the submitted work, and served as a scientific consultant on behalf of the Department of Internal Medicine of the University of Genova for Bayer and Daiichi Sankyo, outside of the scope of the submitted work. The other authors have no conflict of interest to disclose related to the contents of this article.

## Supporting information


**Figure S1**: Preferred Reporting Items for Systematic Reviews and Meta‐Analysis (PRISMA) flowchart of study selection.
**Figure S2**: Network of the comparisons between different pharmacological treatments for the secondary endpoint of cardiovascular death.
**Figure S3**: Network of the comparisons between different pharmacological treatments for the secondary endpoint of hospitalization for heart failure.
**Figure S4**: Network of the comparisons between different pharmacological treatments for the secondary endpoint of all‐cause hospitalization.
**Figure S5**: Results of random‐effects frequentist network meta‐analysis for all‐cause death.
**Figure S6**: Results of random‐effects frequentist network meta‐analysis for cardiovascular death.
**Figure S7**: Results of random‐effects frequentist network meta‐analysis for heart failure hospitalization.
**Figure S8**: Results of random‐effects frequentist network meta‐analysis for all‐cause hospitalization.
**Figure S9**: Risk reduction in all‐cause mortality (A), cardiovascular mortality (B), and heart failure hospitalization (C), as calculated by frequentist random‐effects network meta‐analysis, after distinguishing subgroups of combination therapy with sodium‐glucose co‐transporter‐2 inhibitors according to concomitant use of angiotensin receptor‐neprilysin inhibitor.
**Figure S10**: Surface under the cumulative ranking area (SUCRA) scores for all‐cause death.
**Figure S11**: Surface under the cumulative ranking area (SUCRA) scores for cardiovascular death.
**Figure S12**: Surface under the cumulative ranking area (SUCRA) scores for heart failure hospitalization.
**Figure S13**: Surface under the cumulative ranking area (SUCRA) scores for all‐cause hospitalization.
**Figure S14**: Comparison‐adjusted funnel plots for the primary endpoint of all‐cause death.
**Figure S15**: Gelman and Rubin plots to evaluate convergence for all‐cause death.
**Figure S16**: Node‐split analyses for the primary endpoint of all‐cause death.
**Figure S17**: Node‐split analyses for cardiovascular mortality.
**Figure S18**: Node‐split analyses for heart failure hospitalization.
**Figure S19**: Node‐split analyses for all‐cause hospitalization.
**Table S1**: Preferred Reporting Items for Systematic Reviews and Meta‐Analysis (PRISMA) checklist.
**Table S2**: Full electronic search in databases through November 30, 2020.
**Table S3**: List of references included in the network meta‐analysis.
**Table S4**: Reported concomitant medical and device therapy for the selected randomized controlled trials.
**Table S5**: Risk of bias assessment in the studies included in the network meta‐analysis and quality of evidence for the pairwise comparisons.
**Table S6**: Number of events for the endpoints of interest in the included studies.
**Table S7**: Results of random‐effects Bayesian network meta‐analysis for the primary endpoint of all‐cause death.
**Table S8**: Results of random‐effects Bayesian network meta‐analysis for the secondary endpoint of cardiovascular death.
**Table S9**: Results of random‐effects Bayesian network meta‐analysis for the secondary endpoint of hospitalization for heart failure.
**Table S10**: Results of random‐effects Bayesian network meta‐analysis for the secondary endpoint of all‐cause hospitalization.
**Table S11**: Surface under the cumulative ranking area (SUCRA) scores of random‐effects network meta‐analysis for the endpoint of all‐cause death on a scale from 0 to 100 with the leave‐one‐study out approach.
**Table S12**: Results for the meta‐regression analyses for all‐cause death, cardiovascular death, heart failure hospitalization, and all‐cause hospitalization.
