# Supplementary material for: Network meta‐analysis of medical therapy efficacy in more than 90,000 patients with heart failure and reduced ejection fraction
Source: J Intern Med. 2022 Apr 12;292(2):333–49. doi: 10.1111/joim.13487 (PMC9546056; doi:10.1111/joim.13487)
Supplement: Supplementary file 1 — Figure S1: Preferred Reporting Items for Systematic Reviews and Meta‐Analysis (PRISMA) flowchart of study selection. Figure S2: Network of the comparisons between different pharmacological treatments for the secondary endpoint of cardiovascular death. Figure S3: Network of the comparisons between different pharmacological treatments for the secondary endpoint of hospitalization for heart failure. Figure S4: Network of the comparisons between different pharmacological treatments for the secondary endpoint of all‐cause hospitalization. Figure S5: Results of random‐effects frequentist network meta‐analysis for all‐cause death. Figure S6: Results of random‐effects frequentist network meta‐analysis for cardiovascular death. Figure S7: Results of random‐effects frequentist network meta‐analysis for heart failure hospitalization. Figure S8: Results of random‐effects frequentist network meta‐analysis for all‐cause hospitalization. Figure S9: Risk reduction in all‐cause mortality (A), cardiovascular mortality (B), and heart failure hospitalization (C), as calculated by frequentist random‐effects network meta‐analysis, after distinguishing subgroups of combination therapy with sodium‐glucose co‐transporter‐2 inhibitors according to concomitant use of angiotensin receptor‐neprilysin inhibitor. Figure S10: Surface under the cumulative ranking area (SUCRA) scores for all‐cause death. Figure S11: Surface under the cumulative ranking area (SUCRA) scores for cardiovascular death. Figure S12: Surface under the cumulative ranking area (SUCRA) scores for heart failure hospitalization. Figure S13: Surface under the cumulative ranking area (SUCRA) scores for all‐cause hospitalization. Figure S14: Comparison‐adjusted funnel plots for the primary endpoint of all‐cause death. Figure S15: Gelman and Rubin plots to evaluate convergence for all‐cause death. Figure S16: Node‐split analyses for the primary endpoint of all‐cause death. Figure S17: Node‐split analyses for cardiovascular mortality [file JOIM-292-333-s001.docx]

**Network meta-analysis of medical therapy effects in more than 90,000 patients**

**with heart failure and reduced ejection fraction**

Vincenzo De Marzo, Gianluigi Savarese, Lucia Tricarico, Sofia Hassan, Massimo Iacoviello, Italo Porto, Pietro Ameri

**SUPPLEMENTARY MATERIAL**

**Full Methods**

This NMA was registered in the PROSPERO database (ID: CRD42021228040) and performed according to the Preferred Reporting Items for Systematic Reviews and Meta-Analysis (PRISMA) recommendations (**Table S1**) [1]. All data are available within the article and Supplementary Material.

**Search strategy**

We systematically searched the MEDLINE, Embase, Scopus, and Cochrane Library databases for RCTs in HFrEF using the search strings “heart failure” and/or “randomized controlled trial”. We also thoroughly screened the references of original research articles, guidelines, reviews, and meta-analyses to identify additional eligible studies.

The search, which is outlined in **Table S2**, was limited to English language, peer-reviewed publications and is updated to November 30^th^, 2020.

**Eligibility criteria and data extraction**

After identifying the phase 2 and 3 RCTs enrolling individuals with chronic HF and left ventricular ejection fraction (LVEF) less than 45%, we excluded those with greater than 10% of subjects with LVEF at least 45%, which were not representative of the broad HFrEF population (i.e. investigating only specific subsets of patients), or for which there was no published information about the rates of all-cause death in both the intervention and placebo or comparator arms. We also excluded the studies comparing different molecules of the same drug class (e.g. BB vs. BB).

Three investigators (VDM, LT, and SH) independently reviewed the retrieved articles and extracted baseline patient characteristics and therapies, follow-up duration, total numbers of patients and outcome events in the arms of each RCT, and measures of relative risk (hazard ratios, HR), if available. Disagreements were solved by involvement of another two investigators (PA and MI).

To account for concomitant treatments, the tested compound was considered as combined with other drugs if more than 50% of the patients took these medications [2-4].

The collected information was entered into a database (double entry with reconciliation).

**Analysis outcomes**

The primary outcome was all-cause death. The secondary outcomes of CV death, HHF, and all-cause hospitalization were also investigated for those RCTs with data available.

**Assessment of risk of bias**

We used the Cochrane Collaboration’s tool to assess risk of bias in six pre-specified domains: selection bias, performance bias, detection bias, attrition bias, reporting bias, and other bias [5]. Studies were defined as at low risk of bias if all six domains were classified as low risk, at medium risk of bias if at least one domain prompted some concern, and at high risk of bias if the probability of bias in at least one domain was deemed high or if concerns were raised for several domains.

Comparison-adjusted funnel plots and Egger’s regressions were employed to visualize publication bias, as previously described [6].

**Quality of evidence**

We examined the quality of evidence (high, low, or unclear) according to the Grading of Recommendations, Assessment, Development, and Evaluation (GRADE) tool [7]. The following parameters were taken into consideration: study design, risk of bias, inconsistency, indirect evidence, imprecision, and publication bias.

**Network meta-analysis**

The NMA was graphically represented by means of a network, in which each treatment (either a single drug class or a combination therapy, as defined above) was connected to every other treatment, irrespective of the existence of head-to-head comparisons [8-10]. The thickness of the connecting lines was proportional to the number of patients/years.

Interventions with different molecules belonging to the same pharmacological class, e.g. BB, were pooled together to form one node.

As previously done [2-4, 11], the NMA comprised both a fixed-effects model and a more conservative random-effects model within a Bayesian framework using R software and JAGS software [12]. The Markov chain Monte Carlo method was used running two chains with 200,000 iterations after a burn-in of 100,000. Non-informative priors were used. Results of the random-effects model are presented unless the fixed-effects model resulted in a more parsimonious model.

The dataset for the models included the reported number of patients with an event per arm at the end of the RCT, the total number of patients randomized per arm, and the mean or median follow-up duration of the RCT (arm-based data). Thereafter, the log mean/median follow-up time was used to transform the probability of an event into a constant rate for RCT trial arm by assuming an underlying Poisson process, and a log link was used to model the event rates.

The outputs of the models are presented as HRs for each treatment versus placebo with 95% credible intervals (CrI) for the Bayesian probability that the treatment is better than placebo. Results were also computed for all pairwise comparisons.

Heterogeneity was measured through the I^2^ statistic, with less than 25%, 25-50% and greater than 50% I^2^ indicating low, moderate, and high heterogeneity, respectively, as well as through τ^2^ heterogeneity [13]. Convergence was evaluated according to Gelman-Rubin-Brooks [14].

Consistency was assessed comparing direct and indirect evidence with the node-splitting technique.

The probability that a treatment ranked among the most effective for the outcomes of interest was calculated as a Surface Under the Cumulative Ranking Area (SUCRA) value between 0% and 100%. The higher the SUCRA value, the higher the likelihood that a therapy ranks high; the closer to 0 the SUCRA value, the more likely that a therapy is in the bottom rank [15].

For the primary endpoint, sensitivity analyses were also conducted by using a frequentist random-effects approach with the DerSimonian-Laird estimator [16], and by excluding the selected studies sequentially (leave-one-out analysis).

Since SGLT2i, ARNI, BB, and MRA are now proposed as first-line treatment for HFrEF [17], we also ran the random-effects Bayesian NMA and the frequentist meta-analysis after considering separately SGLT2i + neurohormonal inhibitors and SGLT2i + ARNI + BB + MRA, although in DAPA-HF and EMPEROR-REDUCED the proportion of participants on ARNI was less than 50%, thus not meeting the threshold to define ARNI as a component of a combination therapy with SGLT2i (see Eligibility criteria and data extraction).

Meta-regression analyses were carried out to determine the impact of several covariates with less than 30% missing values on the outcomes—namely, age (missing: 4.3%), sex (missing: 5.7%), baseline NYHA class III/IV (missing: 8.6%), baseline left ventricular ejection fraction (LVEF, missing: 7.1%), and ischemic etiology of HF (missing: 27.1%). The models were re-run excluding these covariates, and the goodness of fit of each regression model was compared with that of the original model by means of the Deviance Information Criterion (DIC), with a five-unit DIC reduction being considered suggestive for a goodness-of-fit improvement [18].

Finally, we accounted for the fact that the selected RCTs span 33 years by dividing the time between the oldest and the latest RCTs in centiles and assigning a centile to each RCT based on the year of publication: The risk of time bias was then graded from 0% to 100%, where 0% corresponds to the most recent RCT (lowest risk of time bias) and 100% to the oldest one (highest risk of time bias).

The NMA was conducted in the R environment (RStudio Desktop, version 1.2.5033) with *forestplot*, *gemtc*, *ggplot2*, and *netmeta* packages. We set statistical significance at p less than 0.05 for the frequentist NMA.

**References**

1 Hutton B, Salanti G, Caldwell DM*, et al.* The PRISMA extension statement for reporting of systematic reviews incorporating network meta-analyses of health care interventions: checklist and explanations. *Ann Intern Med* 2015; **162:** 777-84.

2 Ameri P, De Marzo V, Zoccai GB*, et al.* Efficacy of new medical therapies in patients with heart failure, reduced ejection fraction and chronic kidney disease already receiving neurohormonal inhibitors: a network meta-analysis. *Eur Heart J Cardiovasc Pharmacother* 2021.

3 Burnett H, Earley A, Voors AA, Senni M, McMurray JJ, Deschaseaux C, Cope S. Thirty Years of Evidence on the Efficacy of Drug Treatments for Chronic Heart Failure With Reduced Ejection Fraction: A Network Meta-Analysis. *Circ Heart Fail* 2017; **10**.

4 Komajda M, Bohm M, Borer JS, Ford I, Tavazzi L, Pannaux M, Swedberg K. Incremental benefit of drug therapies for chronic heart failure with reduced ejection fraction: a network meta-analysis. *Eur J Heart Fail* 2018; **20:** 1315-22.

5 Sterne JAC, Savovic J, Page MJ*, et al.* RoB 2: a revised tool for assessing risk of bias in randomised trials. *BMJ* 2019; **366:** l4898.

6 Salanti G, Del Giovane C, Chaimani A, Caldwell DM, Higgins JP. Evaluating the quality of evidence from a network meta-analysis. *PLoS One* 2014; **9:** e99682.

7 Guyatt GH, Oxman AD, Schunemann HJ, Tugwell P, Knottnerus A. GRADE guidelines: a new series of articles in the Journal of Clinical Epidemiology. *J Clin Epidemiol* 2011; **64:** 380-2.

8 Caldwell DM, Ades AE, Higgins JP. Simultaneous comparison of multiple treatments: combining direct and indirect evidence. *BMJ* 2005; **331:** 897-900.

9 Jansen JP, Crawford B, Bergman G, Stam W. Bayesian meta-analysis of multiple treatment comparisons: an introduction to mixed treatment comparisons. *Value Health* 2008; **11:** 956-64.

10 Jansen JP, Fleurence R, Devine B*, et al.* Interpreting indirect treatment comparisons and network meta-analysis for health-care decision making: report of the ISPOR Task Force on Indirect Treatment Comparisons Good Research Practices: part 1. *Value Health* 2011; **14:** 417-28.

11 Dias S, Sutton AJ, Ades AE, Welton NJ. Evidence synthesis for decision making 2: a generalized linear modeling framework for pairwise and network meta-analysis of randomized controlled trials. *Med Decis Making* 2013; **33:** 607-17.

12 Plummer M. JAGS: A Program for Analysis of Bayesian Graphical Models using Gibbs Sampling. *3rd International Workshop on Distributed Statistical Computing (DSC 2003); Vienna, Austria* 2003; **124**.

13 Mills EJ, Thorlund K, Ioannidis JP. Demystifying trial networks and network meta-analysis. *BMJ* 2013; **346:** f2914.

14 Gelman A, Rubin DB. Markov chain Monte Carlo methods in biostatistics. *Stat Methods Med Res* 1996; **5:** 339-55.

15 Spineli LM. An empirical comparison of Bayesian modelling strategies for missing binary outcome data in network meta-analysis. *BMC Med Res Methodol* 2019; **19:** 86.

16 Jackson D, White IR, Riley RD. A matrix-based method of moments for fitting the multivariate random effects model for meta-analysis and meta-regression. *Biom J* 2013; **55:** 231-45.

17 McDonagh TA, Metra M, Adamo M*, et al.* 2021 ESC Guidelines for the diagnosis and treatment of acute and chronic heart failure. *Eur Heart J* 2021; **42:** 3599-726.

18 Spiegelhalter DJ, Abrams KR, Myles JP. *Bayesian approaches to clinical trials and health-care evaluation*. Chichester: John Wiley & Sons. 2004.

**Supplementary tables**

**Table S1. Preferred Reporting Items for Systematic Reviews and Meta-Analysis** (**PRISMA) checklist.**

| **Section/topic** | **#** | **Checklist item** | **Reported on page #** |
| --- | --- | --- | --- |
| **TITLE** | | |  |
| Title | 1 | Identify the report as a systematic review incorporating a network meta-analysis (or related form of meta-analysis). | 1 |
| **ABSTRACT** | | |  |
| Structured summary | 2 | Provide a structured summary including, as applicable: 1) Background: main objectives; 2) Methods: data sources; study eligibility criteria, participants, and interventions; study appraisal; and synthesis methods, such as network meta-analysis; 3) Results: number of studies and participants identified; summary estimates with corresponding  confidence/credible intervals; treatment rankings may also be discussed. Authors may choose to summarize pairwise comparisons against a chosen treatment included in their analyses for brevity. 4) Discussion/Conclusions: limitations; conclusions and implications of findings. 5) Other: primary source of funding; systematic review registration number with registry name. | 2 |
| **INTRODUCTION** | | |  |
| Rationale | 3 | Describe the rationale for the review in the context of what is already known, including mention of why a network meta-analysis has been conducted. | 3 |
| Objectives | 4 | Provide an explicit statement of questions being addressed with reference to participants, interventions, comparisons, outcomes, and study design (PICOS). | 3 |
| **METHODS** | | |  |
| Protocol and registration | 5 | Indicate if a review protocol exists, if and where it can be accessed (e.g., Web address), and, if available, provide registration information including registration number. | 3 |
| Eligibility criteria | 6 | Specify study characteristics (e.g., PICOS, length of follow-up) and report characteristics (e.g., years considered, language, publication status) used as criteria for eligibility, giving rationale. Clearly describe eligible treatments included in the treatment network, and note whether any have been clustered or merged into the same node (with justification). | 4-5 |
| Information sources | 7 | Describe all information sources (e.g., databases with dates of coverage, contact with study authors to identify additional studies) in the search and date last searched. | 4 |
| Search | 8 | Present full electronic search strategy for at least one database, including any limits used, such that it could be repeated. | 4 |
| Study selection | 9 | State the process for selecting studies (i.e., screening, eligibility, included in systematic review, and, if applicable, included in the meta-analysis). | 4 |
| Data collection process | 10 | Describe method of data extraction from reports (e.g., piloted forms, independently, in duplicate) and any processes for obtaining and confirming data from investigators. | 4-5 |
| Data items | 11 | List and define all variables for which data were sought (e.g., PICOS, funding sources) and any assumptions and simplifications made. | 4-5 |
| Geometry of the network | S1 | Describe methods used to explore the geometry of the treatment network under study and potential biases related to it. This should include how the evidence base has been graphically summarized for presentation, and what characteristics were compiled and used to describe the evidence base to readers. | 4-5 |
| Risk of bias within individual studies | 12 | Describe methods used for assessing risk of bias of individual studies (including specification of whether this was done at the study or outcome level), and how this information is to be used in any data synthesis. | 4-5 |
| Summary measures | 13 | State the principal summary measures (e.g., risk ratio, difference in means). Also describe the use of additional summary measures assessed, such as treatment rankings and surface under the cumulative ranking curve (SUCRA) values, as well as modified approaches used to present summary findings from meta-analyses. | 4-5 |
| Planned method of analysis | 14 | Describe the methods of handling data and combining results of studies for each network meta-analysis. This should include, but not be limited to: handling of multi-group trials; Selection of variance structure; Selection of prior distributions in Bayesian analyses; and Assessment of model fit. | 5-6 |
| Assessment of inconsistency | S2 | Describe the statistical methods used to evaluate the agreement of direct and indirect evidence in the treatment network(s) studied. Describe efforts taken to address its presence when found. | 5-6 |
| Risk of bias across studies | 15 | Specify any assessment of risk of bias that may affect the cumulative evidence (e.g., publication bias, selective reporting within studies). | 4-5 |
| Additional analyses | 16 | Describe methods of additional analyses if done, indicating which were pre-specified. This may include, but not be limited to, the following: sensitivity or subgroup analyses; Meta-regression analyses; Alternative formulations of the treatment network; and Use of alternative prior distributions for Bayesian analyses (if applicable). | 5-6 |
| **RESULTS** | | |  |
| Study selection | 17 | Give numbers of studies screened, assessed for eligibility, and included in the review, with reasons for exclusions at each stage, ideally with a flow diagram. | 6 |
| Presentation of network structure | S3 | Provide a network graph of the included studies to enable visualization of the geometry of the treatment network. | 6-7 |
| Summary of network geometry | S4 | Provide a brief overview of characteristics of the treatment network. This may include commentary on the abundance of trials and randomized patients for the different interventions and pairwise comparisons in the network, gaps of evidence in the treatment network, and potential biases reflected by the network structure. | 6-7 |
| Study characteristics | 18 | For each study, present characteristics for which data were extracted (e.g., study size, PICOS, follow-up period) and provide the citations. | 6-7 |
| Risk of bias within studies | 19 | Present data on risk of bias of each study and, if available, any outcome level assessment. | 9 |
| Results of individual studies | 20 | For all outcomes considered (benefits or harms), present, for each study: 1) simple summary data for each intervention group, and 2) effect estimates and confidence intervals. Modified approaches may be needed to deal with information from larger networks. | 7-8 |
| Synthesis of results | 21 | Present results of each meta-analysis done, including confidence/credible intervals. In larger networks, authors may focus on comparisons versus a particular comparator (e.g., placebo or standard care), with full findings presented in an appendix. League tables and forest plots may be considered to summarize pairwise comparisons. If additional summary measures were explored (such as treatment rankings), these should also be presented. | 7-8-9 |
| Exploration of inconsistency | S5 | Describe results from investigations of inconsistency. This may include such information as measures of model fit to compare consistency and inconsistency models, P values from statistical tests, or summary of inconsistency estimates from different parts of the treatment network. | 9 |
| Risk of bias across studies | 22 | Present results of any assessment of risk of bias across studies for the evidence base being studied. | 8 |
| Results of additional analyses | 23 | Give results of additional analyses, if done (e.g., sensitivity or subgroup analyses, meta-regression analyses, alternative network geometries studied, alternative choice of prior distributions for Bayesian analyses, and so forth). | 9 |
| **DISCUSSION** | | |  |
| Summary of evidence | 24 | Summarize the main findings, including the strength of evidence for each main outcome; consider their relevance to key groups (e.g., health care providers, researchers, and policymakers). | 9-10-11-12 |
| Limitations | 25 | Discuss limitations at study and outcome level (e.g., risk of bias), and at review level (e.g., incomplete retrieval of identified research, reporting bias). Comment on the validity of the assumptions, such as transitivity and consistency. Comment on any concerns regarding network geometry (e.g., avoidance of certain comparisons). | 12 |
| Conclusions | 26 | Provide a general interpretation of the results in the context of other evidence, and implications for future research. | 12-13 |
| **FUNDING** | | |  |
| Funding | 27 | Describe sources of funding for the systematic review and other support (e.g., supply of data); role of funders for the systematic review. This should also include information regarding whether funding has been received from manufacturers of treatments in the network and/or whether some of the authors are content experts with professional conflicts of interest that could affect use of treatments in the network. | 1 |

**Table S2. Full electronic search in databases through November 30, 2020.**

| **Query** | | **Items found** |
| --- | --- | --- |
| 1 | Heart failure | 299662 |
| 2 | Randomized controlled trial | 685341 |
| 3 | Heart failure AND random* | 30239 |
| 4 | Heart failure AND randomized controlled trial | 19679 |

**Table S3. List of references included in the network meta-analysis.**

1. Chalmers JP, West MJ, Cyran J, De La Torre D, Englert M, Kramar M, Lewis GR, Maranhao MF, Myburgh DP, Schuster P, et al. Placebo-controlled study of lisinopril in congestive heart failure: a multicentre study. J Cardiovasc Pharmacol 1987;9 Suppl 3:S89-97.

2. Group CTS. Effects of enalapril on mortality in severe congestive heart failure. Results of the Cooperative North Scandinavian Enalapril Survival Study (CONSENSUS). N Engl J Med 1987;316(23):1429-35.

3. Comparative effects of therapy with captopril and digoxin in patients with mild to moderate heart failure. The Captopril-Digoxin Multicenter Research Group. JAMA 1988;259(4):539-44.

4. Lewis GR. Comparison of lisinopril versus placebo for congestive heart failure. Am J Cardiol 1989;63(8):12D-16D.

5. Investigators S, Yusuf S, Pitt B, Davis CE, Hood WB, Cohn JN. Effect of enalapril on survival in patients with reduced left ventricular ejection fractions and congestive heart failure. N Engl J Med 1991;325(5):293-302.

6. Colfer HT, Ribner HS, Gradman A, Hughes CV, Kapoor A, Laidlaw JC. Effects of once-daily benazepril therapy on exercise tolerance and manifestations of chronic congestive heart failure. The Benazepril Heart Failure Study Group. Am J Cardiol 1992;70(3):354-8.

7. Investigators S, Yusuf S, Pitt B, Davis CE, Hood WB, Jr., Cohn JN. Effect of enalapril on mortality and the development of heart failure in asymptomatic patients with reduced left ventricular ejection fractions. N Engl J Med 1992;327(10):685-91.

8. Kleber FX, Niemoller L. Long-term survival in the Munich Mild Heart Failure Trial (MHFT). Am J Cardiol 1993;71(13):1237-9.

9. A randomized trial of beta-blockade in heart failure. The Cardiac Insufficiency Bisoprolol Study (CIBIS). CIBIS Investigators and Committees. Circulation 1994;90(4):1765-73.

10. Beller B, Bulle T, Bourge RC, Colfer H, Fowles RE, Giles TD, Grover J, Whipple JP, Fisher MB, Jessup M, et al. Lisinopril versus placebo in the treatment of heart failure: the Lisinopril Heart Failure Study Group. J Clin Pharmacol 1995;35(7):673-80.

11. Brown EJ, Jr., Chew PH, MacLean A, Gelperin K, Ilgenfritz JP, Blumenthal M. Effects of fosinopril on exercise tolerance and clinical deterioration in patients with chronic congestive heart failure not taking digitalis. Fosinopril Heart Failure Study Group. Am J Cardiol 1995;75(8):596-600.

12. Dickstein K, Chang P, Willenheimer R, Haunso S, Remes J, Hall C, Kjekshus J. Comparison of the effects of losartan and enalapril on clinical status and exercise performance in patients with moderate or severe chronic heart failure. J Am Coll Cardiol 1995;26(2):438-45.

13. Erhardt L, MacLean A, Ilgenfritz J, Gelperin K, Blumenthal M. Fosinopril attenuates clinical deterioration and improves exercise tolerance in patients with heart failure. Fosinopril Efficacy/Safety Trial (FEST) Study Group. Eur Heart J 1995;16(12):1892-9.

14. Krum H, Sackner-Bernstein JD, Goldsmith RL, Kukin ML, Schwartz B, Penn J, Medina N, Yushak M, Horn E, Katz SD, et al. Double-blind, placebo-controlled study of the long-term efficacy of carvedilol in patients with severe chronic heart failure. Circulation 1995;92(6):1499-506.

15. Widimsky J, Kremer HJ, Jerie P, Uhlir O. Czech and Slovak spirapril intervention study (CASSIS). A randomized, placebo and active-controlled, double-blind multicentre trial in patients with congestive heart failure. Eur J Clin Pharmacol 1995;49(1-2):95-102.

16. Bristow MR, Gilbert EM, Abraham WT, Adams KF, Fowler MB, Hershberger RE, Kubo SH, Narahara KA, Ingersoll H, Krueger S, Young S, Shusterman N. Carvedilol produces dose-related improvements in left ventricular function and survival in subjects with chronic heart failure. MOCHA Investigators. Circulation 1996;94(11):2807-16.

17. Colucci WS, Packer M, Bristow MR, Gilbert EM, Cohn JN, Fowler MB, Krueger SK, Hershberger R, Uretsky BF, Bowers JA, Sackner-Bernstein JD, Young ST, Holcslaw TL, Lukas MA. Carvedilol inhibits clinical progression in patients with mild symptoms of heart failure. US Carvedilol Heart Failure Study Group. Circulation 1996;94(11):2800-6.

18. Packer M, Bristow MR, Cohn JN, Colucci WS, Fowler MB, Gilbert EM, Shusterman NH. The effect of carvedilol on morbidity and mortality in patients with chronic heart failure. U.S. Carvedilol Heart Failure Study Group. N Engl J Med 1996;334(21):1349-55.

19. Packer M, Colucci WS, Sackner-Bernstein JD, Liang CS, Goldscher DA, Freeman I, Kukin ML, Kinhal V, Udelson JE, Klapholz M, Gottlieb SS, Pearle D, Cody RJ, Gregory JJ, Kantrowitz NE, LeJemtel TH, Young ST, Lukas MA, Shusterman NH. Double-blind, placebo-controlled study of the effects of carvedilol in patients with moderate to severe heart failure. The PRECISE Trial. Prospective Randomized Evaluation of Carvedilol on Symptoms and Exercise. Circulation 1996;94(11):2793-9.

20. Randomised, placebo-controlled trial of carvedilol in patients with congestive heart failure due to ischaemic heart disease. Australia/New Zealand Heart Failure Research Collaborative Group. Lancet 1997;349(9049):375-80.

21. Cohn JN, Fowler MB, Bristow MR, Colucci WS, Gilbert EM, Kinhal V, Krueger SK, Lejemtel T, Narahara KA, Packer M, Young ST, Holcslaw TL, Lukas MA. Safety and efficacy of carvedilol in severe heart failure. The U.S. Carvedilol Heart Failure Study Group. J Card Fail 1997;3(3):173-9.

22. Digitalis Investigation G. The effect of digoxin on mortality and morbidity in patients with heart failure. N Engl J Med 1997;336(8):525-33.

23. Lang RM, Elkayam U, Yellen LG, Krauss D, McKelvie RS, Vaughan DE, Ney DE, Makris L, Chang PI. Comparative effects of losartan and enalapril on exercise capacity and clinical status in patients with heart failure. The Losartan Pilot Exercise Study Investigators. J Am Coll Cardiol 1997;30(4):983-91.

24. Pitt B, Segal R, Martinez FA, Meurers G, Cowley AJ, Thomas I, Deedwania PC, Ney DE, Snavely DB, Chang PI. Randomised trial of losartan versus captopril in patients over 65 with heart failure (Evaluation of Losartan in the Elderly Study, ELITE). Lancet 1997;349(9054):747-52.

25. van Veldhuisen DJ, Genth-Zotz S, Brouwer J, Boomsma F, Netzer T, Man In TVAJ, Pinto YM, Lie KI, Crijns HJ. High- versus low-dose ACE inhibition in chronic heart failure: a double-blind, placebo-controlled study of imidapril. J Am Coll Cardiol 1998;32(7):1811-8.

26. The Cardiac Insufficiency Bisoprolol Study II (CIBIS-II): a randomised trial. Lancet 1999;353(9146):9-13.

27. Goldstein S, Kennedy HL, Hall C, Anderson JL, Gheorghiade M, Gottlieb S, Jessup M, Karlsberg RP, Friday G, Haskell L. Metoprolol CR/XL in patients with heart failure: A pilot study examining the tolerability, safety, and effect on left ventricular ejection fraction. Am Heart J 1999;138(6 Pt 1):1158-65.

28. Hamroff G, Katz SD, Mancini D, Blaufarb I, Bijou R, Patel R, Jondeau G, Olivari MT, Thomas S, Le Jemtel TH. Addition of angiotensin II receptor blockade to maximal angiotensin-converting enzyme inhibition improves exercise capacity in patients with severe congestive heart failure. Circulation 1999;99(8):990-2.

29. Pitt B, Zannad F, Remme WJ, Cody R, Castaigne A, Perez A, Palensky J, Wittes J. The effect of spironolactone on morbidity and mortality in patients with severe heart failure. Randomized Aldactone Evaluation Study Investigators. N Engl J Med 1999;341(10):709-17.

30. Riegger GA, Bouzo H, Petr P, Munz J, Spacek R, Pethig H, von Behren V, George M, Arens H. Improvement in exercise tolerance and symptoms of congestive heart failure during treatment with candesartan cilexetil. Symptom, Tolerability, Response to Exercise Trial of Candesartan Cilexetil in Heart Failure (STRETCH) Investigators. Circulation 1999;100(22):2224-30.

31. Shettigar U, Hare T, Gelperin K, Ilgenfritz JP, Deitchman D, Blumenthal M. Effects of fosinopril on exercise tolerance, symptoms, and clinical outcomes in patients with decompensated heart failure. Congest Heart Fail 1999;5(1):27-34.

32. Effects of metoprolol CR in patients with ischemic and dilated cardiomyopathy : the randomized evaluation of strategies for left ventricular dysfunction pilot study. Circulation 2000;101(4):378-84.

33. Genth-Zotz S, Zotz RJ, Sigmund M, Hanrath P, Hartmann D, Bohm M, Waagstein F, Treese N, Meyer J, Darius H. MIC trial: metoprolol in patients with mild to moderate heart failure: effects on ventricular function and cardiopulmonary exercise testing. Eur J Heart Fail 2000;2(2):175-81.

34. Granger CB, Ertl G, Kuch J, Maggioni AP, McMurray J, Rouleau JL, Stevenson LW, Swedberg K, Young J, Yusuf S, Califf RM, Bart BA, Held P, Michelson EL, Sellers MA, Ohlin G, Sparapani R, Pfeffer MA. Randomized trial of candesartan cilexetil in the treatment of patients with congestive heart failure and a history of intolerance to angiotensin-converting enzyme inhibitors. Am Heart J 2000;139(4):609-17.

35. Hjalmarson A, Goldstein S, Fagerberg B, Wedel H, Waagstein F, Kjekshus J, Wikstrand J, El Allaf D, Vitovec J, Aldershvile J, Halinen M, Dietz R, Neuhaus KL, Janosi A, Thorgeirsson G, Dunselman PH, Gullestad L, Kuch J, Herlitz J, Rickenbacher P, Ball S, Gottlieb S, Deedwania P. Effects of controlled-release metoprolol on total mortality, hospitalizations, and well-being in patients with heart failure: the Metoprolol CR/XL Randomized Intervention Trial in congestive heart failure (MERIT-HF). MERIT-HF Study Group. JAMA 2000;283(10):1295-302.

36. Pitt B, Poole-Wilson PA, Segal R, Martinez FA, Dickstein K, Camm AJ, Konstam MA, Riegger G, Klinger GH, Neaton J, Sharma D, Thiyagarajan B. Effect of losartan compared with captopril on mortality in patients with symptomatic heart failure: randomised trial--the Losartan Heart Failure Survival Study ELITE II. Lancet 2000;355(9215):1582-7.

37. Sturm B, Pacher R, Strametz-Juranek J, Berger R, Frey B, Stanek B. Effect of beta 1 blockade with atenolol on progression of heart failure in patients pretreated with high-dose enalapril. Eur J Heart Fail 2000;2(4):407-12.

38. Witchitz S, Cohen-Solal A, Dartois N, Weisslinger N, Juste K, Darmon JY. Treatment of heart failure with celiprolol, a cardioselective beta blocker with beta-2 agonist vasodilatory properties. The CELICARD Group. Am J Cardiol 2000;85(12):1467-71.

39. Beta-Blocker Evaluation of Survival Trial I, Eichhorn EJ, Domanski MJ, Krause-Steinrauf H, Bristow MR, Lavori PW. A trial of the beta-blocker bucindolol in patients with advanced chronic heart failure. N Engl J Med 2001;344(22):1659-67.

40. Cohn JN, Tognoni G, Valsartan Heart Failure Trial I. A randomized trial of the angiotensin-receptor blocker valsartan in chronic heart failure. N Engl J Med 2001;345(23):1667-75.

41. Dunselman PH, Replacement of Angiotensin Converting Enzyme Inhibition I. Effects of the replacement of the angiotensin converting enzyme inhibitor enalapril by the angiotensin II receptor blocker telmisartan in patients with congestive heart failure. The replacement of angiotensin converting enzyme inhibition (REPLACE) investigators. Int J Cardiol 2001;77(2-3):131-8; discussion 139-40.

42. Cicoira M, Zanolla L, Rossi A, Golia G, Franceschini L, Brighetti G, Marino P, Zardini P. Long-term, dose-dependent effects of spironolactone on left ventricular function and exercise tolerance in patients with chronic heart failure. J Am Coll Cardiol 2002;40(2):304-10.

43. de Milliano PA, de Groot AC, Tijssen JG, van Eck-Smit BL, Van Zwieten PA, Lie KI. Beneficial effects of metoprolol on myocardial sympathetic function: Evidence from a randomized, placebo-controlled study in patients with congestive heart failure. Am Heart J 2002;144(2):E3.

44. Dubach P, Myers J, Bonetti P, Schertler T, Froelicher V, Wagner D, Scheidegger M, Stuber M, Luchinger R, Schwitter J, Hess O. Effects of bisoprolol fumarate on left ventricular size, function, and exercise capacity in patients with heart failure: analysis with magnetic resonance myocardial tagging. Am Heart J 2002;143(4):676-83.

45. Packer M, Fowler MB, Roecker EB, Coats AJ, Katus HA, Krum H, Mohacsi P, Rouleau JL, Tendera M, Staiger C, Holcslaw TL, Amann-Zalan I, DeMets DL, Carvedilol Prospective Randomized Cumulative Survival Study G. Effect of carvedilol on the morbidity of patients with severe chronic heart failure: results of the carvedilol prospective randomized cumulative survival (COPERNICUS) study. Circulation 2002;106(17):2194-9.

46. Willenheimer R, Helmers C, Pantev E, Rydberg E, Lofdahl P, Gordon A, Heart Failure Valsartan Exercise Capacity Evaluation Study G. Safety and efficacy of valsartan versus enalapril in heart failure patients. Int J Cardiol 2002;85(2-3):261-70.

47. Granger CB, McMurray JJ, Yusuf S, Held P, Michelson EL, Olofsson B, Ostergren J, Pfeffer MA, Swedberg K, Investigators C, Committees. Effects of candesartan in patients with chronic heart failure and reduced left-ventricular systolic function intolerant to angiotensin-converting-enzyme inhibitors: the CHARM-Alternative trial. Lancet 2003;362(9386):772-6.

48. McKelvie RS, Rouleau JL, White M, Afzal R, Young JB, Maggioni AP, Held P, Yusuf S. Comparative impact of enalapril, candesartan or metoprolol alone or in combination on ventricular remodelling in patients with congestive heart failure. Eur Heart J 2003;24(19):1727-34.

49. McMurray JJ, Ostergren J, Swedberg K, Granger CB, Held P, Michelson EL, Olofsson B, Yusuf S, Pfeffer MA, Investigators C, Committees. Effects of candesartan in patients with chronic heart failure and reduced left-ventricular systolic function taking angiotensin-converting-enzyme inhibitors: the CHARM-Added trial. Lancet 2003;362(9386):767-71.

50. Mitrovic V, Willenbrock R, Miric M, Seferovic P, Spinar J, Dabrowski M, Kiowski W, Marks DS, Alegria E, Dukat A, Lenz K, Arens HA. Acute and 3-month treatment effects of candesartan cilexetil on hemodynamics, neurohormones, and clinical symptoms in patients with congestive heart failure. Am Heart J 2003;145(3):E14.

51. Cohen Solal A, Jondeau G, Beauvais F, Berdeaux A. Beneficial effects of carvedilol on angiotensin-converting enzyme activity and renin plasma levels in patients with chronic heart failure. Eur J Heart Fail 2004;6(4):463-6.

52. Komajda M, Lutiger B, Madeira H, Thygesen K, Bobbio M, Hildebrandt P, Jaarsma W, Riegger G, Ryden L, Scherhag A, Soler-Soler J, Remme WJ, investigators C, and co-ordinators. Tolerability of carvedilol and ACE-Inhibition in mild heart failure. Results of CARMEN (Carvedilol ACE-Inhibitor Remodelling Mild CHF EvaluatioN). Eur J Heart Fail 2004;6(4):467-75.

53. Edes I, Gasior Z, Wita K. Effects of nebivolol on left ventricular function in elderly patients with chronic heart failure: results of the ENECA study. Eur J Heart Fail 2005;7(4):631-9.

54. Palazzuoli A, Quatrini I, Vecchiato L, Calabria P, Gennari L, Martini G, Nuti R. Left ventricular diastolic function improvement by carvedilol therapy in advanced heart failure. J Cardiovasc Pharmacol 2005;45(6):563-8.

55. Palazzuoli A, Quatrini I, Vecchiato L, Scali C, De Paola V, Iovine F, Martini G, Nuti R. Effects of carvedilol on left ventricular diastolic function and chamber volumes in advanced heart failure. Minerva Cardioangiol 2005;53(4):321-8.

56. Willenheimer R, van Veldhuisen DJ, Silke B, Erdmann E, Follath F, Krum H, Ponikowski P, Skene A, van de Ven L, Verkenne P, Lechat P, Investigators CI. Effect on survival and hospitalization of initiating treatment for chronic heart failure with bisoprolol followed by enalapril, as compared with the opposite sequence: results of the randomized Cardiac Insufficiency Bisoprolol Study (CIBIS) III. Circulation 2005;112(16):2426-35.

57. Boccanelli A, Mureddu GF, Cacciatore G, Clemenza F, Di Lenarda A, Gavazzi A, Porcu M, Latini R, Lucci D, Maggioni AP, Masson S, Vanasia M, de Simone G, Investigators AI-C. Anti-remodelling effect of canrenone in patients with mild chronic heart failure (AREA IN-CHF study): final results. Eur J Heart Fail 2009;11(1):68-76.

58. Swedberg K, Komajda M, Bohm M, Borer JS, Ford I, Dubost-Brama A, Lerebours G, Tavazzi L, Investigators S. Ivabradine and outcomes in chronic heart failure (SHIFT): a randomised placebo-controlled study. Lancet 2010;376(9744):875-85.

59. Zannad F, McMurray JJ, Krum H, van Veldhuisen DJ, Swedberg K, Shi H, Vincent J, Pocock SJ, Pitt B, Group E-HS. Eplerenone in patients with systolic heart failure and mild symptoms. N Engl J Med 2011;364(1):11-21.

60. McMurray JJ, Packer M, Desai AS, Gong J, Lefkowitz MP, Rizkala AR, Rouleau JL, Shi VC, Solomon SD, Swedberg K, Zile MR, Investigators P-H, Committees. Angiotensin-neprilysin inhibition versus enalapril in heart failure. N Engl J Med 2014;371(11):993-1004.

61. Vizzardi E, Nodari S, Caretta G, D'Aloia A, Pezzali N, Faden G, Lombardi C, Raddino R, Metra M, Dei Cas L. Effects of spironolactone on long-term mortality and morbidity in patients with heart failure and mild or no symptoms. Am J Med Sci 2014;347(4):271-6.

62. Gheorghiade M, Greene SJ, Butler J, Filippatos G, Lam CS, Maggioni AP, Ponikowski P, Shah SJ, Solomon SD, Kraigher-Krainer E, Samano ET, Muller K, Roessig L, Pieske B, Investigators S-R, Coordinators. Effect of Vericiguat, a Soluble Guanylate Cyclase Stimulator, on Natriuretic Peptide Levels in Patients With Worsening Chronic Heart Failure and Reduced Ejection Fraction: The SOCRATES-REDUCED Randomized Trial. JAMA 2015;314(21):2251-62.

63. Teerlink JR, Felker GM, McMurray JJ, Solomon SD, Adams KF, Jr., Cleland JG, Ezekowitz JA, Goudev A, Macdonald P, Metra M, Mitrovic V, Ponikowski P, Serpytis P, Spinar J, Tomcsanyi J, Vandekerckhove HJ, Voors AA, Monsalvo ML, Johnston J, Malik FI, Honarpour N, Investigators C-H. Chronic Oral Study of Myosin Activation to Increase Contractility in Heart Failure (COSMIC-HF): a phase 2, pharmacokinetic, randomised, placebo-controlled trial. Lancet 2016;388(10062):2895-2903.

64. McMurray JJV, Solomon SD, Inzucchi SE, Kober L, Kosiborod MN, Martinez FA, Ponikowski P, Sabatine MS, Anand IS, Belohlavek J, Bohm M, Chiang CE, Chopra VK, de Boer RA, Desai AS, Diez M, Drozdz J, Dukat A, Ge J, Howlett JG, Katova T, Kitakaze M, Ljungman CEA, Merkely B, Nicolau JC, O'Meara E, Petrie MC, Vinh PN, Schou M, Tereshchenko S, Verma S, Held C, DeMets DL, Docherty KF, Jhund PS, Bengtsson O, Sjostrand M, Langkilde AM, Committees D-HT, Investigators. Dapagliflozin in Patients with Heart Failure and Reduced Ejection Fraction. N Engl J Med 2019;381(21):1995-2008.

65. Nassif ME, Windsor SL, Tang F, Khariton Y, Husain M, Inzucchi SE, McGuire DK, Pitt B, Scirica BM, Austin B, Drazner MH, Fong MW, Givertz MM, Gordon RA, Jermyn R, Katz SD, Lamba S, Lanfear DE, LaRue SJ, Lindenfeld J, Malone M, Margulies K, Mentz RJ, Mutharasan RK, Pursley M, Umpierrez G, Kosiborod M. Dapagliflozin Effects on Biomarkers, Symptoms, and Functional Status in Patients With Heart Failure With Reduced Ejection Fraction: The DEFINE-HF Trial. Circulation 2019;140(18):1463-1476.

66. Armstrong PW, Pieske B, Anstrom KJ, Ezekowitz J, Hernandez AF, Butler J, Lam CSP, Ponikowski P, Voors AA, Jia G, McNulty SE, Patel MJ, Roessig L, Koglin J, O'Connor CM, Group VS. Vericiguat in Patients with Heart Failure and Reduced Ejection Fraction. N Engl J Med 2020;382(20):1883-1893.

67. Jensen J, Omar M, Kistorp C, Poulsen MK, Tuxen C, Gustafsson I, Kober L, Gustafsson F, Faber J, Fosbol EL, Bruun NE, Brond JC, Forman JL, Videbaek L, Moller JE, Schou M. Twelve weeks of treatment with empagliflozin in patients with heart failure and reduced ejection fraction: A double-blinded, randomized, and placebo-controlled trial. Am Heart J 2020;228:47-56.

68. Packer M, Anker SD, Butler J, Filippatos G, Pocock SJ, Carson P, Januzzi J, Verma S, Tsutsui H, Brueckmann M, Jamal W, Kimura K, Schnee J, Zeller C, Cotton D, Bocchi E, Bohm M, Choi DJ, Chopra V, Chuquiure E, Giannetti N, Janssens S, Zhang J, Gonzalez Juanatey JR, Kaul S, Brunner-La Rocca HP, Merkely B, Nicholls SJ, Perrone S, Pina I, Ponikowski P, Sattar N, Senni M, Seronde MF, Spinar J, Squire I, Taddei S, Wanner C, Zannad F, Investigators EM-RT. Cardiovascular and Renal Outcomes with Empagliflozin in Heart Failure. N Engl J Med 2020;383(15):1413-1424.

69. Teerlink JR, Diaz R, Felker GM, McMurray JJV, Metra M, Solomon SD, Adams KF, Anand I, Arias-Mendoza A, Biering-Sorensen T, Bohm M, Bonderman D, Cleland JGF, Corbalan R, Crespo-Leiro MG, Dahlstrom U, Echeverria LE, Fang JC, Filippatos G, Fonseca C, Goncalvesova E, Goudev AR, Howlett JG, Lanfear DE, Li J, Lund M, Macdonald P, Mareev V, Momomura SI, O'Meara E, Parkhomenko A, Ponikowski P, Ramires FJA, Serpytis P, Sliwa K, Spinar J, Suter TM, Tomcsanyi J, Vandekerckhove H, Vinereanu D, Voors AA, Yilmaz MB, Zannad F, Sharpsten L, Legg JC, Varin C, Honarpour N, Abbasi SA, Malik FI, Kurtz CE, Investigators G-H. Cardiac Myosin Activation with Omecamtiv Mecarbil in Systolic Heart Failure. N Engl J Med 2020.

**Table S4. Reported concomitant medical and device therapy for the selected randomized controlled trials.**

| **RCT**  **(year)** | **ACEi/ARB** | **BB** | **MRA** | **Diuretics** | **ARNI** | **ICD** | **CRT** |
| --- | --- | --- | --- | --- | --- | --- | --- |
| **Chalmers**  **(1987)** | NA | NR | 12 | 86 | NR | NR | NR |
| **CONSENSUS**  **(1987)** | NA | 3 | 52 | 98 | NR | NR | NR |
| **The Captopril-Digoxin Multicenter Research Group**  **(1988)** | NA | NR | NR | NR | NR | NR | NR |
| **Lewis**  **(1989)** | NA | NR | NR | NR | NR | NR | NR |
| **SOLVD**  **(1991)** | NA | 7 | 9 | 85 | NR | NR | NR |
| **SOLVD**  **(1992)** | NA | 24 | 4 | 17 | NR | NR | NR |
| **Colfer**  **(1992)** | NA | NR | NR | NR | NR | NR | NR |
| **MHFT**  **(1993)** | NA | NR | NR | NR | NR | NR | NR |
| **CIBIS**  **(1994)** | 90 | NA | NR | 100 | NR | NR | NR |
| **CASSIS**  **(1995)** | NA | NR | NR | 96 | NR | NR | NR |
| **Beller**  **(1995)** | NA | NR | NR | NR | NR | NR | NR |
| **Brown**  **(1995)** | NA | NR | NR | NR | NR | NR | NR |
| **Dickstein**  **(1995)** | NA | 12 | NR | 95 | NR | NR | NR |
| **FEST**  **(1995)** | NA | NR | NR | NR | NR | NR | NR |
| **Krum**  **(1995)** | NR | NA | NR | NR | NR | NR | NR |
| **Packer**  **(1996)** | 95 | NA | NR | 95 | NR | NR | NR |
| **PRECISE**  **(1996)** | 96 | NA | NR | 98 | NR | NR | NR |
| **MOCHA**  **(1996)** | 94 | NA | NR | 95 | NR | NR | NR |
| **Colucci**  **(1996)** | NR | NA | NR | NR | NR | NR | NR |
| **ANZ HF Coll GROUP**  **(1997)** | 85 | NA | NR | 75 | NR | NR | NR |
| **Lang**  **(1997)** | 7 | NA | NR | 99 | NR | NR | NR |
| **Cohn**  **(1997)** | 92 | NA | NR | 98 | NR | NR | NR |
| **The Digitalis Investigation Group**  **(1997)** | 94 | NR | NR | 82 | NR | NR | NR |
| **ELITE I**  **(1997)** | NA | 16 | NR | 74 | NR | NR | NR |
| **Van Veldhuisen**  **(1998)** | NA | NR | NR | 28 | NR | NR | NR |
| **CIBIS-II**  **(1999)** | 96 | NA | NR | 98 | NR | NR | NR |
| **STRETCH**  **(1999)** | NA | 0 | 1 | 60 | NR | NR | NR |
| **Hamroff**  **(1999)** | NA | 6 | NR | 100 | NR | NR | NR |
| **MERIT-HF Pilot**  **(1999)** | 92 | NA | NR | 86 | NR | NR | NR |
| **RALES**  **(1999)** | 94 | 10 | NA | 100 | NR | NR | NR |
| **Shettigar**  **(1999)** | NA | NR | NR | NR | NR | NR | NR |
| **RESOLVD**  **(2000)** | 59 | NA | NR | 83 | NR | NR | NR |
| **ELITE II**  **(2000)** | NA | 22 | 21 | 78 | NR | NR | NR |
| **CELICARD**  **(2000)** | 85 | NA | NR | 94 | NR | NR | NR |
| **MIC**  **(2000)** | 91 | NA | NR | 71 | NR | NR | NR |
| **SPICE**  **(2000)** | NA | 21 | NR | 74 | NR | 3 | NR |
| **Sturm**  **(2000)** | 100 | NA | NR | 54 | NR | NR | NR |
| **REPLACE**  **(2001)** | NA | NR | NR | NR | NR | NR | NR |
| **Val-HeFT**  **(2001)** | 93 | 35 | NR | 85 | NR | NR | NR |
| **COPERNICUS**  **(2001)** | 97 | NA | 22 | 99 | NR | NR | NR |
| **BEST**  **(2001)** | 97 | NA | 3 | 94 | NR | NR | NR |
| **Dubach**  **(2002)** | 100 | NA | NR | 85 | NR | NR | NR |
| **De Milliano**  **(2002)** | 92 | NA | NR | NR | NR | NR | NR |
| **Cicoiria**  **(2002)** | NR | 69 | NA | NR | NR | NR | NR |
| **COPERNICUS**  **(2002)** | NR | NA | NR | NR | NR | NR | NR |
| **HEAVEN**  **(2002)** | 99 | 54 | NR | 84 | NR | NR | NR |
| **Mitrovic**  **(2003)** | NA | NR | NR | 91 | NR | NR | NR |
| **RESOLVD**  **(2003)** | 92 | NR | NR | 83 | NR | NR | NR |
| **CHARM-Added**  **(2003)** | 100 | 55 | 17 | 90 | NR | 4 | NR |
| **CHARM-Alternative**  **(2003)** | NA | 55 | 24 | 85 | NR | 3 | NR |
| **SYMPOXYDEX**  **(2004)** | 95 | NA | NR | 100 | NR | NR | NR |
| **ENECA**  **(2004)** | 96 | NA | NR | 88 | NR | NR | NR |
| **CARMEN**  **(2004)** | 66 | 6 | 13 | 71 | NR | NR | NR |
| **CIBIS III**  **(2005)** | 13 | NA | 10 | 84 | NR | NR | NR |
| **Palazzuoli**  **(2005)** | NR | NA | NR | NR | NR | NR | NR |
| **Palazzuoli, b**  **(2005)** | NR | NA | NR | NR | NR | NR | NR |
| **MERIT-HF**  **(2009)** | 95 | NA | NR | 90 | NR | NR | NR |
| **AREA IN-CHF**  **(2009)** | 96 | 80 | NR | 70 | NR | 6 | NR |
| **SHIFT**  **(2010)** | 89 | 92 | 60 | 83 | NR | 3 | 1 |
| **EMPHASIS-HF**  **(2011)** | 93 | 87 | NR | 85 | NR | 13 | 2 |
| **Vizzardi**  **(2014)** | 99 | 97 | NR | 81 | NR | 69 | 42 |
| **PARADIGM-HF**  **(2014)** | NA | 93 | 56 | 80 | NR | 15 | 7 |
| **SOCRATES-REDUCED**  **(2015)** | 84 | 90 | 62 | 94 | NR | 21 | 7 |
| **COSMIC-HF**  **(2016)** | 93 | 97 | 57 | 84 | NR | 42 | 2 |
| **DAPA-HF**  **(2019)** | 84 | 96 | 71 | 93 | 11 | 26 | 7 |
| **DEFINE-HF**  **(2019)** | 59 | 96 | 61 | 85 | 32 | 62 | 26 |
| **EMPIRE-HF**  **(2020)** | 96 | 95 | 65 | 63 | 30 | 47 | 19 |
| **VICTORIA**  **(2020)** | 73 | 93 | 70 | NR | 14 | 28 | 15 |
| **GALACTIC-HF**  **(2020)** | 87 | 94 | 78 | NR | 19 | 32 | 14 |
| **EMPEROR-REDUCED**  **(2020)** | 70 | 95 | 71 | NR | 19 | 31 | 12 |

ACEi: angiotensin converting enzyme inhibitor; ARB: angiotensin receptor blocker; ARNI: angiotensin receptor-neprilysin inhibitor; BB: beta-blocker; CRT: cardiac resynchronization therapy; ICD: implantable cardioverter defibrillator; MRA: mineralocorticoid receptor antagonist; NR: not reported; RCT: randomized controlled trial.

**Table S5. Risk of bias assessment in the studies included in the network meta-analysis and quality of evidence for the pairwise comparisons.**

| **RCT**  **(year)** | **Random sequence generation**  **(Selection bias)** | **Allocation concealment (Selection bias)** | **Blinding of participants, personnel and outcome (Performance bias)** | **Incomplete outcome data**  **(Attrition bias)** | **Selective reporting (Reporting bias)** | **Other bias** |
| --- | --- | --- | --- | --- | --- | --- |
| **Chalmers**  **(1987)** | Unclear | Unclear | Low | High | Low | Low |
| **CONSENSUS**  **(1987)** | Low | Low | Low | Low | Low | Low |
| **The Captopril-Digoxin Multicenter Research Group**  **(1988)** | Low | Unclear | Low | Low | Low | Low |
| **Lewis**  **(1989)** | Unclear | Unclear | Low | Unclear | Low | Unclear |
| **SOLVD**  **(1991)** | Low | Low | Low | Unclear | Low | Low |
| **SOLVD**  **(1992)** | Unclear | Unclear | Low | Unclear | Low | Low |
| **Colfer**  **(1992)** | Unclear | Unclear | Low | Low | Low | High |
| **MHFT**  **(1993)** | Low | Low | Low | Low | Low | Unclear |
| **CIBIS**  **(1994)** | Low | Low | Low | Low | Low | Low |
| **CASSIS**  **(1995)** | Unclear | Unclear | Low | Unclear | Low | High |
| **Beller**  **(1995)** | Unclear | Unclear | Low | High | Low | Low |
| **Brown**  **(1995)** | Unclear | Unclear | Low | Unclear | Low | Low |
| **Dickstein**  **(1995)** | Unclear | Unclear | Low | Unclear | Low | Low |
| **FEST**  **(1995)** | Unclear | Unclear | Low | Unclear | Low | Low |
| **Krum**  **(1995)** | Unclear | Unclear | Low | Low | Low | Low |
| **Packer**  **(1996)** | Low | Unclear | Low | Low | Low | Low |
| **PRECISE**  **(1996)** | Unclear | Unclear | Low | Low | Low | Low |
| **MOCHA**  **(1996)** | Unclear | Unclear | Low | Low | Low | Low |
| **Colucci**  **(1996)** | Unclear | Unclear | Low | Low | Low | Low |
| **ANZ HF Coll GROUP**  **(1997)** | Low | Unclear | Low | Unclear | Low | Low |
| **Lang**  **(1997)** | Low | Unclear | Low | Low | Low | Low |
| **Cohn**  **(1997)** | Unclear | Unclear | Low | Low | Low | Low |
| **The Digitalis**  **Investigation**  **Group**  **(1997)** | Low | Unclear | Low | Low | Low | Low |
| **ELITE I**  **(1997)** | Low | Unclear | Low | Low | Low | Low |
| **Van Veldhuisen**  **(1998)** | Unclear | Unclear | Low | Low | Low | Low |
| **CIBIS-II**  **(1999)** | Low | Low | Low | Low | Low | Low |
| **STRETCH**  **(1999)** | Low | Low | Low | Low | Low | Low |
| **Hamroff**  **(1999)** | Unclear | Unclear | Low | Unclear | Low | Low |
| **MERIT-HF Pilot**  **(1999)** | Unclear | Low | Low | High | Low | High |
| **RALES**  **(1999)** | Unclear | Unclear | Low | Low | Low | Low |
| **Shettigar**  **(1999)** | Unclear | Unclear | Low | Unclear | Low | Low |
| **RESOLVD**  **(2000)** | Low | Unclear | Low | Low | Low | Low |
| **ELITE II**  **(2000)** | Low | Unclear | Low | Low | Low | Low |
| **CELICARD**  **(2000)** | Low | Unclear | Low | Low | Low | Low |
| **MIC**  **(2000)** | Unclear | Unclear | Low | High | Low | Low |
| **SPICE**  **(2000)** | Unclear | Unclear | Low | Low | Low | Low |
| **Sturm**  **(2000)** | Unclear | Unclear | Low | Low | Low | High |
| **REPLACE**  **(2001)** | Unclear | Unclear | Low | Unclear | Low | Low |
| **Val-HeFT**  **(2001)** | Low | Unclear | Low | Unclear | Low | Low |
| **BEST**  **(2001)** | Low | Low | Low | Low | Low | Low |
| **Dubach**  **(2002)** | Low | low | Low | Unclear | Low | Unclear |
| **De Milliano**  **(2002)** | Low | Unclear | Low | Unclear | Low | Low |
| **Cicoria**  **(2002)** | Unclear | Unclear | High | Unclear | Low | Low |
| **COPERNICUS**  **(2002)** | Low | Low | Low | Low | Low | Low |
| **HEAVEN**  **(2002)** | Unclear | Unclear | Low | Low | Low | Low |
| **Mitrovic**  **(2003)** | Unclear | Unclear | Low | Low | Low | Low |
| **RESOLVD**  **(2003)** | Low | Unclear | Low | Low | Low | Low |
| **CHARM-Added**  **(2003)** | Low | Low | Low | Low | Low | Low |
| **CHARM-Alternative**  **(2003)** | Low | Low | Low | Low | Low | Low |
| **SYMPOXYDEX**  **(2004)** | Unclear | Unclear | Low | Low | Low | Low |
| **ENECA**  **(2004)** | Unclear | low | Low | Low | Low | Low |
| **CARMEN**  **(2004)** | Low | Low | Low | Low | Low | Low |
| **CIBIS III**  **(2005)** | Low | Low | High | Low | Low | Low |
| **Palazzuoli**  **(2005)** | Unclear | Unclear | Low | Unclear | Low | Low |
| **Palazzuoli, b**  **(2005)** | Unclear | Unclear | Low | Unclear | Low | Low |
| **MERIT-HF**  **(2009)** | Low | Low | Low | Low | Low | Low |
| **AREA IN-CHF**  **(2009)** | Unclear | Unclear | Low | Low | Low | Low |
| **SHIFT**  **(2010)** | Low | Low | Low | Low | Low | Low |
| **EMPHASIS-HF**  **(2011)** | Low | Low | Low | Low | Low | Low |
| **Vizzardi**  **(2014)** | Unclear | Low | Low | Unclear | Low | Low |
| **PARADIGM-HF**  **(2014)** | Low | Low | Low | Low | Low | Low |
| **SOCRATES-REDUCED**  **(2015)** | Low | Low | Low | Low | Low | Low |
| **COSMIC-HF**  **(2016)** | Low | Low | Low | Low | Low | Low |
| **DAPA-HF**  **(2019)** | Low | Low | Low | Low | Low | Low |
| **DEFINE-HF**  **(2019)** | Low | Low | Low | Low | Low | Low |
| **EMPIRE-HF**  **(2020)** | Low | Low | Low | Low | Low | Low |
| **VICTORIA**  **(2020)** | Low | Low | Low | Low | Low | Low |
| **GALACTIC-HF**  **(2020)** | Low | Low | Low | Low | Low | Low |
| **EMPEROR-REDUCED**  **(2020)** | Low | Low | Low | Low | Low | Low |

RCT: randomized controlled trial.

**Table S6. Number of events for the endpoints of interest in the included studies.**

| **Study characteristics** | | | **All-cause death** | **CV death** | **HHF** | **All-cause hospitalization** |
| --- | --- | --- | --- | --- | --- | --- |
| **RCT**  **(year)** | **Patients (n°)** | **Intervention** | **Events**  **(n°)** | **Events**  **(n°)** | **Events**  **(n°)** | **Events**  **(n°)** |
| **Chalmers**  **(1987)** | 87  43 | Lisinopril  Placebo | 4  3 | NR  NR | NR  NR | NR  NR |
| **CONSENSUS**  **(1987)** | 127  126 | Enalapril  Placebo | 46  66 | 44  64 | NR  NR | NR  NR |
| **The Captopril-Digoxin Multicenter Research Group**  **(1988)** | 104  96  100 | Captopril  Digoxin  Placebo | 8  7  6 | NR  NR  NR | 5  3  11 | NR  NR  NR |
| **Lewis**  **(1989)** | 87  43 | Lisinopril  Placebo | 4  3 | 2  0 | NR  NR | NR  NR |
| **SOLVD**  **(1991)** | 1285  1284 | Enalapril  Placebo | 452  510 | 399  461 | 729  810 | NR  NR |
| **SOLVD**  **(1992)** | 2111  2117 | Enalapril  Placebo | 313  334 | 265  298 | 184  273 | NR  NR |
| **Colfer**  **(1992)** | 114  58 | Benazepril  Placebo | 0  3 | NR  NR | NR  NR | NR  NR |
| **MHFT**  **(1993)** | 83  87 | Captopril  Placebo | 26  32 | NR  NR | NR  NR | NR  NR |
| **CIBIS**  **(1994)** | 320  321 | Bisoprolol  Placebo | 53  67 | 50  63 | 107  151 | NR  NR |
| **CASSIS**  **(1995)** | 152  48  48 | Spirapril  Enalapril  Placebo | 5  2  6 | NR  NR  NR | NR  NR  NR | NR  NR  NR |
| **Beller**  **(1995)** | 130  63 | Lisinopril  Placebo | 4  5 | NR  NR | NR  NR | NR  NR |
| **Brown**  **(1995)** | 116  125 | Fosinopril  Placebo | 3  4 | NR  NR | 6  12 | NR  NR |
| **Dickstein**  **(1995)** | 108  58 | Losartan  Enalapril | 2  2 | NR  NR | NR  NR | NR  NR |
| **FEST**  **(1995)** | 155  153 | Fosinopril  Placebo | 5  3 | NR  NR | 4  18 | NR  NR |
| **Krum**  **(1995)** | 33  16 | Carvedilol  Placebo | 2  2 | NR  NR | NR  NR | NR  NR |
| **Packer**  **(1996)** | 696  398 | Carvedilol  Placebo | 22  31 | 20  31 | 98  78 | NR  NR |
| **PRECISE**  **(1996)** | 133  145 | Carvedilol  Placebo | 6  11 | NR  NR | 19  34 | NR  NR |
| **MOCHA**  **(1996)** | 261  84 | Carvedilol  Placebo | 12  13 | 9  13 | 29  17 | NR  NR |
| **Colucci**  **(1996)** | 232  134 | Carvedilol  Placebo | 0  4 | 0  4 | 9  8 | NR  NR |
| **ANZ HF Coll GROUP**  **(1997)** | 207  208 | Carvedilol  Placebo | 20  26 | 18  20 | 23  33 | 99  120 |
| **Lang**  **(1997)** | 78  38 | Losartan  Enalapril | 6  0 | 3  0 | NR  NR | NR  NR |
| **Cohn**  **(1997)** | 70  35 | Carvedilol  Placebo | 2  2 | NR  NR | NR  NR | NR  NR |
| **The Digitalis Investigation Group**  **(1997)** | 3397  3403 | Digoxin  Placebo | 1181  1184 | 1016  1004 | 1694  1850 | NR  NR |
| **ELITE I**  **(1997)** | 352  370 | Losartan  Captopril | 17  32 | 12  24 | 20  21 | 78  110 |
| **Van Veldhuisen**  **(1998)** | 182  62 | Imidapril  Placebo | 2  1 | NR  NR | NR  NR | NR  NR |
| **CIBIS-II**  **(1999)** | 1327  1320 | Bisoprolol  Placebo | 156  228 | 119  161 | NR  NR | 440  513 |
| **STRETCH**  **(1999)** | 633  211 | Candesartan  Placebo | 10  1 | 8  1 | NR  NR | NR  NR |
| **Hamroff**  **(1999)** | 16  17 | Losartan  Placebo | 0  1 | NR  NR | NR  NR | NR  NR |
| **MERIT-HF Pilot**  **(1999)** | 42  19 | Metoprolol  Placebo | 0  1 | 0  0 | 2  4 | NR  NR |
| **RALES**  **(1999)** | 822  841 | Spironolactone  Placebo | 284  386 | 226  314 | 260  336 | 483  568 |
| **Shettigar**  **(1999)** | 102  104 | Fosinopril  Placebo | 2  3 | NR  NR | NR  NR | NR  NR |
| **RESOLVD**  **(2000)** | 214  212 | Metoprolol  Placebo | 8  17 | NR  NR | 15  5 | 34  36 |
| **ELITE II**  **(2000)** | 1578  1574 | Losartan  Captopril | 280  250 | 46  53 | 270  293 | 659  638 |
| **CELICARD**  **(2000)** | 62  62 | Celiprolol  Placebo | 3  6 | 2  3 | NR  NR | NR  NR |
| **MIC**  **(2000)** | 26  26 | Metoprolol  Placebo | 1  2 | 1  2 | NR  NR | NR  NR |
| **SPICE**  **(2000)** | 179  91 | Candesartan  Placebo | 6  3 | NR  NR | 15  11 | 23  17 |
| **Sturm**  **(2000)** | 51  49 | Atenolol + Enalapril  Placebo + Enalapril | 5  8 | NR  NR | 5  12 | NR  NR |
| **REPLACE**  **(2001)** | 301  77 | Telmisartan  Enalapril | 4  2 | 4  2 | NR  NR | NR  NR |
| **Val-HeFT**  **(2001)** | 2511  2499 | Valsartan  Placebo | 495  484 | 262  258 | 346  455 | NR  NR |
| **COPERNICUS**  **(2001)** | 1156  1133 | Carvedilol  Placebo | 130  190 | NR  NR | NR  NR | NR  NR |
| **BEST**  **(2001)** | 1354  1354 | Bucindolol  Placebo | 411  449 | 342  389 | 476  569 | 829  875 |
| **Dubach**  **(2002)** | 13  15 | Bisoprolol  Placebo | 1  0 | NR  NR | NR  NR | NR  NR |
| **De Milliano**  **(2002)** | 43  11 | Metoprolol  Placebo | 4  1 | 3  1 | NR  NR | NR  NR |
| **Cicoira**  **(2002)** | 54  52 | Spironolactone  Placebo | 3  4 | NR  NR | NR  NR | NR  NR |
| **COPERNICUS**  **(2002)** | 1156  1113 | Carvedilol  Placebo | 128  193 | NR  NR | 198  268 | 372  432 |
| **HEAVEN**  **(2002)** | 70  71 | Valsartan  Enalapril | 1  5 | 1  2 | NR  NR | NR  NR |
| **Mitrovic**  **(2003)** | 174  44 | Candesartan  Placebo | 5  2 | 5  2 | NR  NR | NR  NR |
| **RESOLVD**  **(2003)** | 125  89  86  126 | Candesartan + Metoprolol  or  Enalapril + Metoprolol  Candesartan + Enalapril + Metoprolol  Candesartan + Enalapril  Candesartan or Enalapril | 2  6  8  12 | NR  NR  NR  NR | 10  11  1  9 | NR  NR  NR  NR |
| **CHARM-Added**  **(2003)** | 1276  1272 | Candesartan  Placebo | 377  412 | 302  347 | 309  356 | NR  NR |
| **CHARM-Alternative**  **(2003)** | 1013  1015 | Candesartan  Placebo | 265  296 | 219  252 | 207  286 | NR  NR |
| **SYMPOXYDEX**  **(2004)** | 28  22 | Carvedilol  Placebo | 1  1 | NR  NR | NR  NR | NR  NR |
| **ENECA**  **(2005)** | 134  126 | Nebivolol  Placebo | 7  7 | NR  NR | NR  NR | NR  NR |
| **CARMEN**  **(2004)** | 191  190  191 | Carvedilol + Placebo  Enalapril + Placebo  Carvedilol + Enalapril | 14  14  14 | 13  14  9 | 19  28  16 | NR  NR  NR |
| **CIBIS III**  **(2005)** | 505  505 | Bisoprolol  Enalapril | 65  73 | 23  32 | 99  92 | 151  157 |
| **Palazzuoli**  **(2005)** | 33  25 | Carvedilol  Placebo | 1  1 | NR  NR | NR  NR | NR  NR |
| **Palazzuoli, b**  **(2005)** | 32  27 | Carvedilol  Placebo | 1  2 | NR  NR | NR  NR | NR  NR |
| **MERIT-HF**  **(2000)** | 1990  2001 | Metoprolol  Placebo | 145  217 | 128  203 | NR  NR | NR  NR |
| **AREA IN-CHF**  **(2009)** | 231  236 | Canrenone  Placebo | 6  12 | 5  8 | 37  43 | NR  NR |
| **SHIFT**  **(2010)** | 3241  3264 | Ivabradine  Placebo | 503  552 | 449  491 | 514  672 | 1231  1356 |
| **EMPHASIS-HF**  **(2011)** | 1364  1373 | Eplerenone  Placebo | 171  213 | 145  185 | 164  253 | 408  491 |
| **Vizzardi**  **(2014)** | 65  65 | Spironolactone  Placebo | 8  8 | 3  8 | 6  24 | NR  NR |
| **PARADIGM-HF**  **(2014)** | 4187  4212 | Sacubitril-Valsartan  Enalapril | 711  835 | 558  693 | 537  658 | NR  NR |
| **SOCRATES-REDUCED**  **(2015)** | 364  92 | Vericiguat  Placebo | 12  3 | 10  3 | 50  16 | NR  NR |
| **COSMIC-HF**  **(2016)** | 296  148 | Omecamtiv mecarbil  Placebo | NR  NR | NR  NR | NR  NR | NR  NR |
| **DAPA-HF**  **(2019)** | 2373  250  2123  2371  258  2113 | Dapagliflozin  - with ARNI  - without ARNI  Placebo  - with ARNI  - without ARNI | 276  24  252  329  27  302 | 227  18  209  273  21  252 | 237  30  207  326  48  278 | NR  NR  NR  NR  NR  NR |
| **DEFINE-HF**  **(2019)** | 131  132 | Dapagliflozin  Placebo | 1  1 | 1  1 | 12  13 | NR  NR |
| **EMPIRE-HF**  **(2020)** | 95  95 | Empagliflozin  Placebo | 0  0 | NR  NR | 1  0 | NR  NR |
| **VICTORIA**  **(2020)** | 2526  2524 | Vericiguat  Placebo | 512  534 | 414  441 | 691  747 | NR  NR |
| **GALACTIC-HF**  **(2020)** | 4120  4112 | Omecamtiv mecarbil  Placebo | 1067  1065 | 808  798 | 1142  1149 | NR  NR |
| **EMPEROR-REDUCED**  **(2020)** | 1863  340  1523  1867  387  1480 | Empagliflozin  - with ARNI  - without ARNI  Placebo  - with ARNI  - without ARNI | 249  32  217  266  53  213 | 187  21  166  202  35  167 | 246  40  206  342  76  266 | 1364  NR  NR  1560  NR  NR |

ARNI: angiotensin receptor-neprilysin inhibitor; CV: cardiovascular; HF: heart failure; HHF: hospitalization for HF; NR: not reported; RCT: randomized controlled trial.

**Table S7. Results of random-effects Bayesian network meta-analysis for the primary endpoint of all-cause death.**

| **Intervention** | **Comparator** | | | | | | | | | | | | | | | |
| --- | --- | --- | --- | --- | --- | --- | --- | --- | --- | --- | --- | --- | --- | --- | --- | --- |
|  | **PLACEBO** | **ARB** | **DIGO**  **+**  **ACEI** | **ACE**  **+**  **ARB** | **ACEI** | **BB** | **ACEI**  **+**  **MRA** | **ACEI**  **+**  **BB** | **ARB**  **+**  **BB** | **ACEI**  **+**  **ARB**  **+**  **BB** | **ACEI**  **+**  **BB**  **+**  **MRA** | **OM**  **+**  **ACEI**  **+**  **BB**  **+**  **MRA** | **VERI**  **+**  **ACEI**  **+**  **BB**  **+**  **MRA** | **IVA**  **+**  **ACEI**  **+**  **BB**  **+**  **MRA** | **SGLT2i**  **+**  **ACEI**  **+**  **BB**  **+**  **MRA** | **ARNI**  **+**  **BB**  **+**  **MRA** |
| **PLACEBO** | | | | | | | | | | | | | | | | |
| HR (95% CrI) | - | 1.20  (0.85-1.70) | 1.20  (0.85-1.80) | 1.20  (0.87-1.90) | 1.20  (1.00-1.50) | 1.60  (1.10-2.40) | 1.70  (1.20-2.70) | 1.80  (1.40-2.40) | 1.90  (1.20-3.40) | 2.00  (1.30-3.00) | 2.30  (1.60-3.60) | 2.40  (1.50-4.30) | 2.50  (1.50-4.50) | 2.50  (1.50-4.60) | 2.60  (1.70-4.50) | 2.70  (1.70-5.00) |
| **ARB** | | | | | | | | | | | | | | | | |
| HR (95% CrI) | 0.86  (0.60-1.20) | - | 1.00  (0.67-1.60) | 1.10  (0.68-1.70) | 1.10  (0.78-1.40) | 1.40  (0.87-2.20) | 1.50  (0.93-2.40) | 1.50  (1.10-2.20) | 1.60  (0.96-3.00) | 1.70  (1.10-2.70) | 2.00  (1.30-3.20) | 2.00  (1.20-3.70) | 2.10  (1.20-3.80) | 2.20  (1.20-3.90) | 2.30  (1.30-3.90) | 2.40  (1.40-4.20) |
| **DIGO+ACEI** | | | | | | | | | | | | | | | | |
| HR (95% CrI) | 0.83  (0.57-1.20) | 0.96  (0.64-1.50) | - | 1.00  (0.66-1.70) | 1.00  (0.74-1.40) | 1.30  (0.84-2.20) | 1.40  (0.89-2.40) | 1.50  (1.10-2.20) | 1.60  (0.92-3.00) | 1.60  (1.00-2.70) | 1.90  (1.20-3.20) | 2.00  (1.20-3.70) | 2.10  (1.20-3.80) | 2.10  (1.20-3.90) | 2.20  (1.30-3.90) | 2.30  (1.30-4.30) |
| **ACEI+ARB** | | | | | | | | | | | | | | | | |
| HR (95% CrI) | 0.81  (0.53-1.10) | 0.95  (0.60-1.50) | 0.99  (0.60-1.50) | - | 1.00  (0.69-1.40) | 1.30  (0.79-2.10) | 1.40  (0.85-2.30) | 1.50  (1.00-2.10) | 1.50  (0.89-2.90) | 1.60  (1.00-2.50) | 1.90  (1.20-3.10) | 1.90  (1.10-3.60) | 2.00  (1.10-3.70) | 2.10  (1.10-3.80) | 2.10  (1.20-3.70) | 2.20  (1.20-4.10) |
| **ACEI** | | | | | | | | | | | | | | | | |
| HR (95% CrI) | 0.82  (0.66-0.96) | 0.95  (0.71-1.30) | 0.99  (0.72-1.40) | 1.00  (0.73-1.40) | - | 1.30  (0.92-1.90) | 1.40  (0.99-2.10) | 1.50  (1.30-1.70) | 1.60  (0.98-2.70) | 1.60  (1.10-2.30) | 1.90  (1.30-2.80) | 1.90  (1.10-3.30) | 2.00  (1.30-3.40) | 2.10  (1.30-3.50) | 2.10  (1.40-3.40) | 2.20  (1.40-3.80) |
| **BB** | | | | | | | | | | | | | | | | |
| HR (95% CrI) | 0.63  (0.41-0.90) | 0.73  (0.46-1.10) | 0.76  (0.46-1.20) | 0.77  (0.47-1.30) | 0.77  (0.54-1.10) | - | 1.10  (0.64-1.80) | 1.10 (0.77-1.60) | 1.20  (0.86-1.80) | 1.20  (0.75-2.00) | 1.50  (0.89-2.40) | 1.50  (0.85-2.70) | 1.60  (0.86-2.80) | 1.60  (0.87-2.90) | 1.70  (0.95-2.90) | 1.70  (0.95-3.20) |
| **ACEI+MRA** | | | | | | | | | | | | | | | | |
| HR (95% CrI) | 0.57  (0.37-0.84) | 0.67  (0.42-1.10) | 0.69  (0.42-1.10) | 0.70  (0.44-1.20) | 0.70  (0.49-1.00) | 0.91  (0.56-1.60) | - | 1.00  (0.71-1.50) | 1.10  (0.61-2.20) | 1.10  (0.69-1.90) | 1.30  (0.81-2.30) | 1.40  (0.78-2.60) | 1.40  (0.80-2.70) | 1.50  (0.80-2.80) | 1.50  (0.87-2.70) | 1.60  (0.89-3.00) |
| **ACEI+BB** | | | | | | | | | | | | | | | | |
| HR (95% CrI) | 0.56  (0.42-0.69) | 0.65  (0.46-0.89) | 0.68  (0.47-0.93) | 0.69  (0.48-0.98) | 0.68  (0.58-0.78) | 0.89  (0.62-1.30) | 0.98  (0.65-1.40) | - | 1.10  (0.66-1.80) | 1.10  (0.78-1.50) | 1.30  (0.94-1.80) | 1.30  (0.86-2.20) | 1.40  (0.88-2.30) | 1.40  (0.89-2.30) | 1.50  (0.98-2.20) | 1.50  (0.97-2.50) |
| **ARB+BB** | | | | | | | | | | | | | | | | |
| HR (95% CrI) | 0.52  (0.29-0.84) | 0.61  (0.33-1.00) | 0.63  (0.33-1.10) | 0.65  (0.35-1.10) | 0.64  (0.38-1.00) | 0.84  (0.56-1.20) | 0.92  (0.46-1.60) | 0.94  (0.55-1.50) | - | 1.00  (0.54-1.80) | 1.20  (0.64-2.10) | 1.30  (0.64-2.40) | 1.30  (0.64-2.50) | 1.30  (0.64-2.60) | 1.40  (0.70-2.60) | 1.40  (0.70-2.80) |
| **ACEI+ARB+BB** | | | | | | | | | | | | | | | | |
| HR (95% CrI) | 0.51  (0.33-0.74) | 0.59  (0.37-0.93) | 0.61  (0.37-0.98) | 0.62  (0.40-0.99) | 0.62  (0.43-0.88) | 0.81  (0.50-1.30) | 0.89  (0.52-1.50) | 0.91  (0.65-1.30) | 0.97  (0.55-1.80) | - | 1.20  (0.75-1.90) | 1.20  (0.71-2.20) | 1.30  (0.72-2.30) | 1.30  (0.72-2.40) | 1.30  (0.80-2.30) | 1.40  (0.79-2.50) |
| **ACEI+BB+MRA** | | | | | | | | | | | | | | | | |
| HR (95% CrI) | 0.43  (0.28-0.63) | 0.50  (0.32-0.78) | 0.52  (0.32-0.83) | 0.53  (0.33-0.86) | 0.53  (0.36-0.74) | 0.69  (0.42-1.10) | 0.76  (0.44-1.20) | 0.77  (0.55-1.10) | 0.82  (0.47-1.60) | 0.85  (0.52-1.30) | - | 1.00  (0.75-1.50) | 1.10  (0.77-1.50) | 1.10  (0.77-1.50) | 1.10  (0.88-1.50) | 1.20  (0.84-1.70) |
| **OM+ACEI+BB+MRA** | | | | | | | | | | | | | | | | |
| HR (95% CrI) | 0.42  (0.23-0.67) | 0.49  (0.27-0.83) | 0.51  (0.27-0.87) | 0.52  (0.28-0.91) | 0.51  (0.30-0.81) | 0.67  (0.37-1.20) | 0.74  (0.38-1.30) | 0.75  (0.46-1.20) | 0.80  (0.41-1.60) | 0.83  (0.45-1.40) | 0.97  (0.68-1.30) | - | 1.00  (0.64-1.60) | 1.10  (0.64-1.70) | 1.10 (0.71-1.60) | 1.20  (0.70-1.80) |
| **VERI+ACEI+BB+MRA** | | | | | | | | | | | | | | | | |
| HR (95% CrI) | 0.40  (0.22-0.66) | 0.47  (0.26-0.81) | 0.49  (0.26-0.84) | 0.50  (0.27-0.89) | 0.49  (0.29-0.78) | 0.64  (0.35-1.20) | 0.71  (0.37-1.30) | 0.72  (0.44-1.10) | 0.77  (0.39-1.60) | 0.80  (0.43-1.40) | 0.94  (0.66-1.30) | 0.96  (0.61-1.60) | - | 1.00  (0.61-1.70) | 1.10  (0.69-1.60) | 1.10  (0.68-1.80) |
| **IVA+ACEI+BB+MRA** | | | | | | | | | | | | | | | | |
| HR (95% CrI) | 0.39  (0.22-0.65) | 0.46  (0.26-0.81) | 0.48  (0.26-0.84) | 0.48  (0.26-0.88) | 0.48  (0.28-0.78) | 0.63  (0.34-1.20) | 0.69  (0.36-1.20) | 0.70  (0.43-1.10) | 0.75  (0.39-1.60) | 0.77  (0.42-1.40) | 0.91  (0.65-1.30) | 0.94  (0.58-1.60) | 0.97  (0.60-1.60) | - | 1.00  (0.67-1.60) | 1.10  (0.66-1.80) |
| **SGLT2i+ACEI+BB+MRA** | | | | | | | | | | | | | | | | |
| HR (95% CrI) | 0.38  (0.22-0.59) | 0.44  (0.26-0.75) | 0.46  (0.26-0.77) | 0.47  (0.27-0.80) | 0.47  (0.29-0.70) | 0.61  (0.34-1.10) | 0.67  (0.37-1.10) | 0.68  (0.45-1.00) | 0.73  (0.39-1.40) | 0.75  (0.43-1.30) | 0.88  (0.68-1.10) | 0.90  (0.61-1.40) | 0.94  (0.62-1.40) | 0.97  (0.62-1.50) | - | 1.00  (0.68-1.60) |
| **ARNI+BB+MRA** | | | | | | | | | | | | | | | | |
| HR (95% CrI) | 0.36  (0.20-0.59) | 0.42  (0.24-0.74) | 0.44  (0.24-0.77) | 0.45  (0.24-0.80) | 0.45  (0.26-0.71) | 0.58  (0.32-1.10) | 0.64  (0.33-1.10) | 0.65  (0.40-1.00) | 0.69  (0.36-1.40) | 0.72  (0.39-1.30) | 0.84  (0.60-1.20) | 0.86  (0.55-1.40) | 0.90  (0.56-1.50) | 0.93  (0.56-1.50) | 0.96  (0.62-1.50) | - |

ACEi: angiotensin converting enzyme inhibitor; ARB: angiotensin receptor blocker; ARNI: angiotensin receptor-neprilysin inhibitor; BB: beta-blocker; CrI: credible interval; DIGO: digoxin; HR: hazard ratio; IVA: ivabradine; MRA: mineralocorticoid receptor antagonist; OM: omecamtiv mecarbil; SGLT2i: sodium-glucose cotransporter 2 inhibitor; VERI: vericiguat.

**Table S8. Results of random-effects Bayesian network meta-analysis for the secondary endpoint of cardiovascular death.**

| **Intervention** | **Comparator** | | | | | | | | | | | | | | | |
| --- | --- | --- | --- | --- | --- | --- | --- | --- | --- | --- | --- | --- | --- | --- | --- | --- |
|  | **PLACEBO** | **ARB** | **DIGO**  **+**  **ACEI** | **ACE**  **+**  **ARB** | **ACEI** | **BB** | **ACEI**  **+**  **MRA** | **ACEI**  **+**  **BB** | **ARB**  **+**  **BB** | **ACEI**  **+**  **ARB**  **+**  **BB** | **ACEI**  **+**  **BB**  **+**  **MRA** | **OM**  **+**  **ACEI**  **+**  **BB**  **+**  **MRA** | **VERI**  **+**  **ACEI**  **+**  **BB**  **+**  **MRA** | **IVA**  **+**  **ACEI**  **+**  **BB**  **+**  **MRA** | **SGLT2i**  **+**  **ACEI**  **+**  **BB**  **+**  **MRA** | **ARNI**  **+**  **BB**  **+**  **MRA** |
| **PLACEBO** | | | | | | | | | | | | | | | | |
| HR (95% CrI) | - | 1.10 (0.74-1.60) | 1.20  (0.79-1.90) | 1.20  (0.78-1.90) | 1.20  (1.00-1.60) | 1.30  (0.86-2.20) | 1.80  (1.10-2.80) | 1.70  (1.30-2.30) | 1.60  (0.96-3.20) | 2.00  (1.30-3.30) | 2.30  (1.50-4.00) | 2.40  (1.30-5.30) | 2.50  (1.50-5.20) | 2.50  (1.50-5.00) | 2.60  (1.60-4.90) | 2.90  (1.70-5.80) |
| **ARB** | | | | | | | | | | | | | | | | |
| HR (95% CrI) | 0.91  (0.62-1.30) | - | 1.10  (0.68-1.80) | 1.10  (0.67-1.90) | 1.10  (0.81-1.60) | 1.20  (0.74-2.10) | 1.60  (0.97-2.70) | 1.60  (1.10-2.30) | 1.50  (0.84-3.10) | 1.80 (1.10-3.20) | 2.10  (1.30-3.80) | 2.20  (1.20-5.20) | 2.30  (1.30-4.90) | 2.30  (1.30-4.70) | 2.40  (1.40-4.70) | 2.60  (1.50-5.50) |
| **DIGO+ACEI** | | | | | | | | | | | | | | | | |
| HR (95% CrI) | 0.83  (0.52-1.30) | 0.91  (0.55-1.50) | - | 1.00 (0.58-1.70) | 1.00  (0.70-1.50) | 1.10 (0.65-1.90) | 1.50  (0.85-2.50) | 1.40  (0.98-2.20) | 1.40  (0.74-2.80) | 1.70  (0.97-3.00) | 1.90  (1.20-3.60) | 2.00 (0.99-4.80) | 2.10  (1.20-4.40) | 2.10  (1.10-4.40) | 2.20  (1.30-4.40) | 2.40  (1.30-5.00) |
| **ACEI+ARB** | | | | | | | | | | | | | | | | |
| HR (95% CrI) | 0.83  (0.52-1.30) | 0.91 (0.54-1.50) | 0.99  (0.58-1.70) | - | 1.00  (0.68-1.50) | 1.10  (0.64-2.00) | 1.50  (0.84-2.50) | 1.40  (0.94-2.20) | 1.30  (0.74-2.80) | 1.70  (0.95-3.00) | 1.90  (1.20-3.60) | 2.00  (0.98-4.80) | 2.10  (1.10-4.60) | 2.10  (1.10-4.40) | 2.20  (1.20-4.40) | 2.40  (1.30-5.10) |
| **ACEI** | | | | | | | | | | | | | | | | |
| HR (95% CrI) | 0.82  (0.64-1.00) | 0.89  (0.63-1.20) | 0.98  (0.68-1.40) | 0.98  (0.67-1.50) | - | 1.10  (0.73-1.70) | 1.40  (0.98-2.10) | 1.40  (1.20-1.70) | 1.30  (0.82-2.40) | 1.60  (1.10-2.60) | 1.90  (1.30-3.00) | 1.90  (1.10-4.00) | 2.10  (1.30-4.00) | 2.00  (1.20-3.90) | 2.20  (1.40-3.80) | 2.40  (1.50-4.50) |
| **BB** | | | | | | | | | | | | | | | | |
| HR (95% CrI) | 0.74  (0.45-1.20) | 0.82  (0.47-1.40) | 0.90  (0.51-1.50) | 0.90  (0.51-1.60) | 0.91  (0.60-1.40) | - | 1.30  (0.74-2.30) | 1.30  (0.83-2.00) | 1.20  (0.87-1.90) | 1.50  (0.85-2.70) | 1.70  (1.00-3.20) | 1.80  (0.87-3.90) | 1.90  (1.00-4.00) | 1.90  (0.98-3.90) | 2.00  (1.10-3.90) | 2.20  (1.20-4.40) |
| **ACEI+MRA** | | | | | | | | | | | | | | | | |
| HR (95% CrI) | 0.57  (0.36-0.87) | 0.62  (0.37-1.00) | 0.68  (0.40-1.20) | 0.68  (0.40-1.20) | 0.69  (0.47-1.00) | 0.75  (0.44-1.40) | - | 0.96  (0.66-1.50) | 0.92  (0.51-1.90) | 1.10  (0.65-2.10) | 1.30  (0.80-2.40) | 1.30  (0.66-3.20) | 1.40  (0.79-3.10) | 1.40  (0.77-3.00) | 1.50  (0.86-2.90) | 1.60  (0.90-3.40) |
| **ACEI+BB** | | | | | | | | | | | | | | | | |
| HR (95% CrI) | 0.59  (0.43-0.75) | 0.64 (0.43-0.90) | 0.71  (0.46-1.00) | 0.71  (0.45-1.10) | 0.71 (0.59-0.83) | 0.78  (0.50-1.20) | 1.00  (0.66-1.50) | - | 0.95  (0.57-1.70) | 1.20  (0.79-1.70) | 1.30  (0.97-2.00) | 1.40  (0.80-2.70) | 1.50  (0.94-2.70) | 1.50  (0.90-2.60) | 1.50  (1.00-2.60) | 1.70  (1.10-3.00) |
| **ARB+BB** | | | | | | | | | | | | | | | | |
| HR (95% CrI) | 0.62  (0.31-1.00) | 0.67  (0.33-1.20) | 0.74  (0.35-1.30) | 0.74  (0.36-1.40) | 0.75  (0.41-1.20) | 0.82  (0.53-1.20) | 1.10  (0.52-2.00) | 1.10  (0.58-1.80) | - | 1.20  (0.60-2.30) | 1.40  (0.72-2.80) | 1.40  (0.59-3.30) | 1.50  (0.74-3.40) | 1.50  (0.71-3.40) | 1.60  (0.78-3.40) | 1.80  (0.83-3.80) |
| **ACEI+ARB+BB** | | | | | | | | | | | | | | | | |
| HR (95% CrI) | 0.50  (0.30-0.77) | 0.55  (0.31-0.90) | 0.60  (0.34-1.00) | 0.60  (0.33-1.10) | 0.61  (0.39-0.91) | 0.67  (0.37-1.20) | 0.88  (0.48-1.50) | 0.85  (0.58-1.30) | 0.82  (0.43-1.70) | - | 1.10  (0.71-2.10) | 1.20  (0.58-2.70) | 1.20  (0.71-2.60) | 1.20  (0.68-2.50) | 1.30  (0.75-2.50) | 1.40  (0.78-3.00) |
| **ACEI+BB+MRA** | | | | | | | | | | | | | | | | |
| HR (95% CrI) | 0.43  (0.25-0.65) | 0.48  (0.26-0.76) | 0.53  (0.28-0.84) | 0.52  (0.28-0.87) | 0.53  (0.33-0.76) | 0.58  (0.32-0.99) | 0.77  (0.41-1.30) | 0.74  (0.49-1.00) | 0.71  (0.36-1.40) | 0.88  (0.49-1.40) | - | 1.00  (0.65-1.60) | 1.10  (0.78-1.60) | 1.10  (0.75-1.60) | 1.20  (0.86-1.50) | 1.30  (0.86-1.80) |
| **OM+ACEI+BB+MRA** | | | | | | | | | | | | | | | | |
| HR (95% CrI) | 0.44  (0.22-0.72) | 0.48  (0.23-0.85) | 0.53  (0.25-0.94) | 0.53  (0.25-0.97) | 0.54  (0.29-0.86) | 0.58  (0.28-1.10) | 0.77  (0.37-1.40) | 0.75  (0.42-1.20) | 0.71  (0.33-1.50) | 0.88  (0.43-1.60) | 1.00  (0.69-1.40) | - | 1.10  (0.66-1.90) | 1.10  (0.64-1.80) | 1.20  (0.73-1.80) | 1.30  (0.74-2.10) |
| **VERI+ACEI+BB+MRA** | | | | | | | | | | | | | | | | |
| HR (95% CrI) | 0.40  (0.19-0.65) | 0.44  (0.20-0.76) | 0.48  (0.23-0.85) | 0.48  (0.22-0.88) | 0.49  (0.25-0.78) | 0.53  (0.25-0.99) | 0.70  (0.32-1.30) | 0.68  (0.37-1.20) | 0.65  (0.29-1.30) | 0.81  (0.38-1.40) | 0.92  (0.62-1.30) | 0.83  (0.39-1.40) | - | 1.00  (0.58-1.60) | 1.10  (0.65-1.60) | 1.10  (0.66-1.90) |
| **IVA+ACEI+BB+MRA** | | | | | | | | | | | | | | | | |
| HR (95% CrI) | 0.40  (0.20-0.67) | 0.44  (0.21-0.76) | 0.48  (0.23-0.87) | 0.48  (0.23-0.89) | 0.49  (0.26-0.80) | 0.53  (0.26-1.00) | 0.71  (0.33-1.30) | 0.68  (0.38-1.10) | 0.65  (0.30-1.40) | 0.80  (0.40-1.50) | 0.92  (0.63-1.30) | 0.91  (0.49-1.70) | 1.00  (0.61-1.70) | - | 1.10  (0.66-1.70) | 1.20  (0.68-1.90) |
| **SGLT2i+ACEI+BB+MRA** | | | | | | | | | | | | | | | | |
| HR (95% CrI) | 0.38  (0.20-0.61) | 0.41  (0.21-0.70) | 0.46  (0.23-0.79) | 0.46  (0.23-0.82) | 0.46  (0.27-0.71) | 0.50  (0.26-0.92) | 0.67  (0.34-1.20) | 0.65  (0.39-0.99) | 0.62  (0.30-1.30) | 0.76  (0.40-1.30) | 0.87  (0.66-1.20) | 0.87  (0.51-1.60) | 0.94  (0.61-1.50) | 0.95  (0.59-1.50) | - | 1.10  (0.69-1.70) |
| **ARNI+BB+MRA** | | | | | | | | | | | | | | | | |
| HR (95% CrI) | 0.35  (0.17-0.57) | 0.38  (0.18-0.67) | 0.42  (0.20-0.76) | 0.42  (0.20-0.77) | 0.42  (0.22-0.69) | 0.46  (0.23-0.87) | 0.61  (0.29-1.10) | 0.59  (0.33-0.95) | 0.57  (0.26-1.20) | 0.70  (0.34-1.30) | 0.80  (0.55-1.20) | 0.79  (0.43-1.50) | 0.87  (0.53-1.50) | 0.87  (0.52-1.50) | 0.92  (0.57-1.50) | - |

ACEi: angiotensin converting enzyme inhibitor; ARB: angiotensin receptor blocker; ARNI: angiotensin receptor-neprilysin inhibitor; BB: beta-blocker; CrI: credible interval; DIGO: digoxin; HR: hazard ratio; IVA: ivabradine; MRA: mineralocorticoid receptor antagonist; OM: omecamtiv mecarbil; SGLT2i: sodium-glucose cotransporter 2 inhibitor; VERI: vericiguat.

**Table S9. Results of random-effects Bayesian network meta-analysis for the secondary endpoint of hospitalization for heart failure.**

| **Intervention** | **Comparator** | | | | | | | | | | | | | | | |
| --- | --- | --- | --- | --- | --- | --- | --- | --- | --- | --- | --- | --- | --- | --- | --- | --- |
|  | **PLACEBO** | **ARB** | **DIGO**  **+**  **ACEI** | **ACE**  **+**  **ARB** | **ACEI** | **BB** | **ACEI**  **+**  **MRA** | **ACEI**  **+**  **BB** | **ARB**  **+**  **BB** | **ACEI**  **+**  **ARB**  **+**  **BB** | **ACEI**  **+**  **BB**  **+**  **MRA** | **OM**  **+**  **ACEI**  **+**  **BB**  **+**  **MRA** | **VERI**  **+**  **ACEI**  **+**  **BB**  **+**  **MRA** | **IVA**  **+**  **ACEI**  **+**  **BB**  **+**  **MRA** | **SGLT2i**  **+**  **ACEI**  **+**  **BB**  **+**  **MRA** | **ARNI**  **+**  **BB**  **+**  **MRA** |
| **PLACEBO** | | | | | | | | | | | | | | | | |
| HR (95% CrI) | - | 1.60  (1.10-2.40) | 2.00  (1.30-3.40) | 2.30  (1.50-4.40) | 1.50  (1.20-1.90) | 1.20  (0.79-1.90) | 1.90  (1.20-3.30) | 1.90  (1.50-2.70) | 1.60  (0.88-3.20) | 1.90  (1.10-3.10) | 3.40  (2.30-6.00) | 3.50  (2.00-7.50) | 3.90  (2.30-8.50) | 4.40  (2.50-9.40) | 4.50  (2.70-8.30) | 4.10  (2.30-8.80) |
| **ARB** | | | | | | | | | | | | | | | | |
| HR (95% CrI) | 0.63  (0.42-0.90) | - | 1.30  (0.76-2.30) | 1.40  (0.88-2.90) | 0.93  (0.67-1.30) | 0.75  (0.44-1.20) | 1.20  (0.67-2.10) | 1.20  (0.85-1.80) | 1.00  (0.51-2.10) | 1.20  (0.63-2.10) | 2.10  (1.30-3.80) | 2.20  (1.10-4.70) | 2.50  (1.40-5.40) | 2.80  (1.50-6.00) | 2.80  (1.60-5.30) | 2.60  (1.40-5.50) |
| **DIGO+ACEI** | | | | | | | | | | | | | | | | |
| HR (95% CrI) | 0.50  (0.29-0.75) | 0.80  (0.44-1.30) | - | 1.10  (0.67-2.30) | 0.75  (0.47-1.10) | 0.60  (0.32-1.00) | 0.94  (0.49-1.70) | 0.98  (0.61-1.50) | 0.83  (0.37-1.70) | 0.97  (0.46-1.70) | 1.70  (0.98-3.10) | 1.80  (0.87-3.80) | 2.00  (1.00-4.30) | 2.20  (1.10-4.80) | 2.20  (1.20-4.30) | 2.10  (1.00-4.40) |
| **ACEI+ARB** | | | | | | | | | | | | | | | | |
| HR (95% CrI) | 0.44  (0.23-0.66) | 0.69  (0.35-1.10) | 0.87  (0.44-1.50) | - | 0.65  (0.37-0.94) | 0.52  (0.26-0.88) | 0.82  (0.39-1.50) | 0.85  (0.49-1.30) | 0.72  (0.29-1.40) | 0.84  (0.38-1.40) | 1.50 (0.79-2.60) | 1.50  (0.71-3.10) | 1.70  (0.85-3.60) | 1.90  (0.90-3.90) | 1.90  (0.93-3.60) | 1.80  (0.82-3.70) |
| **ACEI** | | | | | | | | | | | | | | | | |
| HR (95% CrI) | 0.67  (0.52-0.82) | 1.10  (0.76-1.50) | 1.30  (0.91-2.10) | 1.50  (1.10-2.70) | - | 0.81  (0.53-1.20) | 1.30  (0.79-2.00) | 1.30  (1.10-1.60) | 1.10  (0.58-2.00) | 1.30  (0.74-2.00) | 2.30  (1.60-3.60) | 2.40  (1.40-4.60) | 2.70  (1.60-5.20) | 3.00  (1.70-5.80) | 3.00  (1.80-5.10) | 2.80  (1.60-5.40) |
| **BB** | | | | | | | | | | | | | | | | |
| HR (95% CrI) | 0.84  (0.54-1.30) | 1.30  (0.80-2.30) | 1.70  (0.98-3.10) | 1.90  (1.10-3.90) | 1.20  (0.85-1.90) | - | 1.60  (0.87-3.00) | 1.60  (1.10-2.60) | 1.40  (0.84-2.20) | 1.60  (0.84-2.80) | 2.90  (1.70-5.30) | 2.90  (1.50-6.50) | 3.30  (1.80-7.40) | 3.70  (1.90-8.10) | 3.70  (2.10-7.30) | 3.50  (1.80-7.60) |
| **ACEI+MRA** | | | | | | | | | | | | | | | | |
| HR (95% CrI) | 0.54  (0.30-0.86) | 0.85  (0.47-1.50) | 1.10  (0.58-2.00) | 1.20  (0.68-2.60) | 0.79  (0.49-1.30) | 0.64  (0.34-1.10) | - | 1.00  (0.63-1.70) | 0.88  (0.39-1.90) | 1.00  (0.48-1.90) | 1.80  (1.00-3.60) | 1.90  (0.92-4.30) | 2.10  (1.10-4.90) | 2.40  (1.20-5.50) | 2.40  (1.20-4.90) | 2.20  (1.10-5.00) |
| **ACEI+BB** | | | | | | | | | | | | | | | | |
| HR (95% CrI) | 0.51  (0.37-0.66) | 0.82  (0.54-1.20) | 1.00  (0.66-1.60) | 1.20  (0.78-2.00) | 0.76  (0.63-0.90) | 0.61  (0.39-0.91) | 0.96  (0.58-1.60) | - | 0.85  (0.43-1.60) | 0.99  (0.57-1.50) | 1.70  (1.30-2.60) | 1.80  (1.00-3.40) | 2.00  (1.30-3.80) | 2.30  (1.30-4.20) | 2.30  (1.40-3.70) | 2.10  (1.20-3.90) |
| **ARB+BB** | | | | | | | | | | | | | | | | |
| HR (95% CrI) | 0.61  (0.31-1.10) | 0.96  (0.48-2.00) | 1.20  (0.60-2.70) | 1.40  (0.71-3.40) | 0.90  (0.49-1.70) | 0.73  (0.45-1.20) | 1.10  (0.53-2.50) | 1.20  (0.64-2.30) | - | 1.20  (0.51-2.40) | 2.10  (1.00-4.60) | 2.10  (0.97-5.40) | 2.40  (1.10-6.30) | 2.70  (1.20-6.80) | 2.70  (1.30-6.30) | 2.50  (1.10-6.50) |
| **ACEI+ARB+BB** | | | | | | | | | | | | | | | | |
| HR (95% CrI) | 0.52  (0.32-0.90) | 0.83  (0.49-1.60) | 1.00  (0.59-2.20) | 1.20  (0.72-2.60) | 0.77  (0.51-1.30) | 0.63  (0.36-1.20) | 0.98  (0.53-2.10) | 1.00  (0.69-1.80) | 0.86  (0.42-2.00) | - | 1.80  (1.10-3.70) | 1.80  (0.98-4.40) | 2.10  (1.10-5.10) | 2.30  (1.20-5.60) | 2.30  (1.30-5.00) | 2.10  (1.10-5.20) |
| **ACEI+BB+MRA** | | | | | | | | | | | | | | | | |
| HR (95% CrI) | 0.29  (0.17-0.44) | 0.47  (0.26-0.76) | 0.59  (0.32-1.00) | 0.68  (0.38-1.30) | 0.44  (0.28-0.63) | 0.35  (0.19-0.59) | 0.55 (0.28-0.97) | 0.57  (0.39-0.80) | 0.48  (0.22-0.95) | 0.56  (0.27-0.93) | - | 1.00  (0.66-1.60) | 1.20  (0.81-1.80) | 1.30  (0.83-2.00) | 1.30  (0.93-1.70) | 1.20  (0.77-1.90) |
| **OM+ACEI+BB+MRA** | | | | | | | | | | | | | | | | |
| HR (95% CrI) | 0.29  (0.13-0.50) | 0.45  (0.21-0.87) | 0.57  (0.26-1.20) | 0.65  (0.32-1.40) | 0.42  (0.22-0.74) | 0.34  (0.15-0.66) | 0.54  (0.23-1.10) | 0.55  (0.30-0.95) | 0.47  (0.18-1.00) | 0.55  (0.23-1.00) | 0.97  (0.61-1.50) | - | 1.10  (0.63-2.20) | 1.30  (0.66-2.40) | 1.30  (0.70-2.10) | 1.20  (0.61-2.20) |
| **VERI+ACEI+BB+MRA** | | | | | | | | | | | | | | | | |
| HR (95% CrI) | 0.25  (0.12-0.43) | 0.40  (0.18-0.73) | 0.51  (0.23-0.96) | 0.58  (0.28-1.20) | 0.38  (0.19-0.62) | 0.30  (0.14-0.56) | 0.48  (0.20-0.92) | 0.49  (0.26-0.79) | 0.42  (0.16-0.88) | 0.49  (0.20-0.88) | 0.86  (0.55-1.20) | 0.89  (0.46-1.60) | - | 1.10  (0.58-2.00) | 1.10  (0.62-1.80) | 1.10  (0.55-1.80) |
| **IVA+ACEI+BB+MRA** | | | | | | | | | | | | | | | | |
| HR (95% CrI) | 0.23  (0.11-0.40) | 0.36  (0.17-0.69) | 0.45  (0.21-0.91) | 0.52  (0.26-1.10) | 0.34  (0.17-0.59) | 0.27  (0.12-0.52) | 0.42  (0.18-0.87) | 0.44  (0.24-0.76) | 0.37  (0.15-0.83) | 0.44  (0.18-0.82) | 0.77  (0.49-1.20) | 0.80  (0.41-1.50) | 0.89  (0.50-1.70) | - | 1.00  (0.56-1.70) | 0.94  (0.49-1.80) |
| **SGLT2i+ACEI+BB+MRA** | | | | | | | | | | | | | | | | |
| HR (95% CrI) | 0.22  (0.12-0.37) | 0.35  (0.19-0.64) | 0.45  (0.23-0.87) | 0.51  (0.28-1.10) | 0.33  (0.20-0.54) | 0.27  (0.14-0.49) | 0.42  (0.20-0.82) | 0.44  (0.27-0.69) | 0.37  (0.16-0.79) | 0.43  (0.20-0.78) | 0.76  (0.57-1.10) | 0.78  (0.47-1.40) | 0.88  (0.56-1.60) | 0.99  (0.59-1.80) | - | 0.92  (0.54-1.70) |
| **ARNI+BB+MRA** | | | | | | | | | | | | | | | | |
| HR (95% CrI) | 0.24  (0.11-0.43) | 0.38  (0.18-0.73) | 0.48  (0.23-0.98) | 0.55  (0.27-1.20) | 0.36  (0.19-0.63) | 0.29  (0.13-0.56) | 0.46  (0.20-0.93) | 0.47  (0.25-0.81) | 0.40  (0.15-0.89) | 0.47  (0.19-0.88) | 0.82  (0.52-1.30) | 0.85  (0.45-1.60) | 0.95  (0.54-1.80) | 1.10  (0.56-2.00) | 1.10  (0.60-1.80) | - |

ACEi: angiotensin converting enzyme inhibitor; ARB: angiotensin receptor blocker; ARNI: angiotensin receptor-neprilysin inhibitor; BB: beta-blocker; CrI: credible interval; DIGO: digoxin; HR: hazard ratio; IVA: ivabradine; MRA: mineralocorticoid receptor antagonist; OM: omecamtiv mecarbil; SGLT2i: sodium-glucose cotransporter 2 inhibitor; VERI: vericiguat.

**Table S10. Results of random-effects Bayesian network meta-analysis for the secondary endpoint of all-cause hospitalization.**

| **Intervention** | **Comparator** | | | | | | | | | | | | | | | |
| --- | --- | --- | --- | --- | --- | --- | --- | --- | --- | --- | --- | --- | --- | --- | --- | --- |
|  | **PLACEBO** | **ARB** | **DIGO**  **+**  **ACEI** | **ACE**  **+**  **ARB** | **ACEI** | **BB** | **ACEI**  **+**  **MRA** | **ACEI**  **+**  **BB** | **ARB**  **+**  **BB** | **ACEI**  **+**  **ARB**  **+**  **BB** | **ACEI**  **+**  **BB**  **+**  **MRA** | **OM**  **+**  **ACEI**  **+**  **BB**  **+**  **MRA** | **VERI**  **+**  **ACEI**  **+**  **BB**  **+**  **MRA** | **IVA**  **+**  **ACEI**  **+**  **BB**  **+**  **MRA** | **SGLT2i**  **+**  **ACEI**  **+**  **BB**  **+**  **MRA** | **ARNI**  **+**  **BB**  **+**  **MRA** |
| **PLACEBO** | | | | | | | | | | | | | | | | |
| HR (95% CrI) | - | 1.20  (0.99-1.60) | 1.20  (0.97-1.70) | 1.20  (0.94-1.70) | 1.10  (0.98-1.30) | 1.20 (0.93-1.50) | 1.30  (0.97-1.80) | 1.30  (1.10-1.70) | 1.30  (0.92-1.80) | 1.60  (1.20-2.30) | 1.60  (1.20-2.20) | NA | NA | 1.70  (1.20-2.70) | 1.80 (1.30-2.8) | 1.90 (1.30-3.00) |
| **ARB** | | | | | | | | | | | | | | | | |
| HR (95% CrI) | 0.84  (0.62-1.00) | - | 1.00  (0.75-1.30) | 1.00  (0.71-1.30) | 0.93  (0.74-1.10) | 0.98  (0.71-1.30) | 1.10 (0.74-1.40) | 1.10  (0.87-1.40) | 1.10  (0.70-1.50) | 1.30  (0.91-1.80) | 1.30  (0.94-1.80) | NA | NA | 1.40  (0.94-2.10) | 1.50  (0.99-2.20) | 1.60  (1.00-2.30) |
| **DIGO+ACEI** | | | | | | | | | | | | | | | | |
| HR (95% CrI) | 0.83  (0.59-1.00) | 0.99  (0.75-1.30) | - | 1.00  (0.70-1.30) | 0.93  (0.71-1.10) | 0.98  (0.69-1.30) | 1.10  (0.73-1.40) | 1.10 (0.85-1.40) | 1.00  (0.68-1.50) | 1.30  (0.90-1.90) | 1.30  (0.91-1.80) | NA | NA | 1.40  (0.92-2.10) | 1.50  (0.97-2.20) | 1.60  (1.00-2.40) |
| **ACEI+ARB** | | | | | | | | | | | | | | | | |
| HR (95% CrI) | 0.83  (0.61-1.10) | 0.98 (0.76-1.40) | 0.99  (0.74-1.40) | - | 0.92  (0.72-1.20) | 0.97  (0.71-1.30) | 1.10 (0.75-1.50) | 1.10  (0.86-1.50) | 1.00  (0.69-1.50) | 1.30  (0.91-1.90) | 1.30  (0.94-1.90) | NA | NA | 1.40  (0.95-2.20) | 1.50 (1.00-2.30) | 1.60  (1.00-2.50) |
| **ACEI** | | | | | | | | | | | | | | | | |
| HR (95% CrI) | 0.90 (0.75-1.00) | 1.10  (0.91-1.40) | 1.10  (0.90-1.40) | 1.10  (0.86-1.40) | - | 1.10  (0.85-1.30) | 1.10  (0.89-1.50) | 1.20  (1.10-1.40) | 1.10  (0.82-1.60) | 1.40  (1.10-1.90) | 1.40  (1.10-1.90) | NA | NA | 1.50  (1.10-2.20) | 1.60  (1.20-2.30) | 1.70  (1.20-2.50) |
| **BB** | | | | | | | | | | | | | | | | |
| HR (95% CrI) | 0.85  (0.65-1.10) | 1.00  (0.78-1.40) | 1.00  (0.77-1.40) | 1.00  (0.75-1.40) | 0.95  (0.76-1.20) | - | 1.10  (0.78-1.50) | 1.10  (0.90-1.40) | 1.10  (0.84-1.30) | 1.30 (0.95-1.90) | 1.30 (0.97-1.90) | NA | NA | 1.50  (0.99-2.20) | 1.50  (1.00-2.30) | 1.60  (1.10-2.50) |
| **ACEI+MRA** | | | | | | | | | | | | | | | | |
| HR (95% CrI) | 0.78  (0.57-1.00) | 0.93  (0.71-1.30) | 0.94  (0.70-1.40) | 0.95  (0.67-1.30) | 0.87  (0.68-1.10) | 0.92  (0.66-1.30) | - | 1.00  (0.80-1.40) | 0.98  (0.65-1.50) | 1.20  (0.85-1.90) | 1.20  (0.87-1.80) | NA | NA | 1.30  (0.89-2.10) | 1.40  (0.93-2.20) | 1.50  (0.99-2.40) |
| **ACEI+BB** | | | | | | | | | | | | | | | | |
| HR (95% CrI) | 0.76  (0.59-0.89) | 0.90  (0.74-1.10) | 0.91  (0.72-1.20) | 0.92  (0.69-1.20) | 0.84  (0.73-0.93) | 0.89  (0.69-1.10) | 0.97  (0.71-1.30) | - | 0.95  (0.66-1.30) | 1.20  (0.90-1.50) | 1.20  (0.95-1.50) | NA | NA | 1.30  (0.94-1.80) | 1.40  (0.98-1.90) | 1.40  (1.00-2.00) |
| **ARB+BB** | | | | | | | | | | | | | | | | |
| HR (95% CrI) | 0.80  (0.56-1.10) | 0.95  (0.68-1.40) | 0.96  (0.67-1.50) | 0.97  (0.65-1.40) | 0.89  (0.64-1.20) | 0.94  (0.74-1.20) | 1.00  (0.68-1.50) | 1.00  0.78-1.50) | - | 1.30  (0.84-2.00) | 1.20  (0.85-1.90) | NA | NA | 1.40  (0.87-2.20) | 1.40  (0.91-2.30) | 1.50  (0.97-2.50) |
| **ACEI+ARB+BB** | | | | | | | | | | | | | | | | |
| HR (95% CrI) | 0.64  (0.43-0.85) | 0.76  (0.55-1.10) | 0.77  (0.54-1.10) | 0.78  (0.52-1.10) | 0.71  (0.52-0.94) | 0.75  (0.52-1.10) | 0.82  (0.54-1.20) | 0.84  (0.65-1.10) | 0.80  (0.51-1.20) | - | 1.00 (0.70-1.40) | NA | NA | 1.10  (0.71-1.70) | 1.10 (0.75-1.70) | 1.20  (0.80-1.90) |
| **ACEI+BB+MRA** | | | | | | | | | | | | | | | | |
| HR (95% CrI) | 0.64  (0.46-0.82) | 0.76  (0.57-1.10) | 0.77  (0.56-1.10) | 0.78  (0.54-1.10) | 0.71  (0.54-0.90) | 0.75  (0.53-1.00) | 0.82  (0.56-1.10) | 0.84  (0.68-1.10) | 0.80  (0.53-1.20) | 1.00  (0.70-1.40) | - | NA | NA | 1.10  (0.86-1.40) | 1.10  (0.91-1.50) | 1.20  (0.95-1.60) |
| **OM+ACEI+BB+MRA** | | | | | | | | | | | | | | | | |
| HR (95% CrI) | NA | NA | NA | NA | NA | NA | NA | NA | NA | NA | NA | NA | NA | NA | NA | NA |
| **VERI+ACEI+BB+MRA** | | | | | | | | | | | | | | | | |
| HR (95% CrI) | NA | NA | NA | NA | NA | NA | NA | NA | NA | NA | NA | NA | NA | NA | NA | NA |
| **IVA+ACEI+BB+MRA** | | | | | | | | | | | | | | | | |
| HR (95% CrI) | 0.59  (0.38-0.82) | 0.70  (0.48-1.10) | 0.70  (0.47-1.10) | 0.71  (0.45-1.00) | 0.65  (0.45-0.90) | 0.69  (0.45-1.00) | 0.75  (0.47-1.10) | 0.77  (0.56-1.10) | 0.73  (0.45-1.10) | 0.91  (0.60-1.40) | 0.91  (0.72-1.20) | NA | NA | - | 1.10  (0.74-1.50) | 1.10 (0.79-1.60) |
| **SGLT2i+ACEI+BB+MRA** | | | | | | | | | | | | | | | | |
| HR (95% CrI) | 0.56  (0.36-0.77) | 0.66  (0.46-1.00) | 0.67  (0.45-1.00) | 0.68  (0.44-1.00) | 0.62  (0.43-0.85) | 0.65  (0.43-0.97) | 0.71  (0.45-1.10) | 0.73  (0.53-1.00) | 0.70  (0.43-1.10) | 0.87  (0.57-1.30) | 0.87  (0.69-1.10) | NA | NA | 0.95  (0.68-1.30) | - | 1.10  (0.76-1.50) |
| **ARNI+BB+MRA** | | | | | | | | | | | | | | | | |
| HR (95% CrI) | 0.52  (0.34-0.74) | 0.63  (0.43-0.96) | 0.63  (0.42-0.97) | 0.64  (0.40-0.95) | 0.58  (0.40-0.81) | 0.62 (0.40-0.92) | 0.67  (0.42-1.00) | 0.69  (0.50-0.96) | 0.66  (0.40-1.00) | 0.82  (0.54-1.30) | 0.82  (0.64-1.10) | NA | NA | 0.90 (0.63-1.30) | 0.94  (0.67-1.30) | - |

ACEi: angiotensin converting enzyme inhibitor; ARB: angiotensin receptor blocker; ARNI: angiotensin receptor-neprilysin inhibitor; BB: beta-blocker; CrI: credible interval; DIGO: digoxin; HR: hazard ratio; IVA: ivabradine; MRA: mineralocorticoid receptor antagonist; OM: omecamtiv mecarbil; SGLT2i: sodium-glucose cotransporter 2 inhibitor; VERI: vericiguat.

**Table S11. Surface under the cumulative ranking area (SUCRA) scores of random-effects network meta-analysis for the endpoint of all-cause death on a scale from 0 to 100 with the leave-one-study out approach.**

| **RCT**  **(year)** | **ARB** | **DIGO**  **+**  **ACEi** | **ACEi** | **ACEi**  **+**  **ARB** | **BB** | **ACEi**  **+**  **MRA** | **ACEi**  **+**  **BB** | **ARB**  **+**  **BB** | **ACEi**  **+**  **ARB**  **+**  **BB** | **ACEi**  **+**  **BB**  **+**  **MRA** | **OM**  **+**  **ACEi**  **+**  **BB**  **+**  **MRA** | **VERI**  **+**  **ACEi**  **+**  **BB**  **+**  **MRA** | **IVA**  **+**  **ACEi**  **+**  **BB**  **+**  **MRA** | **SGLT2i**  **+**  **ACEi**  **+**  **BB**  **+**  **MRA** | **ARNI**  **+**  **BB**  **+**  **MRA** |
| --- | --- | --- | --- | --- | --- | --- | --- | --- | --- | --- | --- | --- | --- | --- | --- |
| **Chalmers**  **(1987)** | 14.7% | 17.5% | 18.6% | 19.4% | 39.7% | 47.6% | 48.1% | 58.0% | 58.2% | 70.5% | 73.8% | 78.1% | 80.3% | 84.8% | 87.5% |
| **CONSENSUS**  **(1987)** | 14.2% | 17.4% | 18.3% | 18.8% | 39.8% | 47.2% | 47.8% | 57.8% | 58.8% | 70.2% | 74.2% | 77.8% | 80.4% | 85.2% | 87.7% |
| **The Captopril-Digoxin Multicenter Research Group**  **(1988)** | 14.7% | 19.2% | 18.4% | 19.7% | 39.3% | 48.1% | 48.7% | 57.2% | 58.6% | 70.3% | 73.7% | 77.7% | 80.4% | 84.5% | 87.0% |
| **Lewis**  **(1989)** | 14.6% | 17.6% | 18.7% | 19.2% | 39.4% | 47.4% | 48.5% | 57.2% | 58.9% | 70.5% | 73.9% | 78.0% | 80.4% | 84.9% | 87.7% |
| **SOLVD**  **(1991)** | 14.9% | 17.9% | 18.7% | 20.0% | 39.5% | 47.9% | 48.8% | 57.7% | 58.7% | 70.3% | 74.0% | 78.0% | 79.7% | 84.4% | 86.5% |
| **SOLVD**  **(1992)** | 15.4% | 17.8% | 19.0% | 19.9% | 38.8% | 47.7% | 49.0% | 56.4% | 58.6% | 70.9% | 74.1% | 78.3% | 80.3% | 84.3% | 87.4% |
| **Colfer**  **(1992)** | 14.6% | 17.7% | 18.7% | 19.0% | 39.6% | 47.4% | 48.2% | 57.6% | 58.6% | 70.2% | 73.5% | 78.2% | 80.5% | 85.4% | 87.7% |
| **MHFT**  **(1993)** | 14.7% | 17.7% | 18.8% | 19.4% | 39.4% | 48.0% | 48.2% | 57.7% | 58.6% | 70.3% | 73.9% | 77.8% | 80.0% | 85.0% | 87.5% |
| **CIBIS**  **(1994)** | 14.7% | 18.0% | 18.6% | 19.5% | 38.8% | 47.6% | 48.7% | 56.8% | 59.0% | 70.9% | 74.4% | 77.4% | 80.3% | 85.1% | 87.5% |
| **CASSIS**  **(1995)** | 14.4% | 17.2% | 18.7% | 19.2% | 39.2% | 48.1% | 48.3% | 56.9% | 58.7% | 70.2% | 74.4% | 77.7% | 80.5% | 85.1% | 87.6% |
| **Beller**  **(1995)** | 14.8% | 17.4% | 18.5% | 19.6% | 39.5% | 47.5% | 48.2% | 57.6% | 58.8% | 70.2% | 73.6% | 77.6% | 80.6% | 85.2% | 87.5% |
| **Brown**  **(1995)** | 14.8% | 18.0% | 18.7% | 19.0% | 39.1% | 47.6% | 48.1% | 57.6% | 58.6% | 70.3% | 74.2% | 78.4% | 80.8% | 84.6% | 87.4% |
| **Dickstein**  **(1995)** | 14.2% | 18.2% | 18.9% | 19.2% | 39.1% | 47.7% | 48.5% | 56.6% | 59.1% | 70.4% | 74.2% | 77.8% | 80.8% | 84.6% | 87.6% |
| **FEST**  **(1995)** | 14.5% | 18.1% | 18.8% | 19.4% | 39.0% | 47.7% | 48.6% | 57.3% | 59.1% | 70.1% | 74.2% | 78.0% | 80.5% | 84.9% | 87.3% |
| **Krum**  **(1995)** | 14.8% | 17.5% | 18.7% | 19.2% | 40.1% | 47.6% | 47.8% | 58.2% | 58.4% | 70.3% | 73.6% | 77.8% | 80.7% | 84.9% | 87.6% |
| **Packer**  **(1996)** | 14.5% | 17.8% | 18.7% | 19.1% | 40.0% | 47.4% | 49.0% | 57.9% | 57.9% | 70.1% | 73.8% | 77.7% | 80.6% | 85.2% | 87.7% |
| **PRECISE**  **(1996)** | 14.8% | 17.9% | 18.7% | 19.4% | 39.4% | 47.5% | 48.2% | 57.3% | 58.5% | 70.2% | 74.2% | 77.7% | 80.9% | 84.8% | 87.5% |
| **MOCHA**  **(1996)** | 13.8% | 18.2% | 18.6% | 19.0% | 39.9% | 47.0% | 48.9% | 58.1% | 58.5% | 69.9% | 73.6% | 78.1% | 80.3% | 85.2% | 88.1% |
| **Colucci**  **(1996)** | 14.5% | 18.0% | 18.7% | 19.4% | 39.2% | 47.8% | 48.1% | 57.1% | 58.2% | 70.3% | 73.4% | 78.2% | 81.0% | 85.4% | 87.8% |
| **ANZ HF Coll GROUP**  **(1997)** | 15.3% | 18.0% | 18.5% | 20.0% | 39.1% | 47.1% | 48.7% | 57.3% | 58.8% | 70.5% | 74.2% | 78.0% | 80.3% | 84.3% | 87.0% |
| **Lang**  **(1997)** | 18.2% | 17.0% | 17.7% | 18.5% | 39.5% | 47.3% | 48.3% | 57.8% | 58.3% | 70.5% | 73.9% | 77.8% | 80.3% | 85.1% | 87.3% |
| **Cohn**  **(1997)** | 14.6% | 17.9% | 18.5% | 19.3% | 39.6% | 47.9% | 48.4% | 57.7% | 58.6% | 70.2% | 74.0% | 78.0% | 80.4% | 84.7% | 87.0% |
| **The Digitalis Investigation Group**  **(1997)** | 15.3% | 19.9% | 19.0% | 19.9% | 38.9% | 46.6% | 47.4% | 56.8% | 57.7% | 70.2% | 73.8% | 78.0% | 80.0% | 84.4% | 87.2% |
| **ELITE I**  **(1997)** | 7.1% | 19.1% | 20.5% | 19.8% | 39.9% | 47.6% | 48.0% | 57.8% | 58.9% | 70.5% | 73.9% | 77.9% | 80.7% | 85.3% | 88.3% |
| **Van Veldhuisen**  **(1998)** | 15.0% | 17.4% | 18.6% | 18.9% | 39.5% | 47.7% | 48.5% | 57.7% | 58.8% | 70.4% | 73.6% | 78.0% | 80.5% | 85.0% | 87.6% |
| **CIBIS-II**  **(1999)** | 15.0% | 17.6% | 18.6% | 19.6% | 40.2% | 47,9% | 48.0% | 58.3% | 58.8% | 70.0% | 74.3% | 77.6% | 80.2% | 84.5% | 86.6% |
| **STRETCH**  **(1999)** | 18.4% | 17.2% | 18.0% | 18.7% | 38.8% | 47.4% | 48.5% | 56.7% | 58.3% | 70.6% | 74.4% | 77.8% | 80.7% | 85.1% | 87.3% |
| **Hamroff**  **(1999)** | 14.3% | 18.1% | 18.6% | 18.3% | 39.6% | 47.5% | 48.2% | 57.3% | 58.9% | 70.5% | 74.3% | 78.0% | 80.8% | 85.1% | 87.4% |
| **MERIT-HF Pilot**  **(1999)** | 14.5% | 18.1% | 18.7% | 19.1% | 39.6% | 47.9% | 48.4% | 57.2% | 58.7% | 70.3% | 74.1% | 77.7% | 80.5% | 84.9% | 87.3% |
| **RALES**  **(1999)** | 15.5% | 18.4% | 20.0% | 20.5% | 40.0% | - | 47.8% | 58.1% | 57.5% | 68.9% | 73.1% | 77.6% | 79.5% | 83.9% | 86.3% |
| **Shettigar**  **(1999)** | 14.8% | 17.7% | 18.6% | 19.1% | 39.5% | 48.1% | 47.9% | 57.7% | 58.2% | 70.4% | 74.0% | 77.7% | 80.8% | 85.1% | 87.5% |
| **RESOLVD**  **(2000)** | 15.1% | 18.4% | 19.2% | 19.6% | 35.6% | 48.8% | 49.2% | 53.3% | 59.2% | 70.8% | 74.4% | 78.6% | 80.5% | 86.0% | 88.0% |
| **ELITE II**  **(2000)** | 27.4% | 16.0% | 16.4% | 17.5% | 37.8% | 46.0% | 47.7% | 56.3% | 58.3% | 70.1% | 73.6% | 78.0% | 79.8% | 85.2% | 87.4% |
| **CELICARD**  **(2000)** | 14.5% | 17.7% | 18.6% | 19.3% | 39.1% | 48.1% | 48.1% | 56.9% | 58.5% | 70.6% | 73.8% | 78.3% | 80.6% | 85.1% | 88.3% |
| **MIC**  **(2000)** | 15.0% | 17.4% | 18.7% | 19.6% | 39.4% | 48.1% | 48.7% | 57.7% | 58.7% | 70.1% | 74.1% | 77.8% | 80.0% | 84.7% | 87.4% |
| **SPICE**  **(2000)** | 15.3% | 17.8% | 18.7% | 18.8% | 39.5% | 47.2% | 48.5% | 57.3% | 58.9% | 70.6% | 73.7% | 78.2% | 80.6% | 84.8% | 87.3% |
| **Sturm**  **(2000)** | 14.7% | 17.8% | 18.7% | 19.3% | 39.3% | 47.9% | 48.2% | 56.8% | 59.0% | 70.5% | 74.5% | 78.3% | 79.7% | 85.0% | 87.3% |
| **REPLACE**  **(2001)** | 13,8% | 17.9% | 18.9% | 19.6% | 39.9% | 47.8% | 48.0% | 57.8% | 59.0% | 70.2% | 73.4% | 78.2% | 80.3% | 85.0% | 87.2% |
| **Val-HeFT**  **(2001)** | 13.8% | 16.4% | 17.3% | 44.6% | 36.8% | 44.4% | 45.5% | 55.6% | 58.4% | 68.4% | 71.9% | 75.8% | 78.7% | 83.2% | 85.8% |
| **BEST**  **(2001)** | 11.9% | 18.3% | 18.9% | 18.2% | 36.3% | 43.8% | 49.9% | 51.3% | 59.7% | 70.5% | 73.1% | 79.5% | 84.2% | 89.0% | 92.9% |
| **Dubach**  **(2002)** | 15.4% | 17.5% | 18.5% | 19.8% | 39.2% | 47.0% | 48.1% | 56.8% | 58.3% | 70.6% | 73.9% | 78.7% | 80.7% | 85.2% | 87.8% |
| **De Milliano**  **(2002)** | 14.9% | 17.4% | 18.6% | 20.0% | 39.6% | 47.4% | 48.6% | 57.4% | 58.6% | 70.3% | 74.0% | 78.1% | 80.4% | 84.8% | 87.0% |
| **Cicoria**  **(2002)** | 15.3% | 18.0% | 18.7% | 19,2% | 39,0% | 47.5% | 48.8% | 56.9% | 59.0% | 70.0% | 74.0% | 77.9% | 80.6% | 84.6% | 87.5% |
| **COPERNICUS**  **(2002)** | 14.8% | 18.0% | 18,9% | 19.6% | 40.0% | 48.5% | 47.8% | 58.3% | 58.6% | 70.1% | 73.4% | 77.6% | 79.6% | 84.6% | 87.3% |
| **HEAVEN**  **(2002)** | 14.9% | 18.8% | 19.6% | 19.9% | 40.0% | 48.6% | 50.4% | 47.8% | 60.2% | 71.4% | 74.6% | 78.7% | 81.7% | 86.1% | 88.4% |
| **Mitrovic**  **(2003)** | 14.1% | 17.4% | 18.8% | 19.5% | 39.5% | 47.4% | 48.1% | 58.1% | 59.2% | 70.2% | 74.2% | 77.6% | 80.1% | 85.0% | 87.7% |
| **RESOLVD**  **(2003)** | 15.3% | 17.8% | 18.8% | 18.4% | 39.4% | 47.5% | 48.3% | 57.4% | 59.5% | 70.3% | 73.9% | 77.8% | 80.7% | 84.5% | 87.5% |
| **CHARM-Added**  **(2003)** | 16.5% | 19.7% | 20.8% | 20.4% | 41.5% | 48.9% | 50.2% | 58.3% | 48.9% | 69.8% | 73.9% | 77.1% | 79.4% | 84.1% | 86.2% |
| **CHARM-Alternative**  **(2003)** | 14.4% | 18.2% | 19.0% | 19.1% | 34.8% | 45.4% | 46.6% | 96.0% | 55.8% | 65.8% | 69.7% | 73.6% | 76.1% | 80.0% | 82.6% |
| **SYMPOXYDEX**  **(2004)** | 15.0% | 17.7% | 18.6% | 19.1% | 39.4% | 47.0% | 48.5% | 57.6% | 58.6% | 70.5% | 74.0% | 77.8% | 80.7% | 85.1% | 87.7% |
| **ENECA**  **(2004)** | 14.5% | 17.5% | 18.6% | 19.4% | 39.5% | 47.3% | 48.6% | 57.6% | 58.5% | 70.5% | 74.0% | 78.2% | 80.4% | 85.3% | 87.0% |
| **CARMEN**  **(2004)** | 15.1% | 17.8% | 18.7% | 19.4% | 40.9% | 46.3% | 48.3% | 58.7% | 57.9% | 70.3% | 74.2% | 77.8% | 80.0% | 84.7% | 87.1% |
| **CIBIS III**  **(2005)** | 14.5% | 17.4% | 18.5% | 18.8% | 53.0% | 44.3% | 45.0% | 69.6% | 55.1% | 67.5% | 71.4% | 75.7% | 78.0% | 83.0% | 85.3% |
| **Palazzuoli**  **(2005)** | 14.4% | 17.7% | 18.8% | 19.4% | 39.0% | 47.5% | 48.4% | 56.8% | 58.3% | 70.6% | 74.1% | 78.3% | 81.0% | 85.1% | 87.7% |
| **Palazzuoli, b**  **(2005)** | 15.0% | 17.8% | 18.8% | 18.9% | 39.2% | 47.6% | 48.0% | 57.2% | 59.1% | 70.2% | 73.8% | 78.2% | 80.9% | 85.1% | 87.6% |
| **MERIT-HF**  **(2009)** | 15.1% | 17.7% | 18.6% | 20.2% | 39.6% | 48.1% | 48.4% | 57.9% | 58.9% | 70.4% | 74.3% | 77.8% | 79.7% | 83.5% | 86.6% |
| **AREA IN-CHF**  **(2009)** | 14.6% | 18.0% | 18.9% | 19.8% | 40.5% | 48.8% | 50.1% | 59.3% | 61.2% | 68.3% | 72.0% | 76.3% | 79.0% | 83.8% | 86.5% |
| **SHIFT**  **(2010)** | 15.6% | 18.9% | 20.0% | 20.9% | 41.9% | 50.3% | 51.3% | 60.0% | 61.4% | 73.6% | 77.1% | 80.1% | - | 86.6% | 89.3% |
| **EMPHASIS-HF**  **(2011)** | 15.7% | 18.3% | 19.2% | 20.3% | 41.0% | 49.3% | 51.1% | 57.4% | 59.5% | 68.3% | 73.0% | 76.7% | 78.9% | 82.9% | 85.3% |
| **Vizzardi**  **(2014)** | 14.7% | 17.5% | 18.5% | 19.5% | 39.9% | 46.6% | 47.8% | 57.9% | 57.7% | 70.6% | 74.8% | 78.2% | 80.6% | 84.7% | 87.8% |
| **PARADIGM-HF**  **(2014)** | 15.8% | 18.9% | 19.8% | 20.7% | 42.4% | 50.2% | 51.3% | 61.2% | 61.6% | 74.4% | 77.9% | 81.6% | 83.1% | 88.0% | - |
| **SOCRATES-REDUCED**  **(2015)** | 14.9% | 17.9% | 18.6% | 19.5% | 39.1% | 47.7% | 48.2% | 57.4% | 58.9% | 70.7% | 73.9% | 76.5% | 80.7% | 85.2% | 88.1% |
| **COSMIC-HF**  **(2016)** | 15.0% | 17.7% | 18.8% | 19.1% | 39.9% | 47.9% | 48.8% | 57.7% | 59.0% | 70.7% | 71.3% | 78.6% | 80.4% | 84.7% | 87.5% |
| **DAPA-HF**  **(2019)** | 15.4% | 17.5% | 18.7% | 19.5% | 40.3% | 47.4% | 49.1% | 58.9% | 59.4% | 71.7% | 75.0% | 78.2% | 80.4% | 78.3% | 87.1% |
| **DEFINE-HF**  **(2019)** | 14.4% | 17.7% | 18.8% | 19.2% | 40.1% | 47.7% | 48.3% | 57.9% | 58.5% | 70.1% | 74.3% | 77.4% | 80.8% | 84.6% | 87.2% |
| **EMPIRE-HF**  **(2020)** | 14.5% | 17.7% | 18.7% | 19.5% | 39.1% | 47.6% | 48.5% | 57.6% | 59.1% | 70.4% | 74.0% | 77.7% | 80.5% | 85.0% | 87.2% |
| **VICTORIA**  **(2020)** | 14.9% | 17.8% | 19.3% | 19.5% | 40.0% | 47.3% | 48.9% | 57.9% | 58.7% | 70.1% | 73.3% | 81.4% | 78.9% | 83.0% | 85.6% |
| **GALACTIC-HF**  **(2020)** | 15.3% | 18.0% | 19.0% | 19.8% | 39.8% | 47.8% | 48.7% | 57.0% | 58.0% | 68.6% | 87.5% | 75.2% | 77.4% | 81.3% | 83.4% |
| **EMPEROR-REDUCED**  **(2020)** | 15.4% | 17.7% | 18.6% | 19.7% | 39.5% | 47.4% | 48.9% | 57.7% | 59.0% | 70.4% | 73.5% | 77.0% | 79.6% | 86.9% | 85.6% |

ACEi: angiotensin converting enzyme inhibitor; ARB: angiotensin receptor blocker; ARNI: angiotensin receptor-neprilysin inhibitor; BB: beta-blocker; DIGO: digoxin; IVA: ivabradine; MRA: mineralocorticoid receptor antagonist; OM: omecamtiv mecarbil; SGLT2i: sodium-glucose cotransporter 2 inhibitor; VERI: vericiguat.

**Table S12. Results for the meta-regression analyses for all-cause death, cardiovascular death, heart failure hospitalization, and all-cause hospitalization.**

The beta coefficient indicates the change in effect estimates for the mean covariate value.

| **Covariate** | **Beta coefficient** | **95% CI** | **DIC** |
| --- | --- | --- | --- |
| **All-cause death** | | | |
| Year of publication (rf-DIC: 87.3) | 0.07 | -0.83;0.97 | 85.2 |
| Age (rf-DIC: 80.0) | -0.15 | -0.04;0.34 | 78.6 |
| Male sex (rf-DIC: 81.4) | 0.09 | -0.40;0.49 | 83.2 |
| Baseline NYHA class III/IV (rf-DIC: 85.2) | -0.30 | -0.66;0.01 | 84.9 |
| Baseline LVEF (rf-DIC: 80.6) | 0.09 | -0.48;0.62 | 78.0 |
| Ischemic HF etiology (rf-DIC: 79.3) | 0.39 | -0.44;1.36 | 77.9 |
| **CV death** | | | |
| Year of publication (rf-DIC: 77.6) | -1.19 | -2.65;0.20 | 76.9 |
| Age (rf-DIC: 78.8) | -0.35 | -1.01;0.27 | 77.8 |
| Male sex (rf-DIC: 74.7) | 0.09 | -0.66;0.64 | 73.9 |
| Baseline NYHA class III/IV (rf-DIC: 78.8) | -0.24 | -0.69;0.15 | 77.4 |
| Baseline LVEF (rf-DIC: 73.4) | 0.33 | -0.58;1.29 | 72.3 |
| Ischemic HF etiology (rf-DIC: 55.3) | 0.60 | -0.57;2.17 | 54.1 |
| **HHF** | | | |
| Year of publication (rf-DIC: 81.6) | 0.20 | -1.07;1.03 | 82.7 |
| Age (rf-DIC: 74.7) | 0.55 | -0.49;0.63 | 75.1 |
| Male sex (rf-DIC: 74.3) | 0.07 | -0.34;0.50 | 74.3 |
| Baseline NYHA class III/IV (rf-DIC: 74.9) | -0.06 | 0.54;0.41 | 75.1 |
| Baseline LVEF (rf-DIC: 70.7) | -0.34 | -1.79;0.97 | 70.6 |
| Ischemic HF etiology (rf-DIC: 76.6) | 0.16 | -0.68;1.34 | 76.1 |
| **All-cause hospitalization** | | | |
| Year of publication (rf-DIC: 63.6) | 0.07 | -0.59;0.69 | 62.2 |
| Age (rf-DIC: 63.1) | 0.24 | -0.46;0.97 | 62.2 |
| Male sex (rf-DIC: 63.3) | 0.10 | -0.34;0.38 | 62.2 |
| Baseline NYHA class III/IV (rf-DIC: 66.4) | -0.03 | -0.68;0.58 | 68.6 |
| Baseline LVEF (rf-DIC: 64.0) | 0.06 | -0.81;1.09 | 69.7 |
| Ischemic HF etiology (rf-DIC: 64.4) | 0.11 | -0.80;0.89 | 65.1 |

CV: cardiovascular; CI: confidence interval; DIC: Deviance Information Criteria; HHF: hospitalization for heart failure; LVEF: left-ventricle ejection fraction; NYHA: New York heart association; rf: reference model.

**Supplementary figures**

**Figure S1. Preferred Reporting Items for Systematic Reviews and Meta-Analysis (PRISMA) flowchart of study selection.**

**Figure S2. Network of the comparisons between different pharmacological treatments for the secondary endpoint of cardiovascular death.**

ACEi: angiotensin converting enzyme inhibitor; ARB: angiotensin receptor blocker; ARNI: angiotensin receptor-neprilysin inhibitor; BB: beta-blocker; DIGO: digoxin; IVA: ivabradine; MRA: mineralocorticoid receptor antagonist; OM: omecamtiv mecarbil; SGLT2i: sodium-glucose cotransporter 2 inhibitor; VERI: vericiguat.


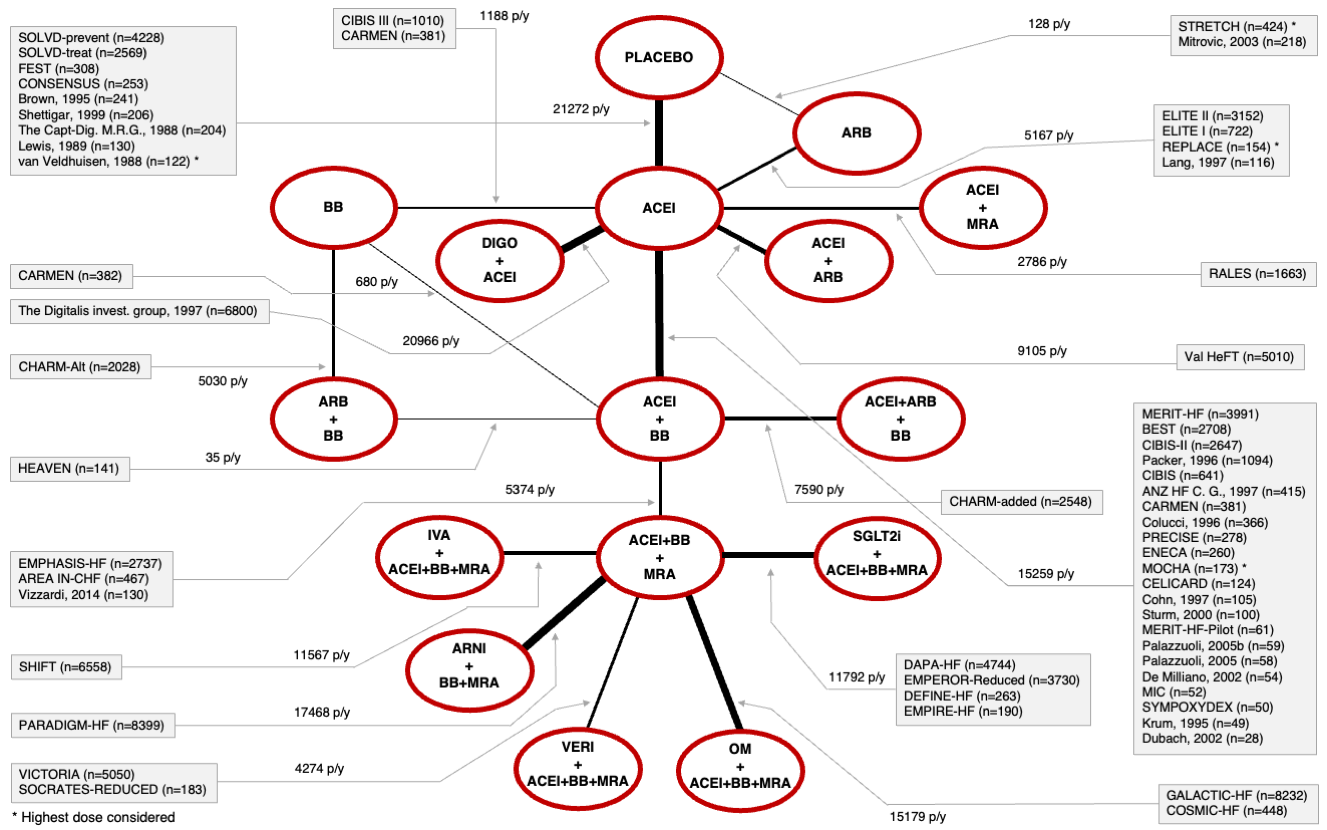


**Figure S3. Network of the comparisons between different pharmacological treatments for the secondary endpoint of hospitalization for heart failure.**

ACEi: angiotensin converting enzyme inhibitor; ARB: angiotensin receptor blocker; ARNI: angiotensin receptor-neprilysin inhibitor; BB: beta-blocker; DIGO: digoxin; IVA: ivabradine; MRA: mineralocorticoid receptor antagonist; OM: omecamtiv mecarbil; SGLT2i: sodium-glucose cotransporter 2 inhibitor; VERI: vericiguat.


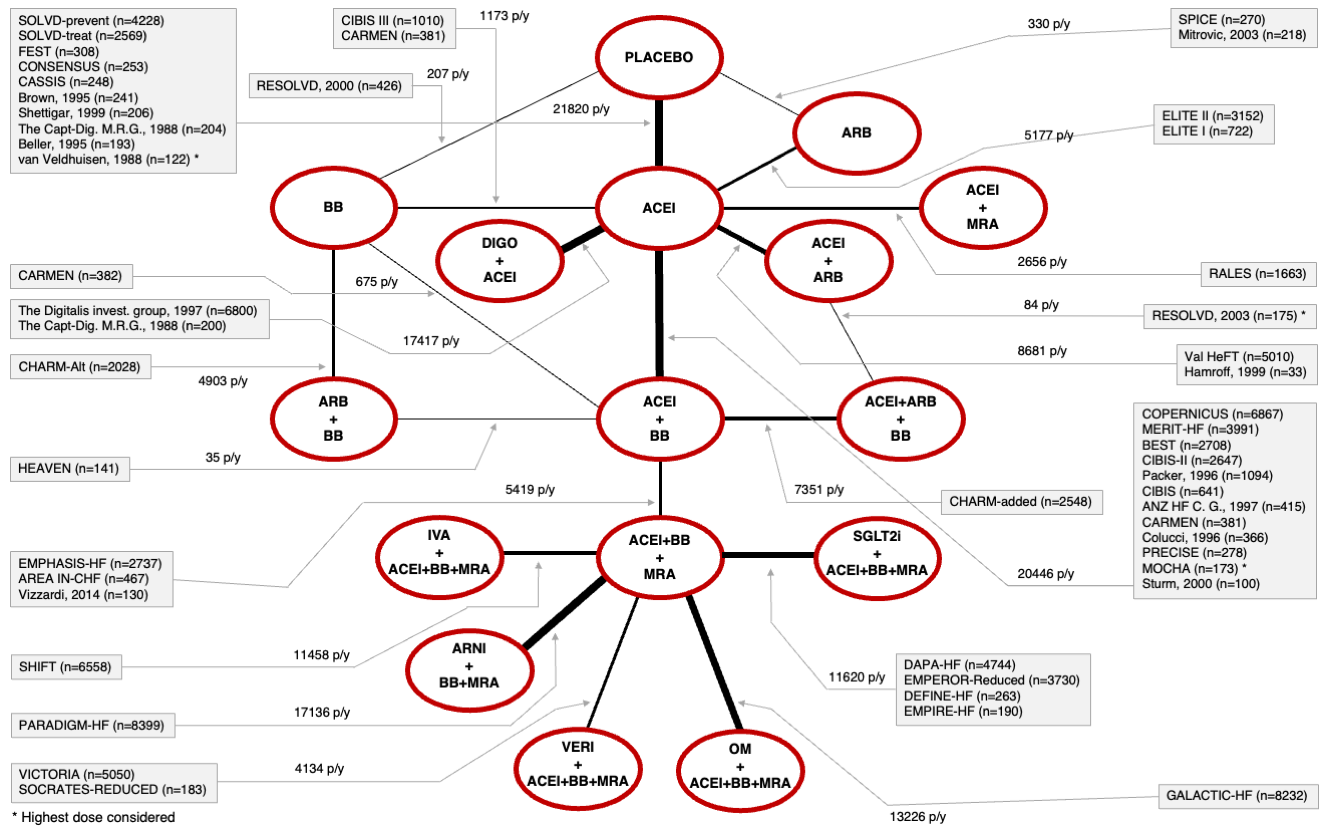


**Figure S4. Network of the comparisons between different pharmacological treatments for the secondary endpoint of all-cause hospitalization.**

ACEi: angiotensin converting enzyme inhibitor; ARB: angiotensin receptor blocker; ARNI: angiotensin receptor-neprilysin inhibitor; BB: beta-blocker; DIGO: digoxin; IVA: ivabradine; MRA: mineralocorticoid receptor antagonist; OM: omecamtiv mecarbil; SGLT2i: sodium-glucose cotransporter 2 inhibitor; VERI: vericiguat.


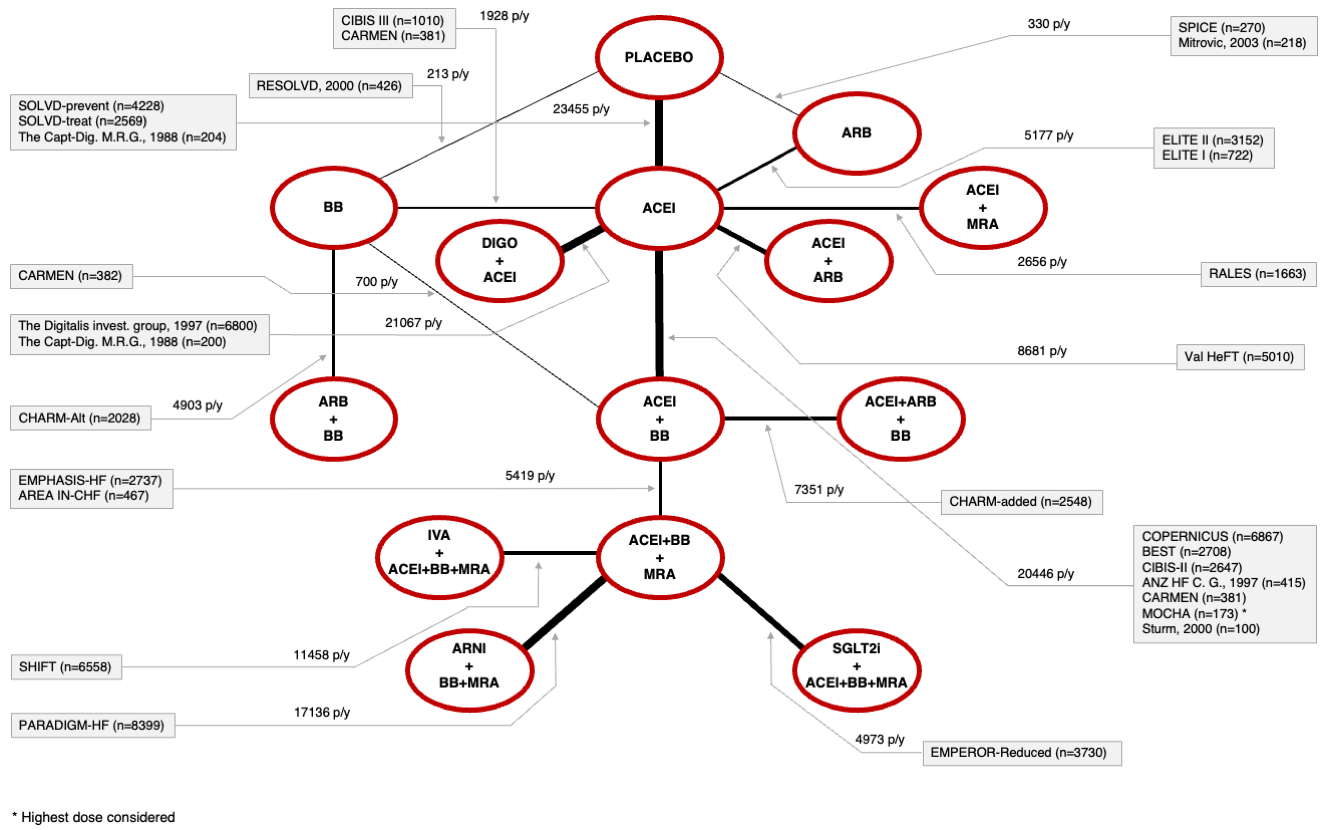


**Figure S5. Results of random-effects frequentist network meta-analysis for all-cause death.**

ACEi: angiotensin converting enzyme inhibitor; ARB: angiotensin receptor blocker; ARNI: angiotensin receptor-neprilysin inhibitor; BB: beta-blocker; CI: confidence interval; DIGO: digoxin; HR: hazard ratio; IVA: ivabradine; MRA: mineralocorticoid receptor antagonist; OM: omecamtiv mecarbil; SGLT2i: sodium-glucose cotransporter 2 inhibitor; VERI: vericiguat.


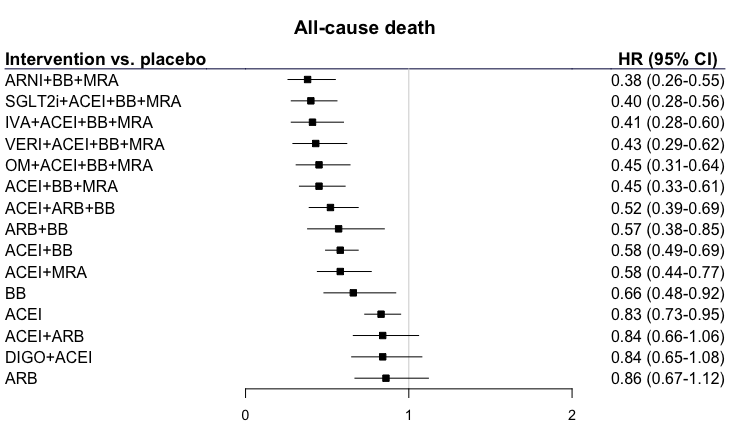


**Figure S6. Results of random-effects frequentist network meta-analysis for cardiovascular death.**

ACEi: angiotensin converting enzyme inhibitor; ARB: angiotensin receptor blocker; ARNI: angiotensin receptor-neprilysin inhibitor; BB: beta-blocker; CI: confidence interval; DIGO: digoxin; HR: hazard ratio; IVA: ivabradine; MRA: mineralocorticoid receptor antagonist; OM: omecamtiv mecarbil; SGLT2i: sodium-glucose cotransporter 2 inhibitor; VERI: vericiguat.


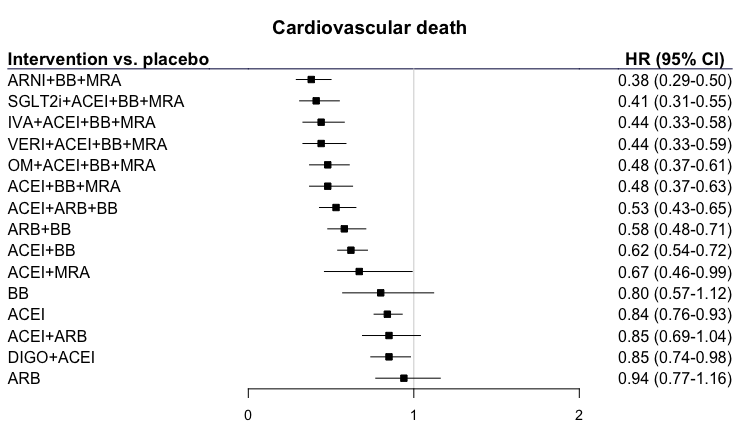


**Figure S7. Results of random-effects frequentist network meta-analysis for heart failure hospitalization.**

ACEi: angiotensin converting enzyme inhibitor; ARB: angiotensin receptor blocker; ARNI: angiotensin receptor-neprilysin inhibitor; BB: beta-blocker; CI: confidence interval; DIGO: digoxin; HR: hazard ratio; IVA: ivabradine; MRA: mineralcorticoid receptor antagonist; OM: omecamtiv mecarbil; SGLT2i: sodium-glucose cotransporter 2 inhibitor; VERI: vericiguat.


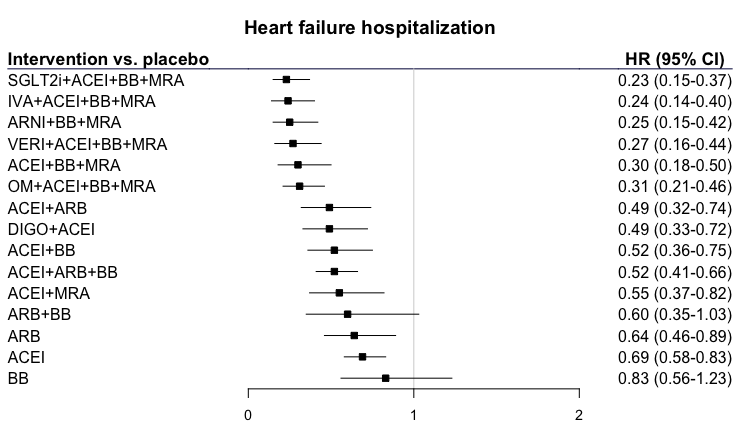


**Figure S8. Results of random-effects frequentist network meta-analysis for all-cause hospitalization.**

ACEi: angiotensin converting enzyme inhibitor; ARB: angiotensin receptor blocker; ARNI: angiotensin receptor-neprilysin inhibitor; BB: beta-blocker; CI: confidence interval; DIGO: digoxin; HR: hazard ratio; IVA: ivabradine; MRA: mineralocorticoid receptor antagonist; OM: omecamtiv mecarbil; SGLT2i: sodium-glucose cotransporter 2 inhibitor; VERI: vericiguat.


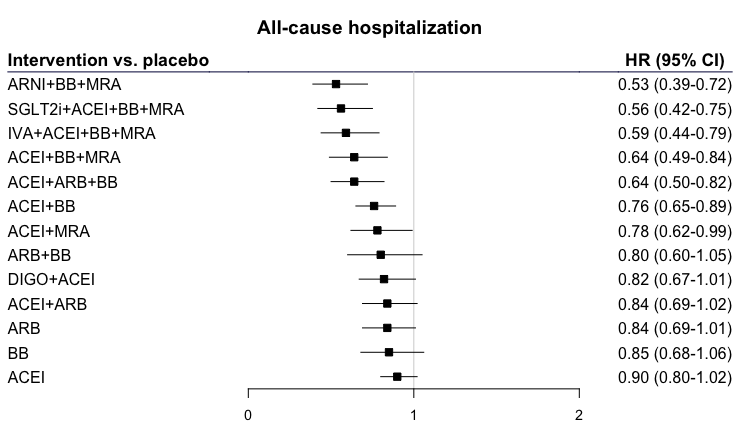


**Figure S9. Risk reduction in all-cause mortality (A), cardiovascular mortality (B), and heart failure hospitalization (C), as calculated by frequentist random-effects network meta-analysis, after distinguishing subgroups of combination therapy with sodium-glucose co-transporter-2 inhibitors according to concomitant use of angiotensin receptor-neprilysin inhibitor.**

ACEi: angiotensin converting enzyme inhibitors; ARB: angiotensin receptor blockers; ARNI: angiotensin receptor-neprilysin inhibitor; BB: beta-blockers; CI: confidence interval; DIGO: digoxin; HR: hazard ratio; IVA: ivabradine; MRA: mineralocorticoid receptor antagonists; OM: omecamtiv mecarbil; SGLT2i: sodium-glucose cotransporter 2 inhibitors; VERI: vericiguat.

**
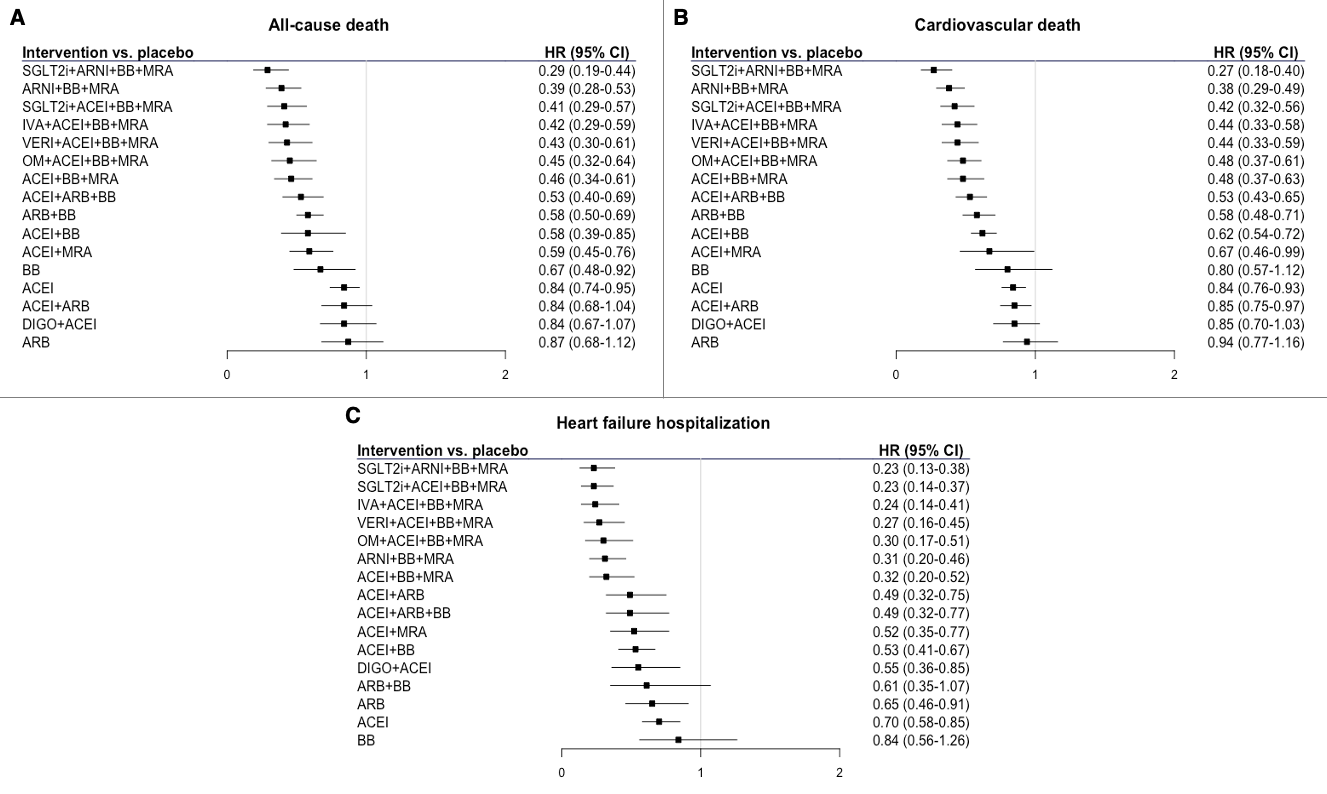
**

**Figure S10. Surface under the cumulative ranking area (SUCRA) scores for all-cause death.**

ACEi: angiotensin converting enzyme inhibitor; ARB: angiotensin receptor blocker; ARNI: angiotensin receptor-neprilysin inhibitor; BB: beta-blocker; DIGO: digoxin; IVA: ivabradine; MRA: mineralcorticoid receptor antagonist; OM: omecamtiv mecarbil; SGLT2i: sodium-glucose cotransporter 2 inhibitor; VERI: vericiguat.


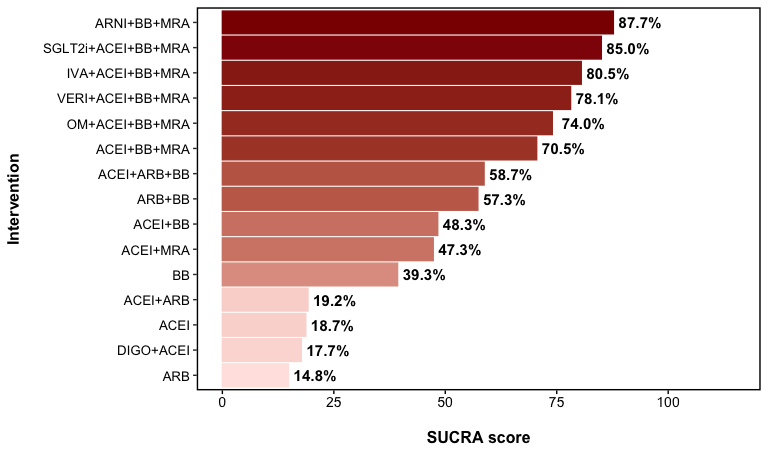


**Figure S11. Surface under the cumulative ranking area (SUCRA) scores for cardiovascular death.**

ACEi: angiotensin converting enzyme inhibitor; ARB: angiotensin receptor blocker; ARNI: angiotensin receptor-neprilysin inhibitor; BB: beta-blocker; DIGO: digoxin; IVA: ivabradine; MRA: mineralcorticoid receptor antagonist; OM: omecamtiv mecarbil; SGLT2i: sodium-glucose cotransporter 2 inhibitor; VERI: vericiguat.


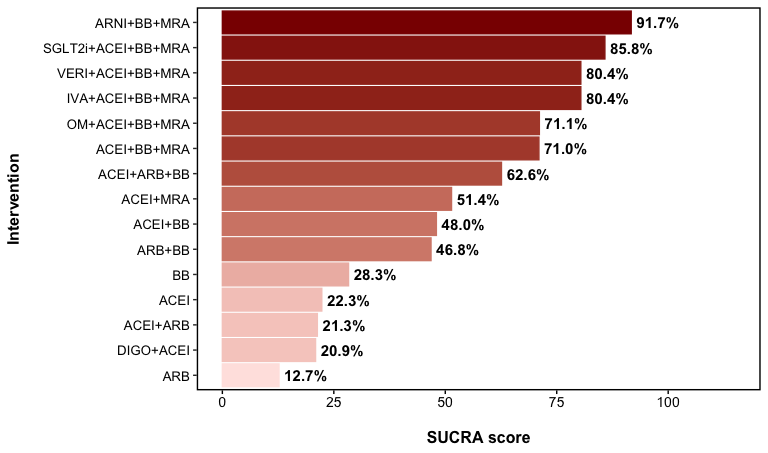


**Figure S12. Surface under the cumulative ranking area (SUCRA) scores for heart failure hospitalization.**

ACEi: angiotensin converting enzyme inhibitor; ARB: angiotensin receptor blocker; ARNI: angiotensin receptor-neprilysin inhibitor; BB: beta-blocker; DIGO: digoxin; IVA: ivabradine; MRA: mineralcorticoid receptor antagonist; OM: omecamtiv mecarbil; SGLT2i: sodium-glucose cotransporter 2 inhibitor; VERI: vericiguat.


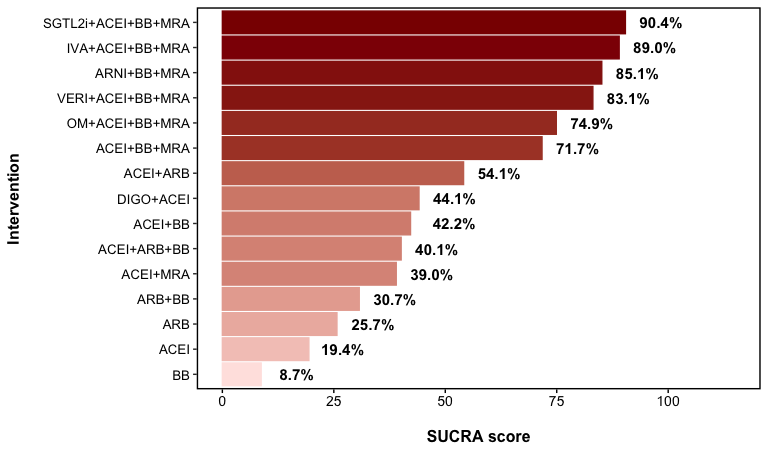


**Figure S13. Surface under the cumulative ranking area (SUCRA) scores for all-cause hospitalization.**

ACEi: angiotensin converting enzyme inhibitor; ARB: angiotensin receptor blocker; ARNI: angiotensin receptor-neprilysin inhibitor; BB: beta-blocker; DIGO: digoxin; IVA: ivabradine; MRA: mineralcorticoid receptor antagonist; OM: omecamtiv mecarbil; SGLT2i: sodium-glucose cotransporter 2 inhibitor; VERI: vericiguat.


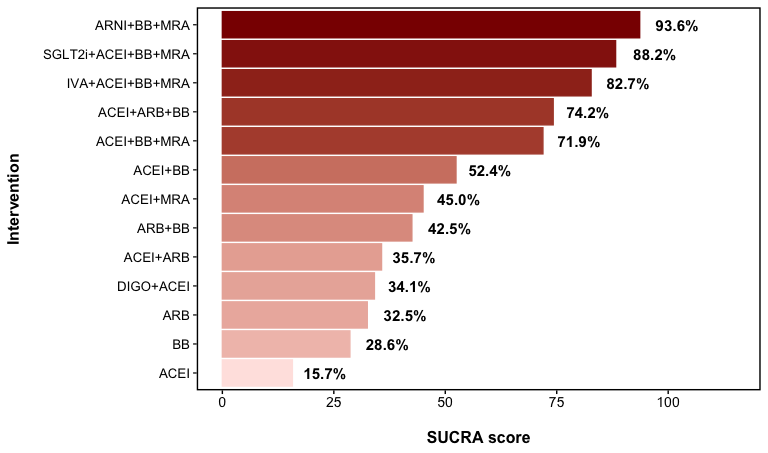


**Figure S14. Comparison-adjusted funnel plots for the primary endpoint of all-cause death.**

ACEi: angiotensin converting enzyme inhibitor; ARB: angiotensin receptor blocker; ARNI: angiotensin receptor-neprilysin inhibitor; BB: beta-blocker; DIGO: digoxin; IVA: ivabradine; MRA: mineralcorticoid receptor antagonist; OM: omecamtiv mecarbil; PSRF: potential scale reduction factor; SGLT2i: sodium-glucose cotransporter 2 inhibitor; VERI: vericiguat.

**
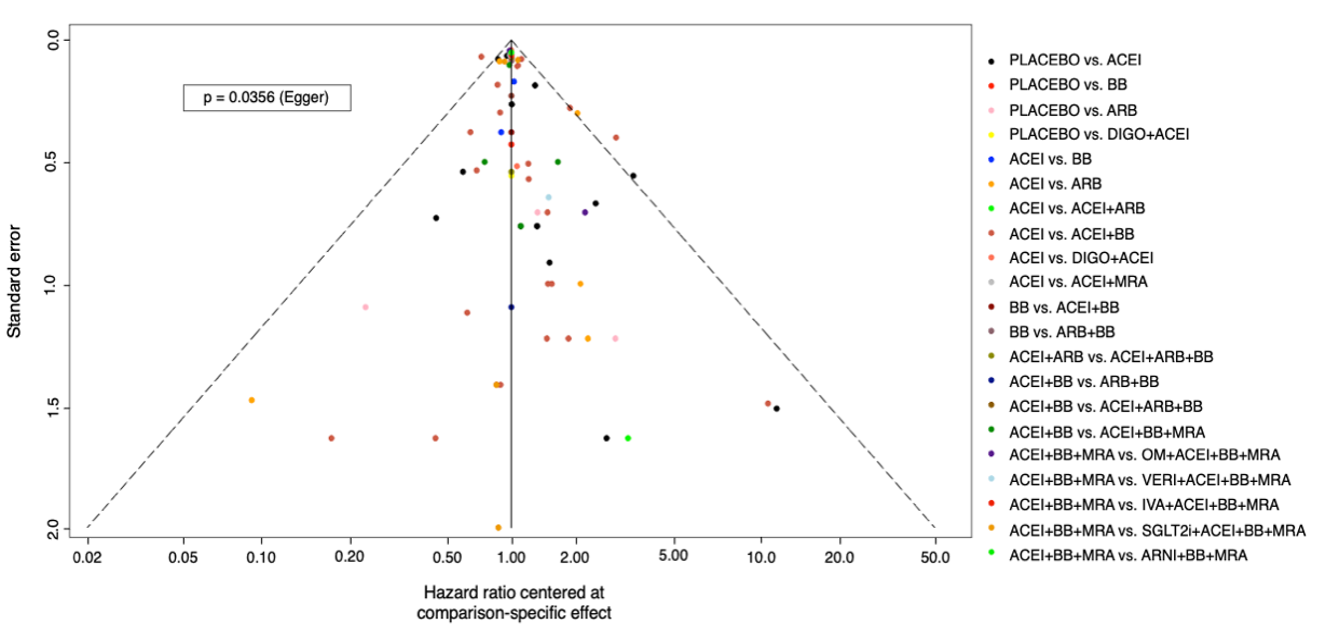
**

**Figure S15. Gelman and Rubin plots to evaluate convergence for all-cause death.**

ACEi: angiotensin converting enzyme inhibitor; ARB: angiotensin receptor blocker; ARNI: angiotensin receptor-neprilysin inhibitor; BB: beta-blocker; DIGO: digoxin; IVA: ivabradine; MRA: mineralcorticoid receptor antagonist; OM: omecamtiv mecarbil; PSRF: potential scale reduction factor; SGLT2i: sodium-glucose cotransporter 2 inhibitor; VERI: vericiguat.


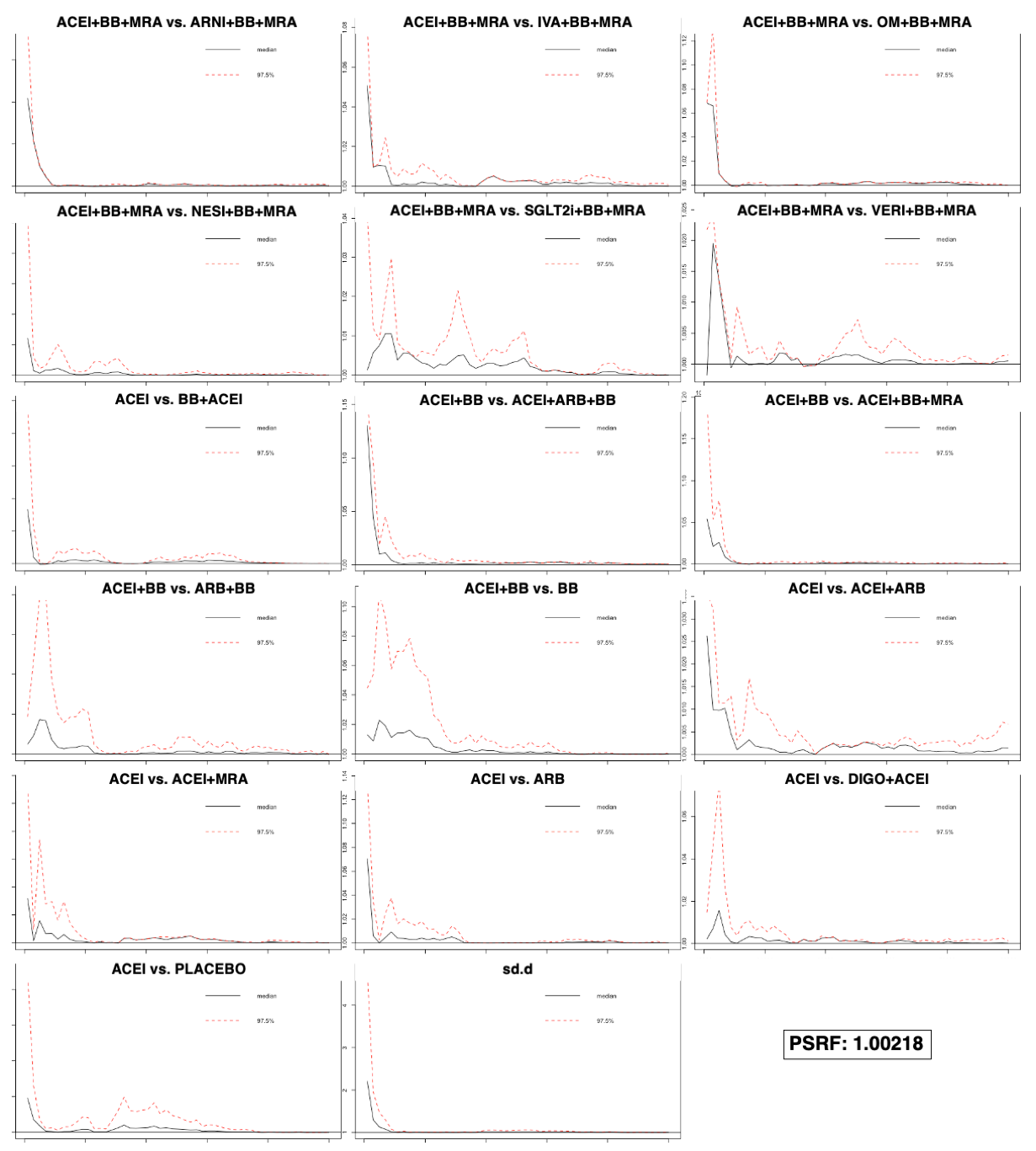


**Figure S16. Node-split analyses for the primary endpoint of all-cause death.**

Each forest plot show the direct, indirect, and network estimates for each direct comparison of the main network. P <0.05 indicates significant inconsistency.

ACEi: angiotensin converting enzyme inhibitor; ARB: angiotensin receptor blocker; ARNI: angiotensin receptor-neprilysin inhibitor; BB: beta-blocker; CrI: credible interval; DIGO: digoxin; HR: hazard ratio; IVA: ivabradine; MRA: mineralocorticoid receptor antagonist; OM: omecamtiv mecarbil; SGLT2i: sodium-glucose cotransporter 2 inhibitor; VERI: vericiguat.


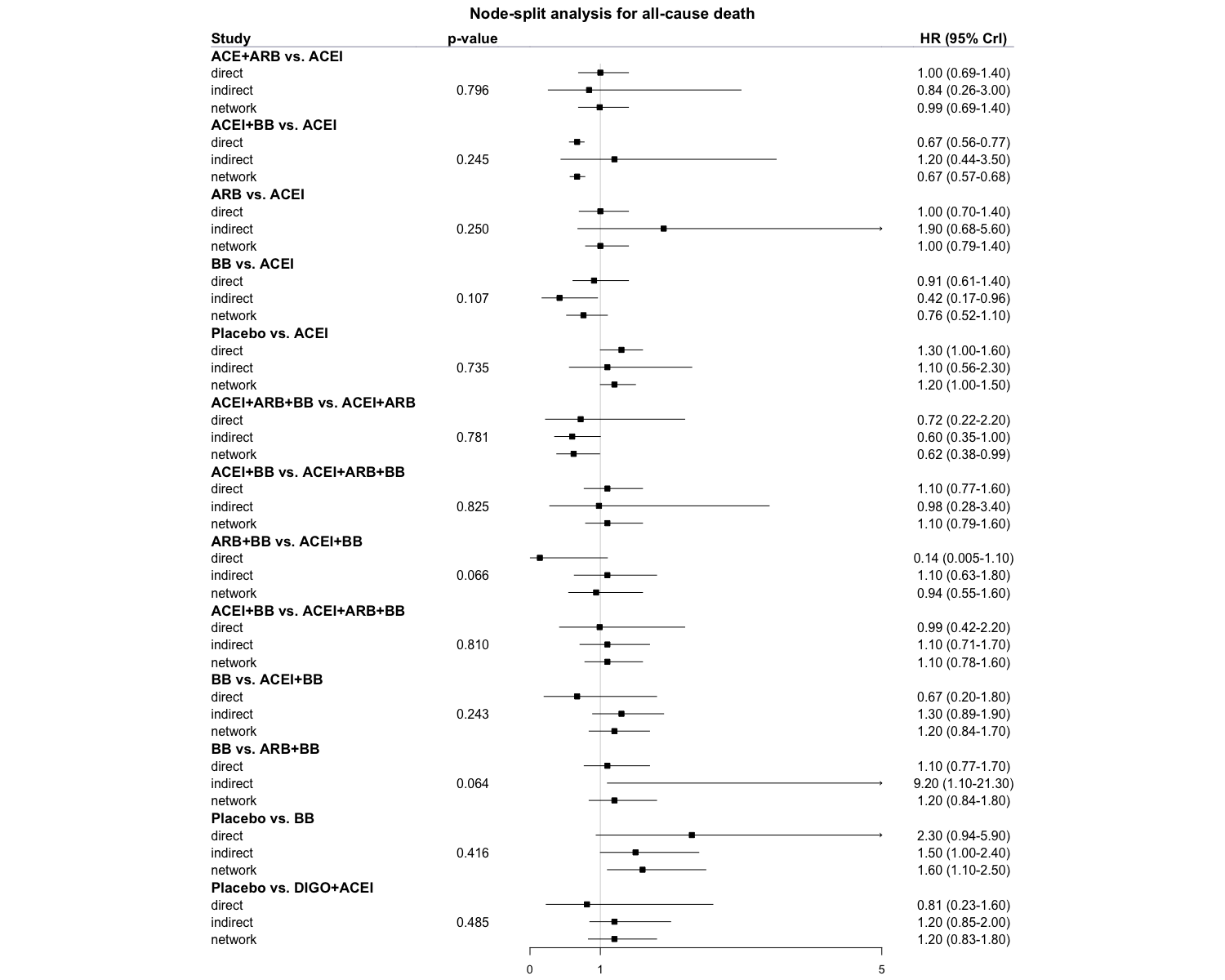


**Figure S17. Node-split analyses for cardiovascular mortality.**

Each forest plot show the direct, indirect, and network estimates for each direct comparison of the main network. P <0.05 indicates significant inconsistency.

ACEi: angiotensin converting enzyme inhibitor; ARB: angiotensin receptor blocker; ARNI: angiotensin receptor-neprilysin inhibitor; BB: beta-blocker; CrI: credible interval; DIGO: digoxin; HR: hazard ratio; IVA: ivabradine; MRA: mineralocorticoid receptor antagonist; OM: omecamtiv mecarbil; SGLT2i: sodium-glucose cotransporter 2 inhibitor; VERI: vericiguat.


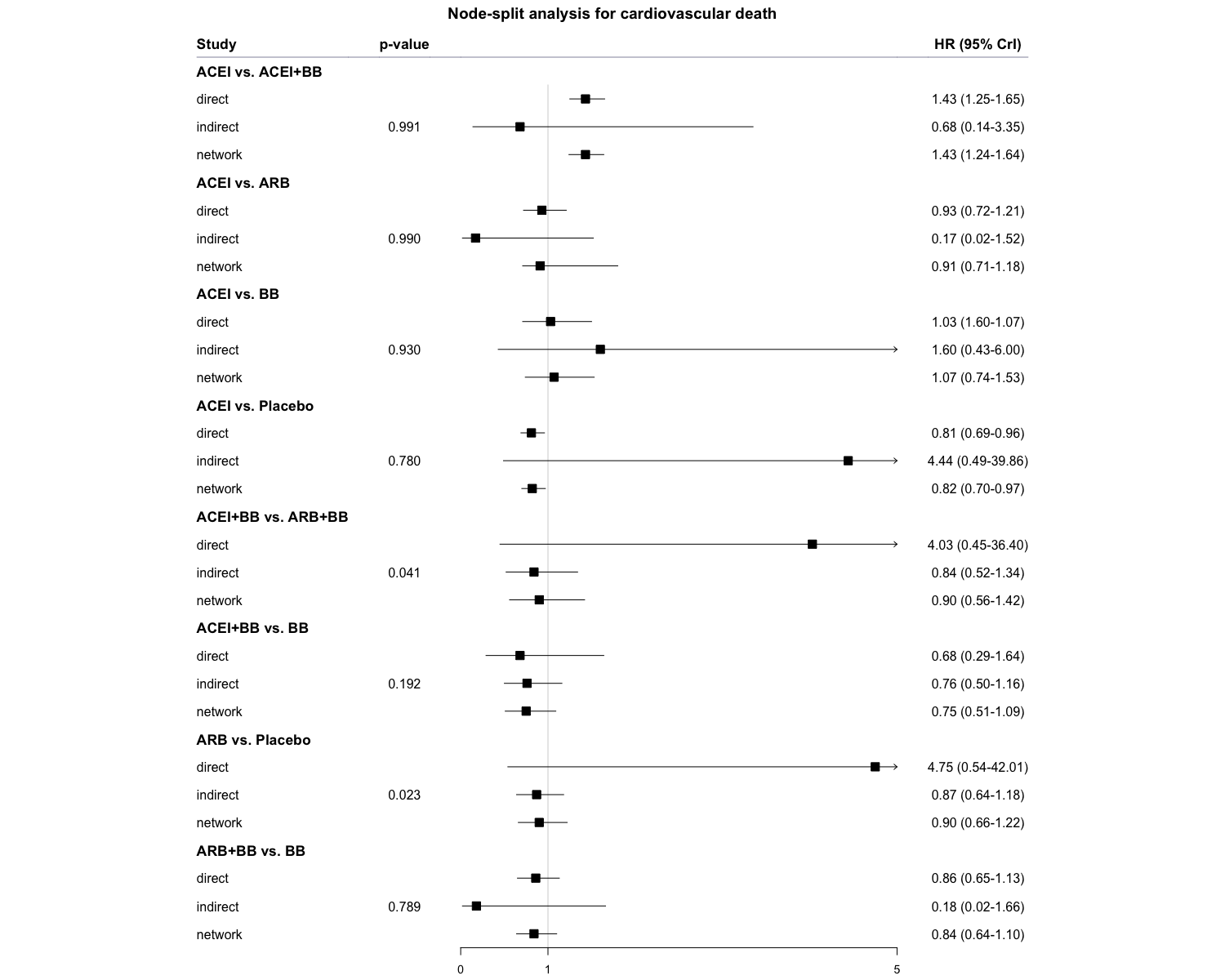


**Figure S18. Node-split analyses for heart failure hospitalization.**

Each forest plot show the direct, indirect, and network estimates for each direct comparison of the main network. P <0.05 indicates significant inconsistency.

ACEi: angiotensin converting enzyme inhibitor; ARB: angiotensin receptor blocker; ARNI: angiotensin receptor-neprilysin inhibitor; BB: beta-blocker; CrI: credible interval; DIGO: digoxin; HR: hazard ratio; IVA: ivabradine; MRA: mineralocorticoid receptor antagonist; OM: omecamtiv mecarbil; SGLT2i: sodium-glucose cotransporter 2 inhibitor; VERI: vericiguat.


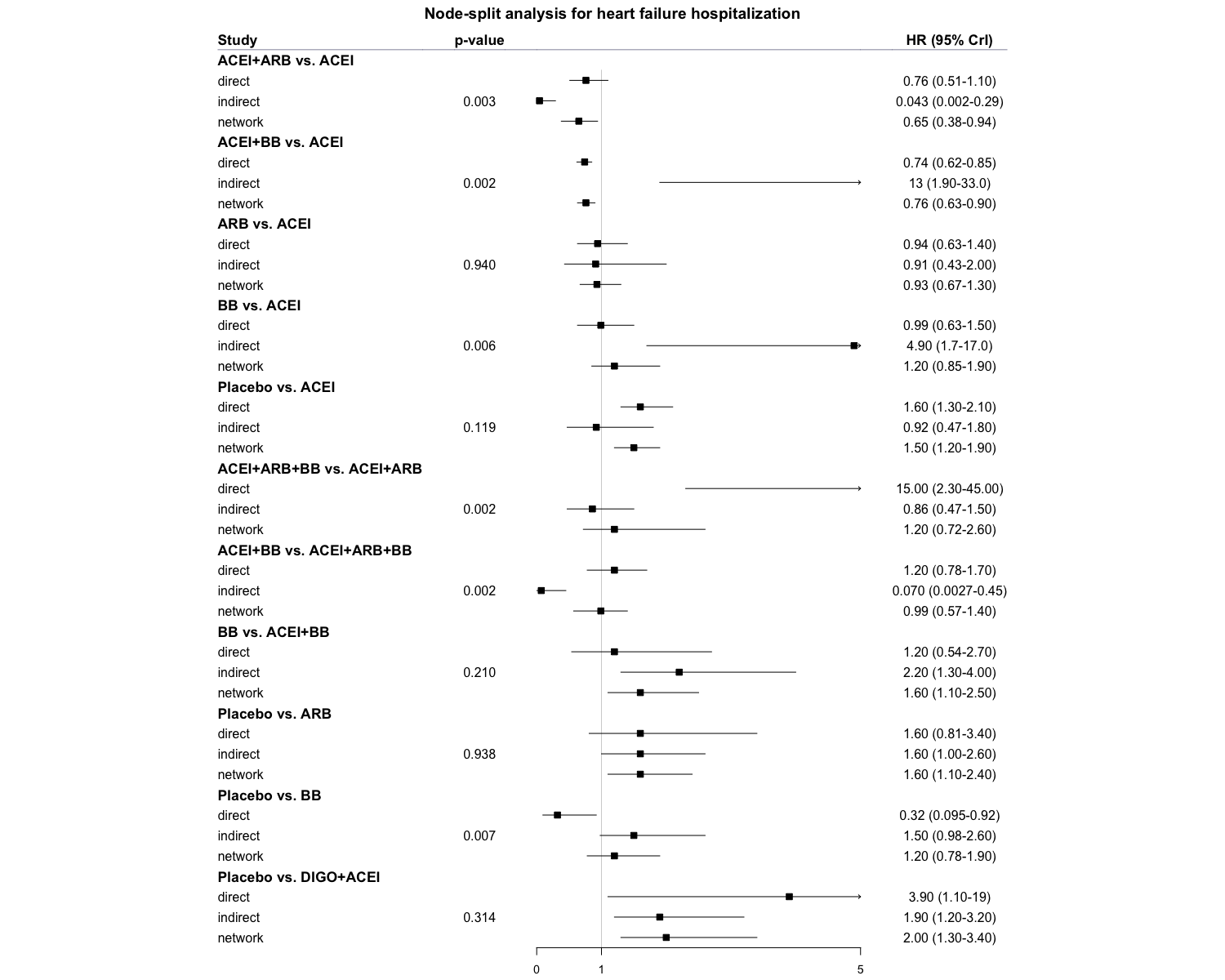


**Figure S19. Node-split analyses for all-cause hospitalization.**

Each forest plot show the direct, indirect, and network estimates for each direct comparison of the main network. P <0.05 indicates significant inconsistency.

ACEi: angiotensin converting enzyme inhibitor; ARB: angiotensin receptor blocker; ARNI: angiotensin receptor-neprilysin inhibitor; BB: beta-blocker; CrI: credible interval; DIGO: digoxin; HR: hazard ratio; IVA: ivabradine; MRA: mineralocorticoid receptor antagonist; OM: omecamtiv mecarbil; SGLT2i: sodium-glucose cotransporter 2 inhibitor; VERI: vericiguat.

**
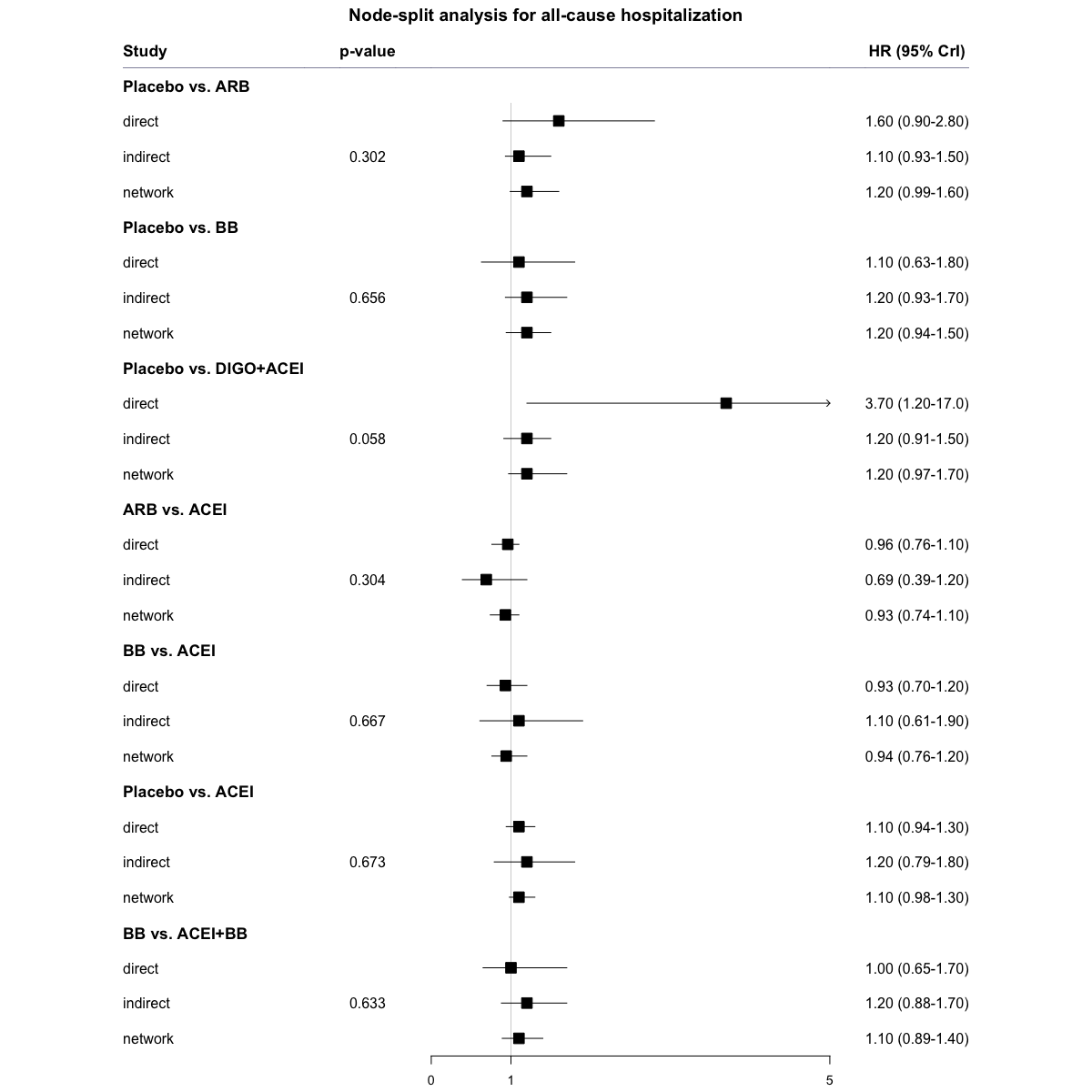
**
